# Host genetic variation impacts microbiome composition across human body sites

**DOI:** 10.1186/s13059-015-0759-1

**Published:** 2015-09-15

**Authors:** Ran Blekhman, Julia K. Goodrich, Katherine Huang, Qi Sun, Robert Bukowski, Jordana T. Bell, Timothy D. Spector, Alon Keinan, Ruth E. Ley, Dirk Gevers, Andrew G. Clark

**Affiliations:** Department of Genetics, Cell Biology, and Development, University of Minnesota, Minneapolis, MN 55455 USA; Department of Ecology, Evolution, and Behavior, University of Minnesota, St. Paul, MN 55108 USA; Department of Molecular Biology and Genetics, Cornell University, Ithaca, NY 14853 USA; Department of Microbiology, Cornell University, Ithaca, NY 14853 USA; Broad Institute of MIT and Harvard, Cambridge, MA 02142 USA; BRC Bioinformatics Facility, Institute of Biotechnology, Cornell University, Ithaca, NY 14853 USA; Department of Twin Research & Genetic Epidemiology, King’s College, London, UK; Department of Biological Statistics and Computational Biology, Cornell University, Ithaca, NY 14853 USA; Current address: Janssen Human Microbiome Institute, Janssen Research and Development, Cambridge, MA 02142 USA

## Abstract

**Background:**

The composition of bacteria in and on the human body varies widely across human individuals, and has been associated with multiple health conditions. While microbial communities are influenced by environmental factors, some degree of genetic influence of the host on the microbiome is also expected. This study is part of an expanding effort to comprehensively profile the interactions between human genetic variation and the composition of this microbial ecosystem on a genome- and microbiome-wide scale.

**Results:**

Here, we jointly analyze the composition of the human microbiome and host genetic variation. By mining the shotgun metagenomic data from the Human Microbiome Project for host DNA reads, we gathered information on host genetic variation for 93 individuals for whom bacterial abundance data are also available. Using this dataset, we identify significant associations between host genetic variation and microbiome composition in 10 of the 15 body sites tested. These associations are driven by host genetic variation in immunity-related pathways, and are especially enriched in host genes that have been previously associated with microbiome-related complex diseases, such as inflammatory bowel disease and obesity-related disorders. Lastly, we show that host genomic regions associated with the microbiome have high levels of genetic differentiation among human populations, possibly indicating host genomic adaptation to environment-specific microbiomes.

**Conclusions:**

Our results highlight the role of host genetic variation in shaping the composition of the human microbiome, and provide a starting point toward understanding the complex interaction between human genetics and the microbiome in the context of human evolution and disease.

**Electronic supplementary material:**

The online version of this article (doi:10.1186/s13059-015-0759-1) contains supplementary material, which is available to authorized users.

## Background

Recent advances in high-throughput sequencing technologies have unveiled wide variability in the microbial communities that coat the human body [1, 2]. There are differences in the microbiota across body sites, which constitute distinct ecological niches [1, 3, 4]. Within each body site, the composition of the microbiome may change rapidly, but community features can remain constant for years [5, 6]. There is great variability in the microbiome across individuals, with some differences associated with chronic conditions, including obesity, diabetes, and inflammatory bowel disease (IBD) [7–12]. Recent studies in germ-free animals have shown that these shifts in the microbiome can have an effect on host traits and could be causal in disease phenotypes [7, 12–14]. Therefore, understanding the factors that impact the composition of the microbiome in healthy individuals is critical to elucidate the role of the microbiome in disease and for development of therapeutics targeting the microbiome.

The composition of the human microbiome is influenced by multiple environmental factors. For example, changes in host diet affect gut microbiome communities at the taxonomic and functional level [5, 15]. In addition, intake of drugs and antibiotics can modulate the gut microbiome [16, 17]. Moreover, studies have shown variation in the gut microbiome can be controlled by interactions with pathogens and parasites [18, 19]. Lastly, social contact and interaction with the environment have also been implicated in shaping the microbial flora in the gut and skin [20–22].

Along with this clear evidence for the influence of environmental factors, there is also support for a host genetic component in structuring of human microbial communities [23]. For example, single nucleotide polymorphisms (SNPs) in the *MEFV* gene are associated with changes in human gut bacterial community structure [24], and IBD-risk loci are associated with changes in gut microbiome composition [25]. Researchers have also shown that a loss-of-function polymorphism in the gene *FUT2*, which is a known risk factor for Crohn’s disease, may modulate energy metabolism of the gut microbiome [26]. Investigating individuals with inflammatory bowel disease, Knights *et al.* have shown that *NOD2* risk allele count is correlated with an increase in the relative abundance of Enterobacteriaceae [27].

In addition to targeted and candidate gene approaches, researchers have also used host genome-wide genetic variation to find interactions with the microbiome. For example, in a recent study using 416 twin pairs to assess the heritability of the microbiome, Goodrich *et al.* identified microbial taxa for which relative abundance is more similar in monozygotic compared to dizygotic twins [14]. In the laboratory mouse, quantitative trait locus (QTL)-mapping approaches have found multiple loci associated with gut microbial community composition, some of which overlap genes involved in immune response [28, 29]. Moreover, researchers have shown that host mitochondrial DNA haplogroups are correlated with the structure of microbiome communities [30]. However, to date, there are no genome-wide studies that attempt to characterize specific genes and pathways in the human genome that shape the composition of the microbiome, although the value of such studies has often been suggested [31, 32].

Here, we performed a genome-wide analysis to identify human genes and pathways correlated with microbiome composition, using data generated by the Human Microbiome Project (HMP). In the last few years, the HMP has sampled and cataloged the microbial diversity across multiple body sites in a few hundred individuals [33]. Since genotype data are not yet available for the individuals included in the HMP study, we extracted host genomic information from the ‘human contamination’ reads in the HMP shotgun metagenomic sequencing. This allowed us to generate genome-wide genetic variation data from 93 individuals, which we then tested for association with the microbiome profiles generated by the HMP.

## Results and discussion

### Mining the human microbiome project data for host reads

First, we scanned and identified the short reads in the metagenomic sequencing data that map to the human genome. By combining these reads across body sites (primarily originating from nares and cheek swabs [33]) for each individual (Additional file 1: Figure S1), we attained a mean depth of coverage of more than 10 reads per base pair per individual (Additional file 1: Figure S2). Combining all 93 individuals, the mean depth of coverage for each site is 1,061 reads (median 1,093), and 99 % of sites are covered at >500x summed across individuals. There is noticeable variability across individuals, although most individuals have a mean coverage in the range of 5x-20x (Additional file 1: Figure S3). We performed genotype calling on these individuals using stringent quality controls and filtering, and identified a final set of 4.2 million high-quality and informative single nucleotide polymorphisms (SNPs), of which 92 % were previously known and found in dbSNP, and were used in subsequent analyses (Additional file 1: Figures S1 to S10). The number of SNPs we identified is in line with previous reports using whole-genome sequencing in humans [34].

### Correlation between host genetic variation and microbiome composition

**Fig. 1:**
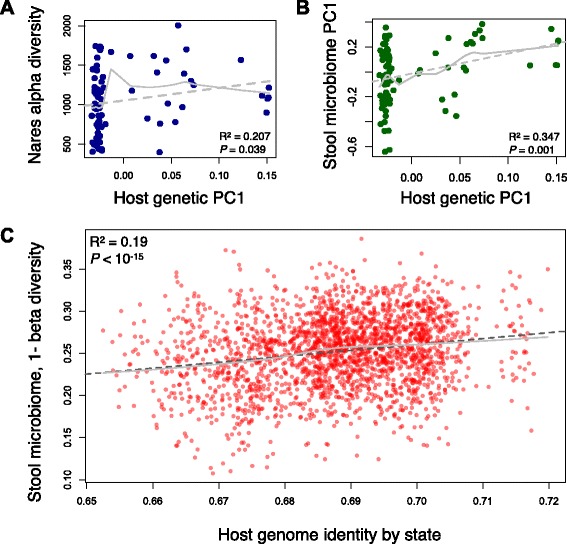
Host genetic variation is correlated with microbiome composition. **a** Correlation of the first PC of host genetic data (*x*-axis) and alpha diversity of the anterior nares microbiome (*y*-axis). **b** Correlation of the first PC of host genetic data (*x*-axis) and first PC of the stool microbiome data (*y*-axis). **c** Identity-by-state between individual pairs calculated from host genome data (*x*-axis) is correlated with stool microbiome beta diversity (*y*-axis), which tabulates the magnitude of pairwise differentiation between the microbiomes of same pair of individuals. In all panels, solid and dashed gray lines represent a linear regression and loess regression fit to the data, respectively

First, we examined the correlation between host genetic variation and the overall diversity of the microbiome. At this point we attempted to identify gross correlation signatures, still without accounting for population structure, and deferring the discussion of mechanistic causes for these correlations until later in the paper. We calculated the coordinates underlying variability in the host genetic data using multidimensional scaling (MDS). We then calculated alpha diversity, a measure of within-sample microbial diversity within each body site (that is, richness within a sample), and found it to be correlated with the first coordinate of host genetic variation data in the anterior nares (Fig. 1a, R^2^ = 0.207, *P* = 0.039) and the right retroauricular crease (Additional file 1: Figure S11, R^2^ = 0.218, *P* = 0.01). In addition, we found correlations in several additional coordinates; for example, the third principal component (PC) of host genetic variation is correlated with alpha diversity in the supragingival plaque, the throat, and the tongue dorsum (Additional file 1: Figure S11). Reduced alpha diversity has been previously linked to different health conditions (for example, inflammatory bowel disease [7], type 2 diabetes [11], and obesity [35]), and our results suggest a possible role for host genetics in controlling the alpha diversity. Next, we looked for correlations of host genetics with the overall composition of the microbiome. We found correlations between the first host genetic principal coordinate and microbiome PCs in the stool and palatine tonsils (Fig. 1b and Additional file 1: Figure S12). We also found correlations at a number of other body sites, although most were not statistically significant after multiple test correction (Additional file 1: Figures S12-S17). Nevertheless, taken together, these correlations suggest a potential relationship between host genetics and microbiome composition.

This dataset also allows us to compare between-individual differences in the microbiome and host genetic variation. We correlated microbial beta diversity (that is, between-sample diversity) at each body site with genome-wide identity-by-state, a statistic estimating similarity in genome sequence between pairs of individuals. We found that identity-by-state is significantly negatively correlated with beta diversity in 10 of the 15 body sites (Additional file 1: Figure S13), including in the stool (Fig. 1c, R^2^ = 0.19, *P* <10^15^), anterior nares, hard palate, palatine tonsils, saliva, supragingival plaque, throat, and tongue dorsum (*P* <0.01 in each of the 10 body sites). These results indicate that the similarity in genome sequence is positively correlated with microbiome similarity, supporting a relationship between host genetic variation and the microbiome at a large scale. However, this pattern may be partly driven by population stratification, or non-genetic environmental factors that are correlated with genetic ancestry. For example, previous studies have found differences in the gut microbiome between human populations [36, 37], so geographic stratification could drive a biologically non-causal correlation between genetic ancestry and local diet, and thus with gut microbial composition.

### Host genes and pathways correlated with microbiome composition

In an effort to control for population structure, in addition to other non-genetic factors that may be driving spurious correlations, we analyzed the data using a linear mixed model. The additive effects model included as covariates possible confounders, such as gender, sample collection location, sequencing center, and the first five coordinates from the MDS analysis of the host genotypic data. By including these covariates we are attempting to correct for effects of individual ancestry and extrinsic factors on the microbiome. We note that there are additional potential confounding factors that we could not account for in our model; for example, physical interaction between individuals, which has been shown to affect microbiome composition in primates [20], is not included, as these data were not collected by the HMP. We ran this model genome-wide, correlating host genetic variation in each SNP with the first five PCs of the microbiome in each of the 15 body sites. In addition to controlling for confounders, this genome-wide approach also allows us to identify specific loci in the host genome that are correlated with the microbiome, and understand their likely functional effect in the host. We recognize at the outset that our sample size is an order of magnitude smaller than most genome-wide association studies (GWAS), precluding us from being able to perform a standard test of association between microbiome composition and each SNP. Therefore, instead, we used a pathway-based analysis, whereby we aggregated SNPs into pathways in order to learn about the biological functions and processes that underlie interactions between host genome and the microbiome. We note that this is a common analysis approach for genome-wide association data, driven by the rationale that complex traits are controlled by multiple genetic effects, which could originate in different genes, but are likely to aggregate in the same biological pathway or function. The approach is aiming to identify these functions by looking for enrichments of biological functional categories among a set of associated genetic loci. Specifically, we first aggregated SNPs that were correlated with at least one microbiome PC at an arbitrary nominal cutoff of *P* ≤10^−6^ (using several other *P* value thresholds did not change the results; see Additional file 2: Tables S1 and S2). We then identified overlapping or nearby genes, and used these gene sets to perform a functional enrichment analysis.

Using this approach, we found the most significant enrichment with genes involved in pathway Leptin Signaling in Obesity (*P* = 2.29 × 10^−7^, Additional file 2: Table S1). Leptin is a hormone whose structure places it in the cytokine superfamily. It has been linked to the microbiome in several recent studies, mainly using leptin-deficient *ob/ob* mice [13, 38]. Leptin has several important roles in immunity, including activation of monocytes, neutrophils, and macrophages, and modulation of inflammation [39]. Leptin may also impact the microbiome indirectly in its role as a hormone, whereby it regulates appetite and body weight, affects basal metabolism, and regulates insulin secretion, among other functions [39]. The enrichment identified here is driven by significant correlations of host genetic variation with microbiome PCs in the nose, oral cavity, and skin (see Additional file 2: Table S1)*.* Studies have shown that the leptin is expressed and has a functional role in the mouth [40]. Leptin and leptin receptor are expressed in the skin [41], and may have a functional role in wound healing and psoriasis [42, 43]. Moreover, leptin is expressed in nasal polyps, and may affect the expression of mucin genes in polyp epithelial cells [44]. Nevertheless, the role of leptin in interactions with microbial flora in these body sites is still not well understood.

In addition to leptin signaling, several other immunity-related pathways are enriched among microbiome-correlated host genes, such as Melatonin Signaling, JAK/Stat Signaling, Chemokine Signaling, CXCR4 Signaling, and Role of Pattern Recognition Receptors in Recognition of Bacteria and Viruses (Additional file 2: Tables S1 and S2). To further investigate the role of host genetic variation in immunity-related genes on the microbiome, we used the InnateDB database, and identified additional enriched pathways, including Interleukin-12-Mediated Signaling Pathway, GABA_A_ Receptor Activation, Inositol Phosphate Metabolism, IL2, CXCR4-Mediated Signaling Events, and GnRH Signaling Pathway (Additional file 2: Tables S3 and S4). In addition, we found enrichment of genes in the REACTOME pathway Sulfide Oxidation to Sulfate, suggesting a potential role for host genetic variation in genes determining sulfate abundance in controlling microbial composition. We also found enrichment in the KEGG pathway Primary Bile Acid Biosynthesis. Recent studies have shown that the microbiome can modulate bile acid metabolism [45], and our results support a possible role for host genetic variation in bile acid metabolic pathways in interacting with the microbiota.

**Fig. 2:**
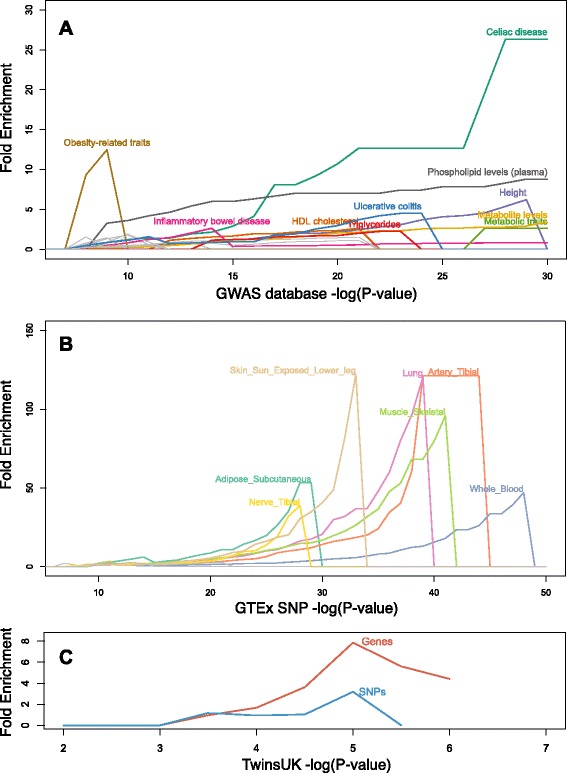
Complex disease and functional SNPs are enriched among microbiome-correlated host genetic variation. **a** Enrichment of genes correlated with microbiome composition (*y*-axis) compared to all other genes that are significantly associated with a complex disease using a given *P* value threshold (*x*-axis). Each colored line represents a different complex disease with an enrichment of at least three-fold. **b** Enrichment of SNPs correlated with microbiome composition (*y*-axis) compared to all other SNPs that have been identified as eQTLs in the GTEx data using a given *P* value threshold (*x*-axis). Each colored line represents a different tissue type analyzed by GTEx. **c** Enrichment of SNPs (blue) and genes (red) correlated with microbiome composition in this study (*y*-axis) among SNPs and genes correlated with microbiome composition in the TwinsUK dataset using a given *P* value threshold (*x*-axis)

Next, we examined correlations between microbiome composition and host genetic loci that had been found to be associated with complex disease. For that purpose, we used the GWAS catalog [46], and looked for enrichment of genes found to be associated with specific complex disease. For each disease in the catalog, we plotted the overlap between the genes associated with the disease and the genes found in our study to be associated to microbiome composition. Plotting this overlap over a range of *P* value cutoffs for each GWAS dataset, we detected enrichments in a number of diseases (Fig. 2a). We found enrichments in genes associated with several complex diseases for which a role for the microbiome has been shown, such as ulcerative colitis [47], inflammatory bowel disease [48], obesity-related traits [7], and HDL cholesterol and triglycerides. In addition, we found enrichment of genes associated with metabolite levels and metabolic traits, for which an interaction with the microbiome has been observed [35].

We used a similar approach to identify enrichment of SNPs annotated as expression quantitative trait loci (eQTLs) among the sites we found to be correlated with microbiome composition (Fig. 2b). We found an enrichment of eQTLs in several tissues that were identified in the GTEx project [49]. This result indicates that the loci we identified in our analysis as correlated with microbiome composition are likely to have a functional role in regulating gene expression. Lastly, we sought to validate our results using an independent cohort. We followed a similar approach to identify correlations between GI tract microbiome PCs and host genetic variation in 984 individuals from the TwinsUK project cohort [14, 50]. We find an enrichment of SNPs correlated with microbiome composition in both studies (Fig. 2c; *P* = 0.028 using Fisher’s exact test for significant overlap between the two sets of SNPs). When considering genes located nearby correlated SNPs, the enrichment becomes more prominent; possibly indicating that different SNPs may control similar microbiome-linked genes and pathways.

### Host genetic variation correlated with bacterial taxa

In addition to identifying interactions with the overall structure of the microbiome, we were interested in finding correlations between host genetic variation and specific bacterial taxa. To do so, we tested for correlation between genetic variation and relative abundances of bacteria derived from the HMP 16S rRNA gene sequences. Abundance data from HMP OTUs were parsed, extensively filtered, normalized, and taxonomically collapsed, to achieve a single representation for each taxon at the genus level or above (see Additional file 1: Figures S14-S19 and Additional file 3). After filtering inter-correlated taxa, our final dataset included 615 microbiome abundance traits in 15 body sites. In an effort to reduce the number of statistical tests, we included in the analysis only host SNPs located within protein-coding sequences.

**Fig. 3:**
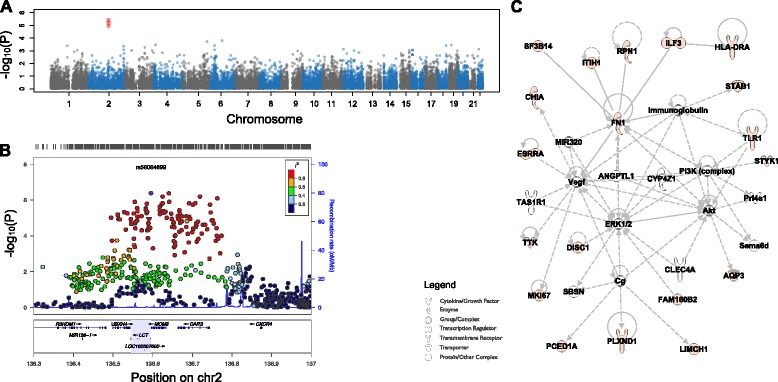
Correlation between coding genetic variation and bacterial abundance. **a** Manhattan plot illustrating the *P* values (y-axis, −log scale) for correlation of each tested coding SNP (shown as circles) by its genomic location (x-axis) with the abundance of *Bifidobacterium* in the gut. SNP colors alternate by chromosome, with red dots representing SNPs with *P* values that surpass genome-wide significance after FDR correction. **b** A close-up of the region of correlation within LCT. Genomic positions on chromosome 2 are on the *x*-axis, and the *P* values are on the *y*-axis (−log scale). Each dot represents a SNP tested using our model, and the color represents the linkage disequilibrium (*r* ^*2*^) between each dot and the top SNP, colored purple and indicated by its dbSNP rsID (inset legend indicates the spectrum of colors and matching *r* ^*2*^ values). Blue lines represent recombination rate calculated from the European samples in the 1000 Genomes Project. Gene regions are shown underneath, with LCT highlighted. **c** An interaction network generated using IPA showing pathways that are enriched among genes that harbor SNPs correlated with abundance of bacterial taxa (in orange). Lines represent known interactions between genes, and shapes represent types of proteins (see legend at the bottom left)

Using this approach, we found 83 associations between genetic variation in host coding sequence and abundance of specific microbial taxa (genome-wide false discovery rate Q-value <0.1). These 83 associations are described in Additional file 2: Table S5. Among these, we find several key host genes related to immunity, such as *HLA-DRA* (*P* = 3.72 × 10^−6^) and *TLR1* (*P* = 5.04 × 10^−6^), which we found to be correlated with abundance of *Selenomonas* in the throat and Lautropia in the tongue dorsum, respectively. Another interesting correlation was found between host genetic variation in SNPs in the *LCT* gene and the abundance of *Bifidobacterium* in the GI tract (*P* = 1.16 × 10^−5^, Fig. 3a, b). *LCT* encodes the lactase enzyme, which is expressed in the GI tract and acts to hydrolyze lactose, the sugar found in dairy products. Intriguingly, *Bifidobacterium* can metabolize lactose, and reports show that some strains prefer lactose to glucose [51]. Since genetic variants in and around *LCT* are directly linked to lactase persistence [52], it is likely that the variants we observed dictate an individual’s consumption of milk products, which in turn may regulate the abundance of *Bifidobacterium* in the GI tract. Although the data do not provide sufficient resolution to discriminate the *Bifidobacterium* species that drives this association, further analytical and experimental approaches may shed light on this result.

Using pathway enrichment approaches described above, we found that genes linked to abundance of bacterial taxa are over-represented with relevant diseases (Additional file 2: Table S6), including transendothelial migration of lymphocytes, meningitis, and several cancer categories, including gastrointestinal adenocarcinoma, growth of mammary tumor, head and neck tumor, and thyroid cancer. To further visualize the interactions between these genes, we used the Ingenuity Pathway Analysis knowledgebase, which holds curated information on molecular pathways and protein interactions, and identified several networks significantly enriched with genes correlated with bacterial taxa abundances (Additional file 2: Table S7). Figure 3c displays the highest-scoring network, containing genes involved with cellular movement, hematological system development and function, and immune cell trafficking.

**Fig. 4:**
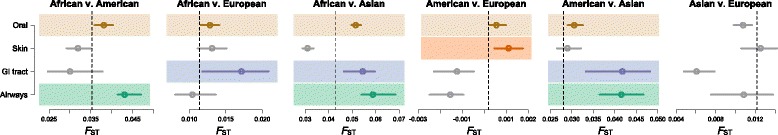
SNPs correlated with microbiome composition have high F_ST_ values between human populations. Each panel represents a comparison of a pair of human populations indicated in the title. Shown is the *F*_ST_ median + 95 % CI (x-axis, calculated using bootstrapping) in SNPs where genetic variation is correlated with microbial taxa at *P* <10^−4^, separated by the body site (y-axis). Vertical dashed line represents the genome-wide median F_ST_. Color highlight was used in cases where *F*_*ST*_ in microbiome-correlated sites was significantly higher than the genome-wide value (FDR Q <0.05; using a permutation test of the median)

Lastly, we investigated the evolutionary pressures acting on the SNPs we found to be correlated with microbiome composition. To do so, we used *F*_ST_, a measure of allele frequency differentiation between human populations, calculated from the 1000 Genomes Project data (see Materials and Methods) [34]. Comparing *F*_ST_ between four human populations (African, American, Asian, and European), we found that SNPs that were linked to microbial communities in our study have higher *F*_ST_ values compared to the rest of the genome (Fig. 4; FDR Q <0.05 for the highlighted comparisons using a permutation test on the medians; see Additional file 3). Interestingly, we found that in some body sites, the microbiome is linked to genes with higher *F*_ST_ values across most population comparisons; for example, the oral cavity microbiome is linked to higher *F*_ST_ in all pairwise comparisons among populations, except Asian vs. European. In addition, specific population pairs seem to be enriched with higher *F*_ST_ across body sites; for example, both the African vs. Asian and the American vs. Asian comparisons show high *F*_ST_ values in the genes that interact with microbial communities in three of the four body sites (oral cavity, GI tract, and airways). Overall, 12 of the 24 comparisons yielded significantly high *F*_ST_ compared to the genome-wide average, while six comparisons yielded significantly lower values.

These results suggest that host genetic variation that is linked to microbial variation is enriched with sites that evolve under differential selection pressures across human populations. This is consistent with the notion of local adaptations to population-specific microbiomes, possibly controlled by environmental conditions for each population. Given that genes that we found to be linked to microbiome composition are enriched with immunity-related genes and pathways, this result may not be surprising; indeed, genetic variation in immune genes has long been associated with higher rated of positive selection in human populations [53]. However, these selective pressures were hypothesized to be mainly a result of interaction with pathogens. Our results indicate that selection pressures on immunity genes and pathways may also be due to interaction with commensal microbial communities that accompany changing environments. Another potential explanation for this pattern is that past selection pressures against pathogens have driven changes in immunity genes that affect the commensal microbiome as a byproduct. Although distinguishing between these hypotheses is not possible using currently available data, the end result – commensal microbial traits affected by past selection events on host genes – is an exciting finding that we hope would be explored further in the future.

## Conclusions

We describe an analysis of host genetic variation data mined from the metagenomic shotgun sequencing performed by the Human Microbiome Project. The ability to mine host genetic material from metagenomic shotgun sequence data has recently raised several privacy concerns [54]. We note that in the current study, informed consent for sequencing of host DNA was given by the participants, although this is not a common procedure for metagenomics studies. We show here that it is possible to reconstruct complete host genomes using metagenomic sequence data, which is potentially identifiable. However, this was possible due to the unique study design of the HMP, whereby multiple body sites from each individual were sequenced at a high depth, allowing us to pool data across body sites and reach a 10x mean coverage per host genome. Common metagenomic shotgun sequencing studies, which usually include an order of magnitude less sequence data, are unlikely to enable such an analysis. Moreover, the majority of studies sequence stool samples, which include many fewer host-derived reads. Nevertheless, we anticipate that future shotgun metagenomics sequencing studies would consider these potential privacy concerns.

The analysis described in this paper focused on the taxonomic structure of the microbiome. However, it would be interesting to incorporate the functional composition of the microbiome when considering associations with host genetic variation. Indeed, several studies have highlighted the importance of shotgun metagenomics for uncovering the genic composition and metabolic capacity of the microbiome [1, 48]. A similar analysis would be critical to uncover functional interactions that could not be detected by looking at community and taxonomic composition. In addition, there are several environmental factors that could influence the microbiome, such as diet, which were not included in our analysis. We expect that the inclusion of such potential confounders in future studies would help to further disentangle the effects of environment and host genetic variation on the microbiome.

Our analysis has shown that host genetic variation in immunity-related pathways is correlated with microbiome composition. These results are consistent with recent reports of host immunity involvement in modulating microbiome structure, for example through production of antimicrobial compounds [55] or inflammation [56]. Additionally, many recent studies have shown that a mice with a knocked-out immune gene display dramatic changes in their microbiota [57–60]. Moreover, genetic variation in immune genes in the mouse was found to be correlated with the composition of the microbiome [61]. In addition, our results show that the host variants and genes that are correlated with the structure of the microbiome are enriched in genes associated with complex disease that have been linked to the microbiome. This result is not surprising, considering that recent studies in the mouse have shown that microbiome QTLs overlap complex disease-linked genes [28, 29]. Taken together, these findings motivate the need for larger association studies to characterize host genetic variation linked to the microbiome in the context of various health conditions, environmental effects, and genetic backgrounds. Moreover, functional studies, for example using cells or animal models, would be crucial for elucidating the causal mechanisms whereby human genetic variation impacts the microbiome.

## Materials and methods

A full and detailed description of the Methods is available in the Additional file 3 document.

### Ethical statement

Recruitment protocols were approved by Institutional Review Boards at each HMP clinical site, and written informed consent was obtained from all study participants for data sharing through dbGap. All study participants have consented for the sequencing of their own genetic material [33]. Specifically, the HMP human subjects study was reviewed by the Institutional Review Boards (IRBs) at each sampling site: the BCM (IRB protocols H-22895 (IRB no. 00001021) and H-22035 (IRB no. 00002649)); Washington University School of Medicine (IRB protocol HMP-07-001 (IRB no. 201105198)); and St Louis University (IRB no. 15778). The study was also reviewed by the J. Craig Venter Institute under IRB protocol 2008–084 (IRB no. 00003721), and at the Broad Institute of MIT and Harvard the study was determined to be exempt from IRB review.

### Host read data acquisition, filtering, and alignment

The processing of the raw data files through the genotyping step was performed on the compute cluster at the Broad Institute. We downloaded 1,553 raw Illumina read files (total of 8 TB) in SRA format, representing samples from 98 individuals (HMP subjects), from the dbGaP database. The files were decrypted, and converted to FASTQ format using NCBI’s SRA toolkit (version 1.0.0-b10) with default parameters. A total of 152 files that failed the standard Illumina quality checks were excluded from the downstream analysis. The reads from the remaining 1,401 files were aligned to the human genome (build hg19) using BWA v0.5.7 [62] with default settings for the alignment, except for the ‘bwa sampe’ step, where the option ‘-a 2000’ was used to change the maximum insert size from default 500 to 2,000. Out of the 79,877,504,468 post-filter reads, 35,828,514,379 were mapped to the human genome. The 1,401 BAM files were reorganized by merging reads from different samples from the same subject into subject BAM files using samtools [63]. The merging failed for one individual (due to corruption of the original sample BAM files), and for four others the merged BAM files contained only reads from stools samples very little human DNA present. These five subjects were excluded, leaving 93 individuals. The average number of mapped reads per individual was 365 million.

### Genotype calling, filtering, and QC

Variants (SNPs and short indels) were called from all 93 cleaned and re-aligned BAM files using the GATK’s UnifiedGenotyper function with standard emission confidence parameter set to 3.0 (−stand_emit_conf 3.0). This value, much lower than the GATK default, was used in order to provide an exhaustive list of possible variants for subsequent filtering. The coverage for each individual was down-sampled to 200 (that is, the option –dcov 200 was used). Other options of UnifiedGenotyper were kept at their default values. The calculation was parallelized over genomic coordinates by splitting the genome into 80,000 bp intervals and running UnifiedGenotyper for each of these intervals on a separate processor of the compute cluster. After excluding contigs that did not map to a known chromosome, this unfiltered, low-pass genotype set included 19,377,382 SNPs and 3,519,487 short InDels. In order to filter the genotype calls and keep only high-quality variants, we used GATK and applied several hard filters that are recommended for low-coverage whole-genome data [64]. Specifically, we excluded SNPs with low mapping quality, SNPs with a strand bias, and SNPs that are otherwise of low quality. In addition, we masked out SNPs that are near InDels using a window size of 10. Lastly, we excluded any SNPs for which there is missing information and a clear filter decision could not be made.

Next, we performed variant score recalibration on the SNPs that have passed the above filters using the GATK VariantRecalibrator. As input to train the model, we used three input SNP sets: (1) HapMap3.3 SNPs; (2) dbSNP build 132 SNPs; and (3) 1000 Genomes Project SNPs from Omni 2.5 chip. After applying the recalibration using the GATK ApplyRecalibration command and excluding variants that did not pass the various filters, we were left with 13,190,940 SNPs across the 93 individuals. Of this set, 7,229,492 SNPs (60.3 %) were also found in dbSNP. As quality control, we plotted the number of sites filtered out by each filter or combination of filters, as well as the Ti/Tv ratio for each filter combination (Additional file 1: Figure S5). The sites that passed our filtering criteria have the highest Ti/Tv ratio (mean 2.1), which is close to the expected value observed in many sequencing projects, including the 1000 Genomes Project pilot data (genomic average Ti/Tv of 1.96) [65]. When we consider the frequency spectrum of alleles in our sample (Additional file 1: Figure S6), we see an enrichment of low-frequency variants, as consistent with many recent population-scale sequencing studies [66]. We see a similar distribution when we consider allele sharing across individuals (Additional file 1: Figures S8 and S9), with most alleles appearing in only one individual. Since alleles at lower frequencies are less informative for association analysis, we excluded from downstream analysis SNPs that are at frequency of less than 5 % in our sample, leaving us a set of 5,536,004 SNPs. Of this set, 5,108,016 SNPs are also found in dbSNP (92.3 %). We further filtered this set keeping only SNPs with minor allele frequency above 10 %, SNP with *P* value >10^−3^ for Hardy-Weinberg equilibrium, autosomal SNPs, and SNPs with less than 50 % missing information. The final set included 4,205,323 SNPs that set that passed these QC thresholds and were used in the analysis. Pairwise identity-by-state (IBS) distances between individuals were calculated from the filtered SNP data using PLINK [67, 68]. We performed metric multidimensional scaling analysis (MDS) on the pairwise IBS distance matrix using PLINK.

### Correlation and enrichment analysis

We used the first five principal coordinates (PCs) of the microbiome 16S data in each of the 15 body sites as quantitative traits, which we correlated against genetic variation in the host. Prior to running this analysis we normalized the PC values using the Box-Cox transformation with the formula

$$ {y}^{\left(\lambda \right)}=\left({y}^{\lambda}\hbox{--}\;1\right)/\lambda $$

Where λ was calculated using the function box.cox.powers in R (in the package ‘car’). Correlation analysis of normalized trait values was performed in PLINK v1.07 [67], and included the following covariates: (1) Individual sex (binary variable); (2) Individual age; (3) Site where microbiome data were collected; (4) Center where sequencing was performed (this was coded as binary variables representing the four collection centers: BCM (Baylor College of Medicine), BI (Broad Institute), JCVI (J. Craig Venter Institute), and WUGC (Washington University Genome Center); (5) The total number of sequences for each individual in the metagenomic sequencing data; and (6) The positions on the first five dimensions in the MDS analysis of the genotype data. In addition to the microbiome PCs, we also ran a similar correlation analysis for a set of microbiome taxa, following a comprehensive filtering of the 16S OTU data as described in Additional file 3. To reduce the multiple test burden, this analysis was performed on a set of protein-coding host SNPs, which were identified after annotation of the SNP data using ANNOVAR [69], and included 33,814 protein-coding SNPs.

We considered SNPs correlated with microbiome PCs with *P* value ≤10^−6^, and identified genes that overlap or are located ≤50 kb from these SNPs, using data for all known human genes taken from the refGene table (hg19 genome build). The identified genes were used as input to functional enrichment analysis, performed using Ingenuity Pathway Analysis (IPA; August 2012 software release), a program that uses Ingenuity’s high-quality knowledge base, which includes curated information on genes, pathways, and interactions (see [70]). IPA generates a *P* value using a Fisher’s exact test comparing the expected and observed genes in a given pathway. The most enriched canonical pathways are listed in Additional file 2: Table S1. To identify the bacterial taxa driving these enrichments, we calculated correlations between each OTU and the PCs in each body site. The most highly correlated OTU for each PC where correlation with host genetics was found is listed in Additional file 2: Table S1. We also used the InnateDB database [71] to identify enrichment of specific gene ontology (GO; [72, 73]) categories (Additional file 2: Table S3) and additional pathway databases (Additional file 2: Table S4), including KEGG [74] [75] and Reactome [76]. To make sure the specific cutoff values chosen in this analysis do not affect the enrichment result, we repeated this analysis with varying *P* value and gene distance cutoffs (see Additional file 2: Table S2). Specifically, we used two *P* value cutoffs (*P* ≤10^−6^ and *P* ≤5×10^−7^) and three gene distance cutoffs (*D* ≤50 k, *D* ≤20 k, and *D* ≤5 k), and examined the enrichment *P* value and rank of pathways of interest (Additional file 2: Table S2).

The data from the GWAS Catalog [46] and the GTEx consortium [49] presented in Fig. 2 were downloaded from [77] in June 2013, and the GTEx portal [78] on October 2013, respectively. The enrichment plots shown in Fig. 2 were calculated as follows: given a dataset (for example, GWAS catalog genes involved in obesity-related traits), and given a *P* value cutoff (*P*_*i*_, shown on the *x*-axis of the figure), we identified the set of genes or SNPs for which *P* ≤*P*_*i*_. Next, we calculated the overlap between *G*_*i*_ and the genes or SNPs identified to be correlated with the microbiome in the current paper. The fold enrichment (*y*-axis) for *P*_*i*_ is the number observed compared to expected overlapping genes or SNPs, where the expected number is the overlap among genes or SNPs not in *G*_*i*_.

To identify enrichment in an independent cohort, we used data from the TwinsUK Project, which included both stool microbiome 16S data, as well as host genetic data assessed by SNP genotyping, from 984 adults [14]. OTU tables and PCs were generated using the QIIME pipeline as described above [79–82]. Host SNP genotyping data were fully imputed using IMPUTE version 2[83], and quality checked as previously described [50]. SNPs were removed if they had a minor allele frequency below 5 %, a genotyping rate below 95 % or extreme deviation from HWE (*P* <0.001). Deviation from HWE was determined using the genotypes from only a single twin from each twin pair. Only imputed SNPs with an imputation accuracy score (IMPUTE *INFO* field) greater than 0.9 were included in the analysis. The final number of SNPs used for the association analysis was 1,310,141. To test for correlation between host SNPs and fecal microbiome PCs, we used the score test implemented in the software Merlin [84] to account for the relatedness of the individuals (option –fastassoc). The recombination rates from HapMap II release 22 were used as the genetic map input to Merlin. Model covariates included the number of sequences per sample, sample batch, sequencing run, the person that extracted the DNA, the gender, the age, and the first three PCs of the MDS. After quality filtering of traits and genotypes, 170 MZ twin pairs, 241 DZ twin pairs, and 162 unrelated individuals were included in the association analysis. For the analysis shown in Fig. 2c, we used correlation *P* values for SNPs and nearby genes, and calculated fold-enrichment for several *P* values as described above.

### *F*_ST_ analysis

We used *F*_ST_ data downloaded from the database of recent positive selection across human populations [85] via [86] in March 2014. We compared *F*_ST_ values in SNPs that were correlated with microbiome PCs with *P* <10^−4^ in each of the four body sites and the rest of the SNPs in our sample. To compare two sets of *F*_ST_ values we used a permutation test on the medians as follows: we randomly split the data into two groups the same size of the two original groups, and calculated the difference in medians between the two groups. This process was repeated 10,000 times, and the *P* value was defined as the proportion of permutations in which difference in medians was greater that the real difference between the two original groups. Figure 4 shows all the comparisons made and highlights in color cases where the calculated *P* value was smaller than 10^−3^. The error bars in the figure are 95 % confidence intervals that were calculated using bootstrapping as follows: for a given set of *F*_ST_ values, we subsampled with replacement a sample of the same size, and calculated the median of the sample. This was repeated 10,000 times, with the median recorded in each iteration. The 95 % CI was defined as the range between the 2.5 and 97.5 percentiles of all subsample medians.

### Data deposition

16S rRNA gene sequence data and OTU tables are available on the HMP DACC website [87]. Host genetic data are deposited in dbGaP under project number phs000228.

## Additional files

Additional file 1: **This is a PDF document containing Supplementary Figures S1 through S19.** (PDF 12238 kb)

Additional file 2: **This is a PDF document containing Supplementary Tables S1 through S7.** (PDF 622 kb)

Additional file 3: **This is a PDF document containing detailed supplementary materials and methods.** (PDF 300 kb)

## Abbreviations

eQTL: Expression quantitative trait locus
GWAS: Genome-wide association study
HMP: Human microbiome project
IBD: Inflammatory bowel disease
MDS: Multidimensional scaling
OTU: Operational taxonomic unit
PC: Principal component
QTL: Quantitative trait locus
SNP: Single nucleotide polymorphisms
