# Supplementary material for: Host genetic variation impacts microbiome composition across human body sites
Source: Genome Biol. 2015 Sep 15;16(1):191. doi: 10.1186/s13059-015-0759-1 (PMC4570153; doi:10.1186/s13059-015-0759-1)
Supplement: Supplementary file 1 — This is a PDF document containing Supplementary Figures S1 through S19. (PDF 12238 kb) [file 13059_2015_759_MOESM1_ESM.pdf]

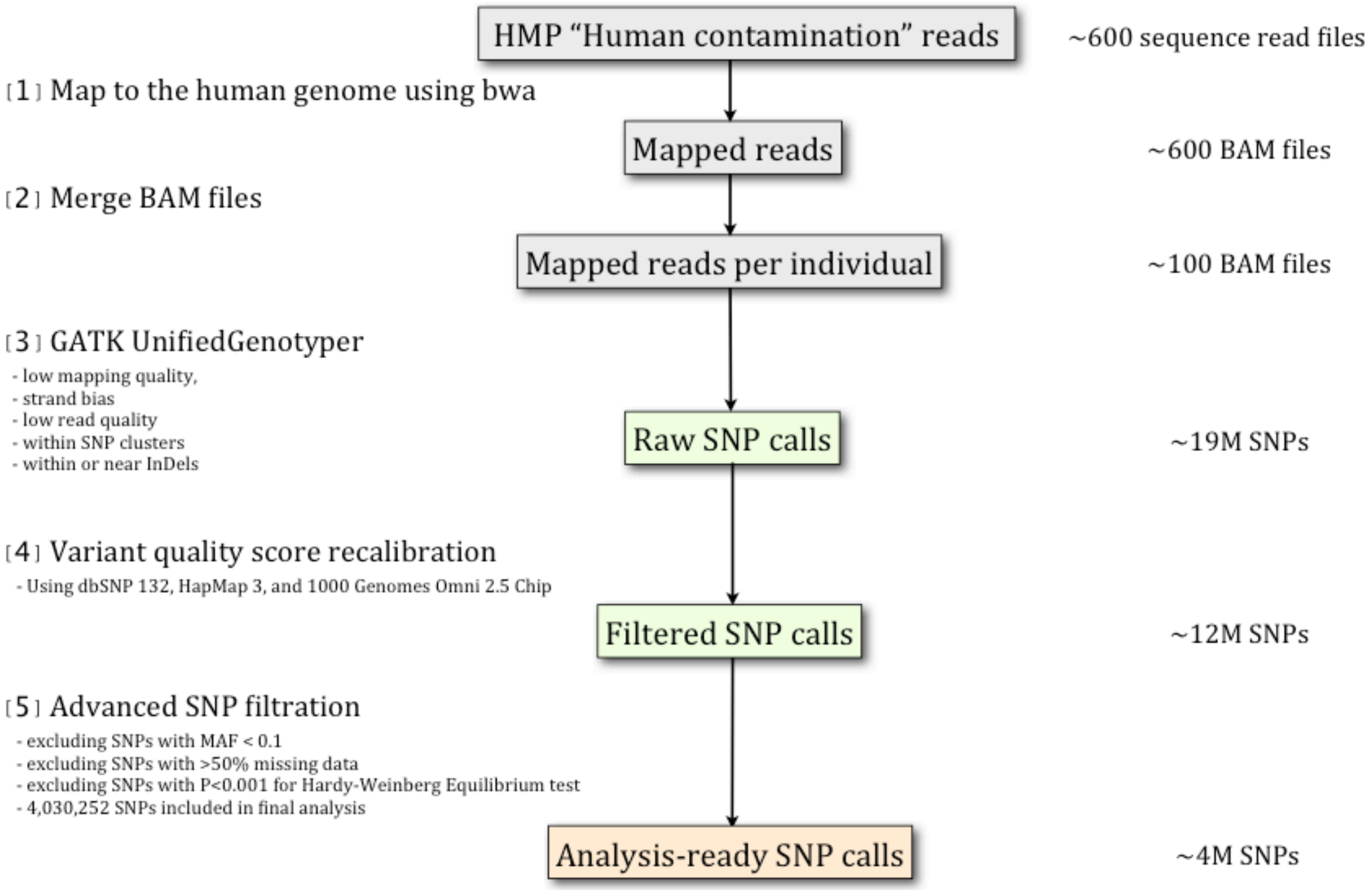

Additional file 1

Figure S1. An outline of the pipeline used to generate high-quality host genotype data from the microbiome shotgun sequencing data.

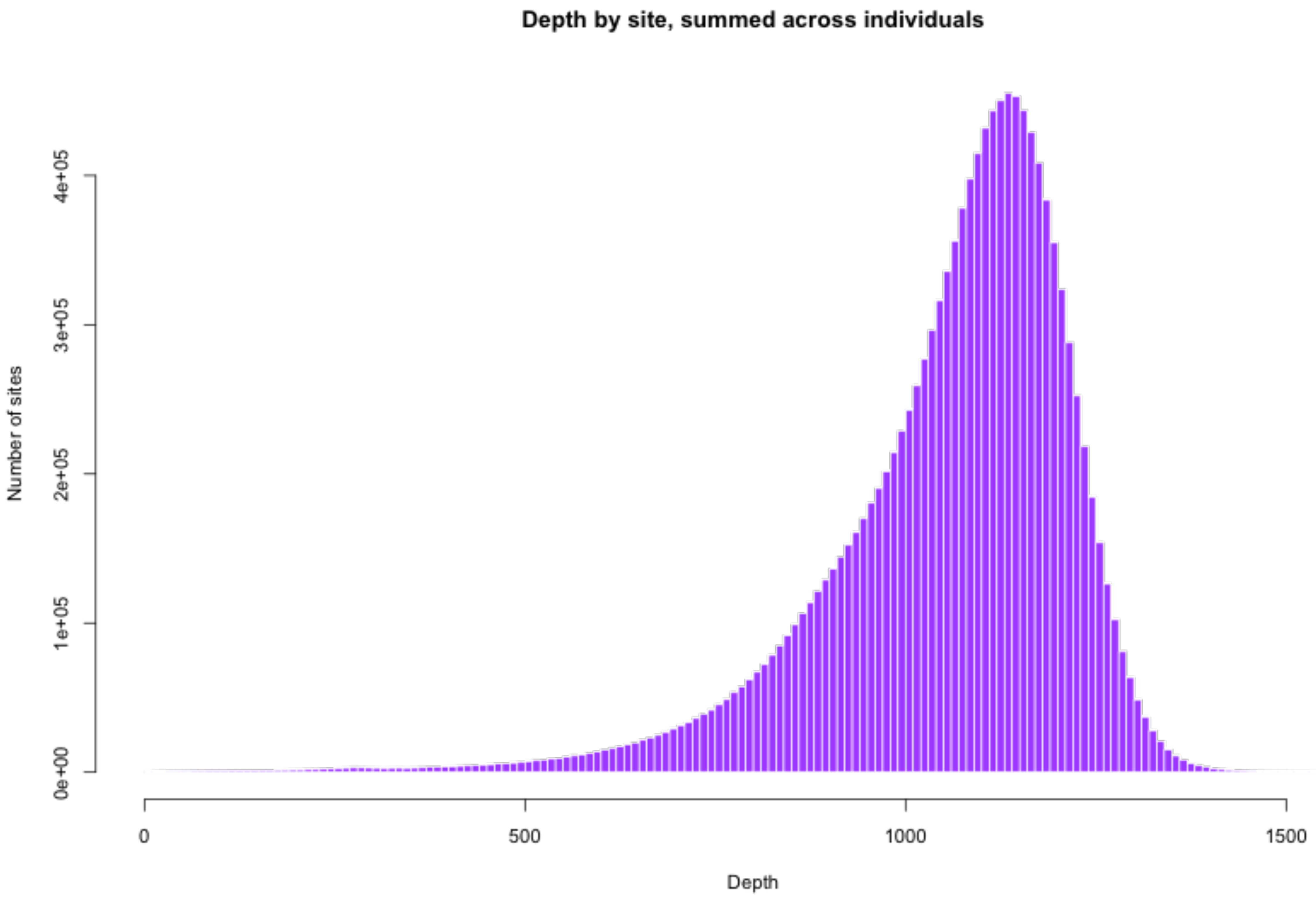

Figure S2. Distribution of per-base depth of coverage, summed across all individuals in the study.

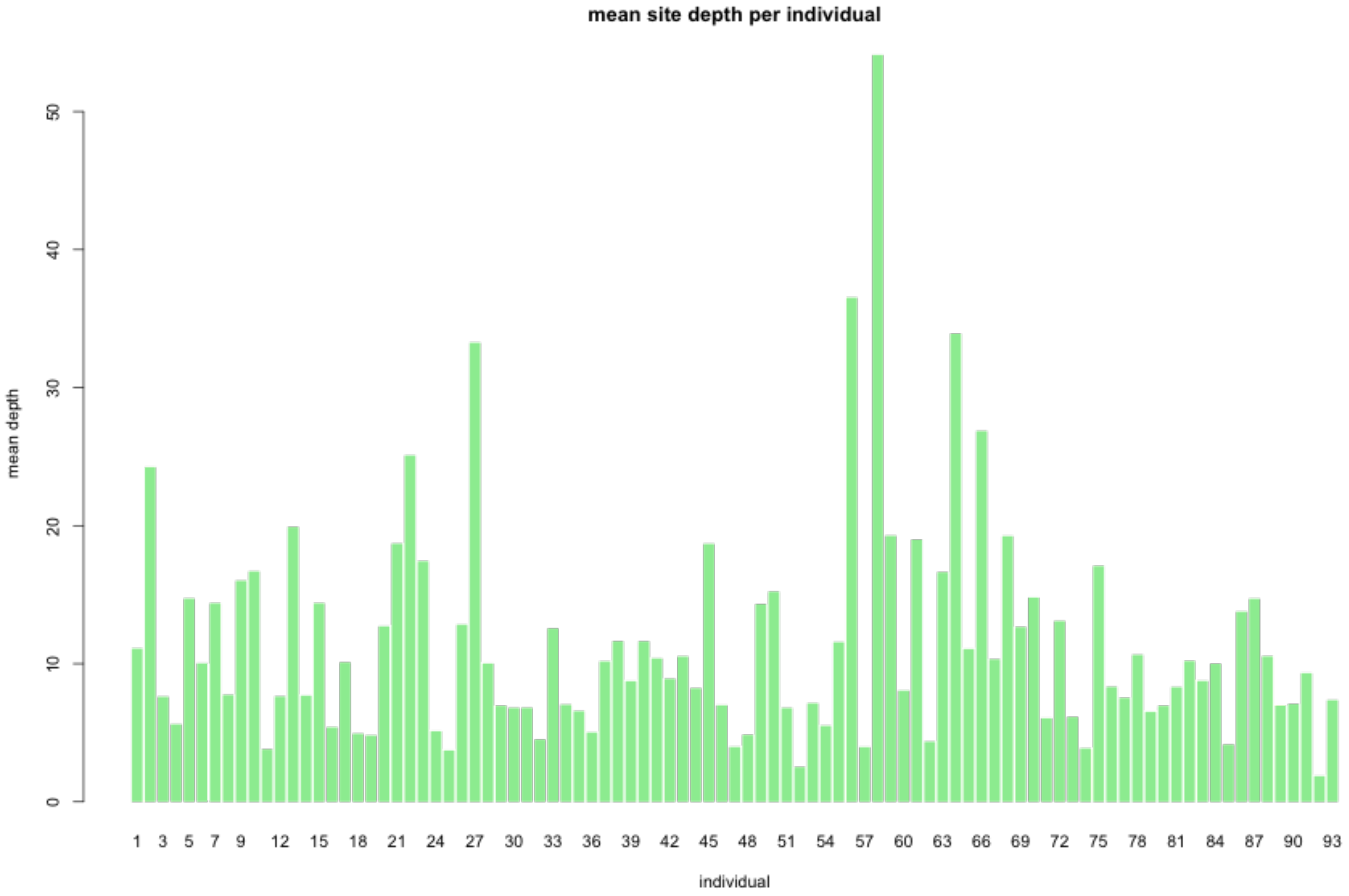

Figure S3. The mean depth of coverage (y-axis) for each of the 93 individuals in our study (x-axis).

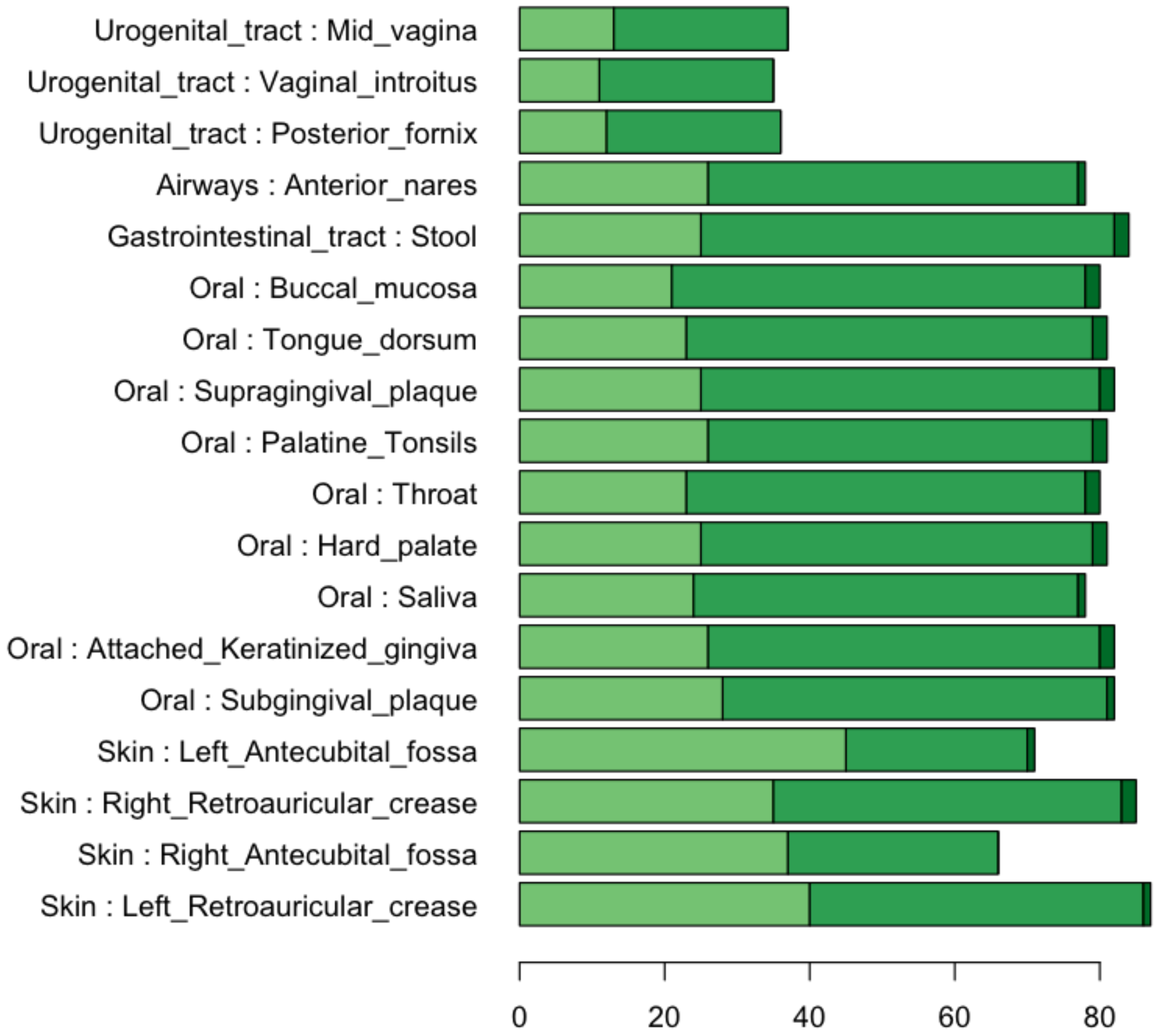

Figure S4. Number of individuals for which we had genotype and microbiome data with one (light green), two (green), and three (dark green) microbiome samples, plotted for each body subsite.

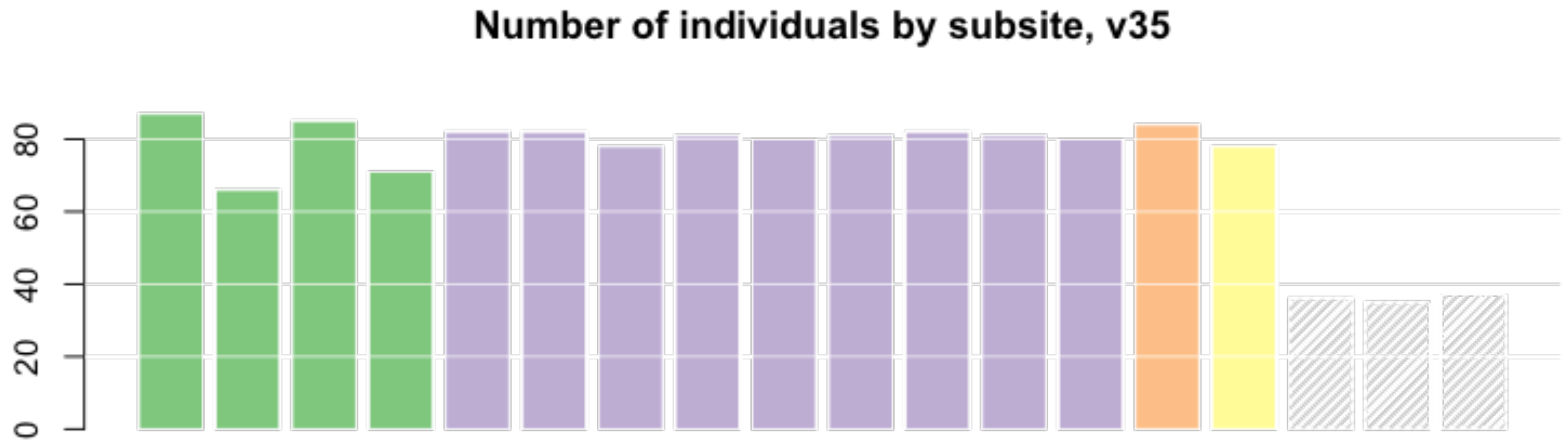

Figure S5. Number of individuals for which we had genotype data for each body subsite and used in the association analysis; green: skin; purple: oral; orange: GI tract; yellow: airways; grey: urogenital tract (not used in association analysis).

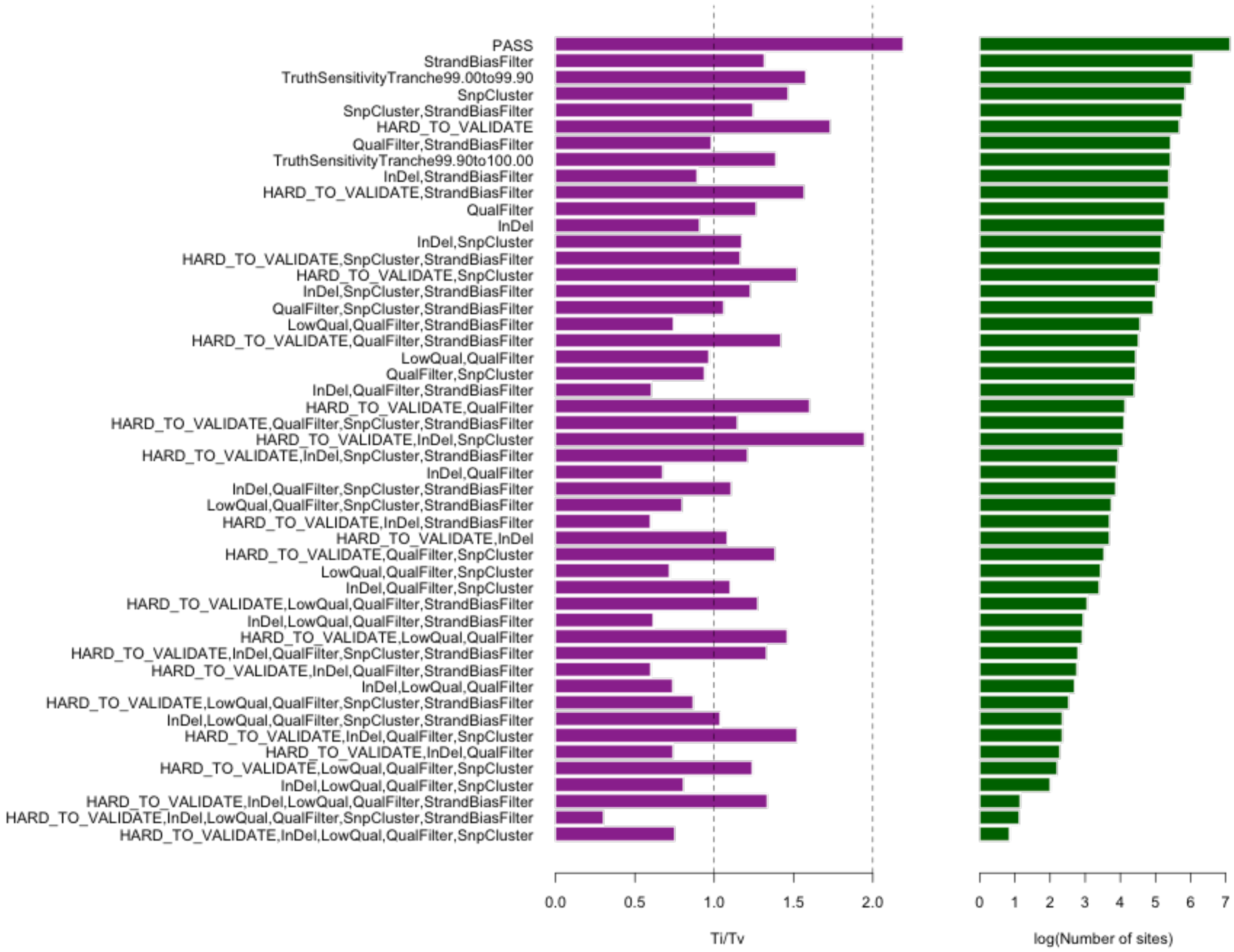

Figure S6. For each combination of SNP filters used on the raw genotype data (y-axis), we plot the Ti/Tv (purple) and number of sites (green, log-scale) filtered out. The first category (marked 'PASS') represents the sites that have passed all filtering criteria and are included in the final set.

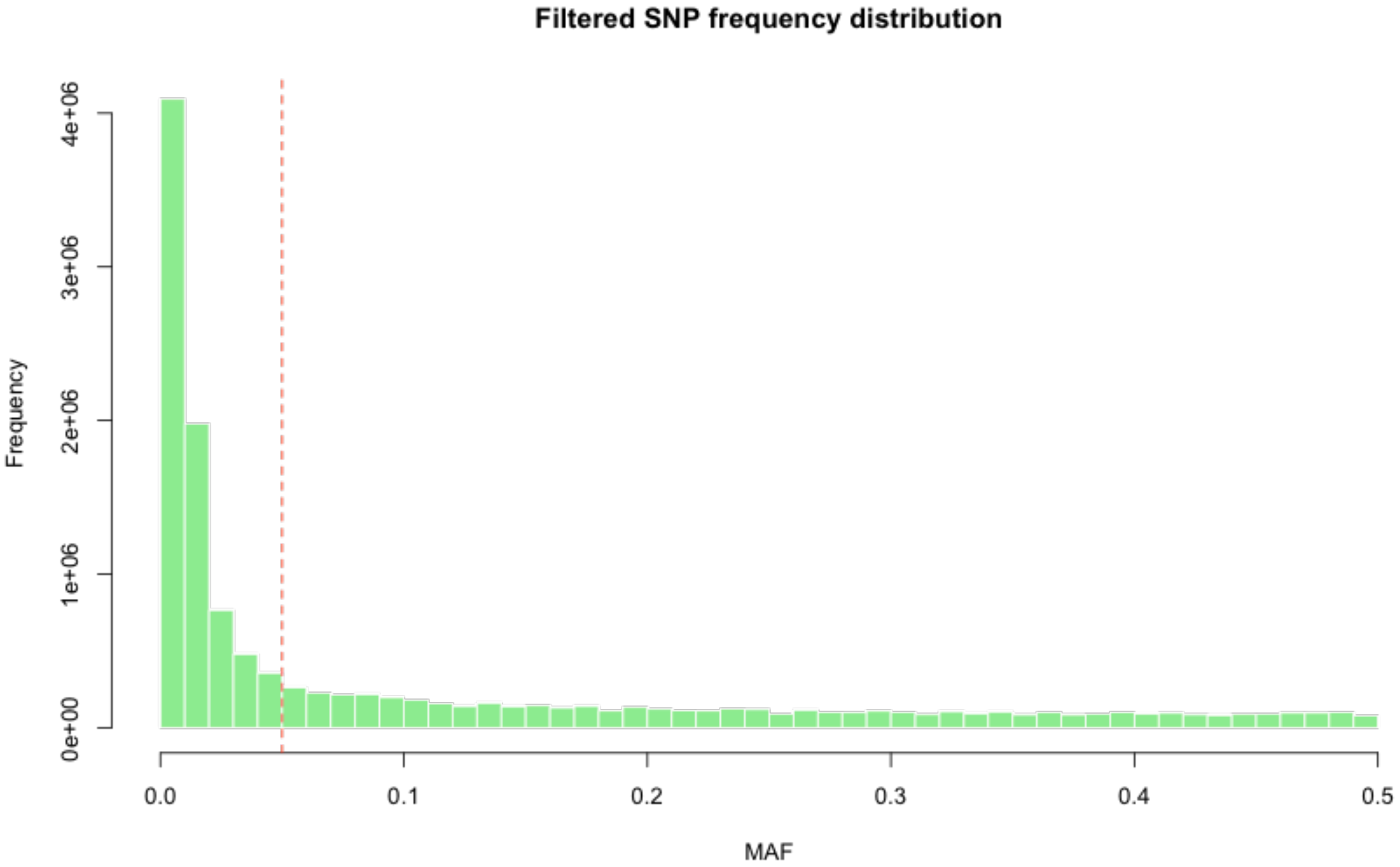

Figure S7. Minor allele frequency spectrum for called SNPs. The x-axis shows the minor frequency in our sample (in 0.1 frequency bins), and the height of each bar corresponds to the number of sites in each bin.

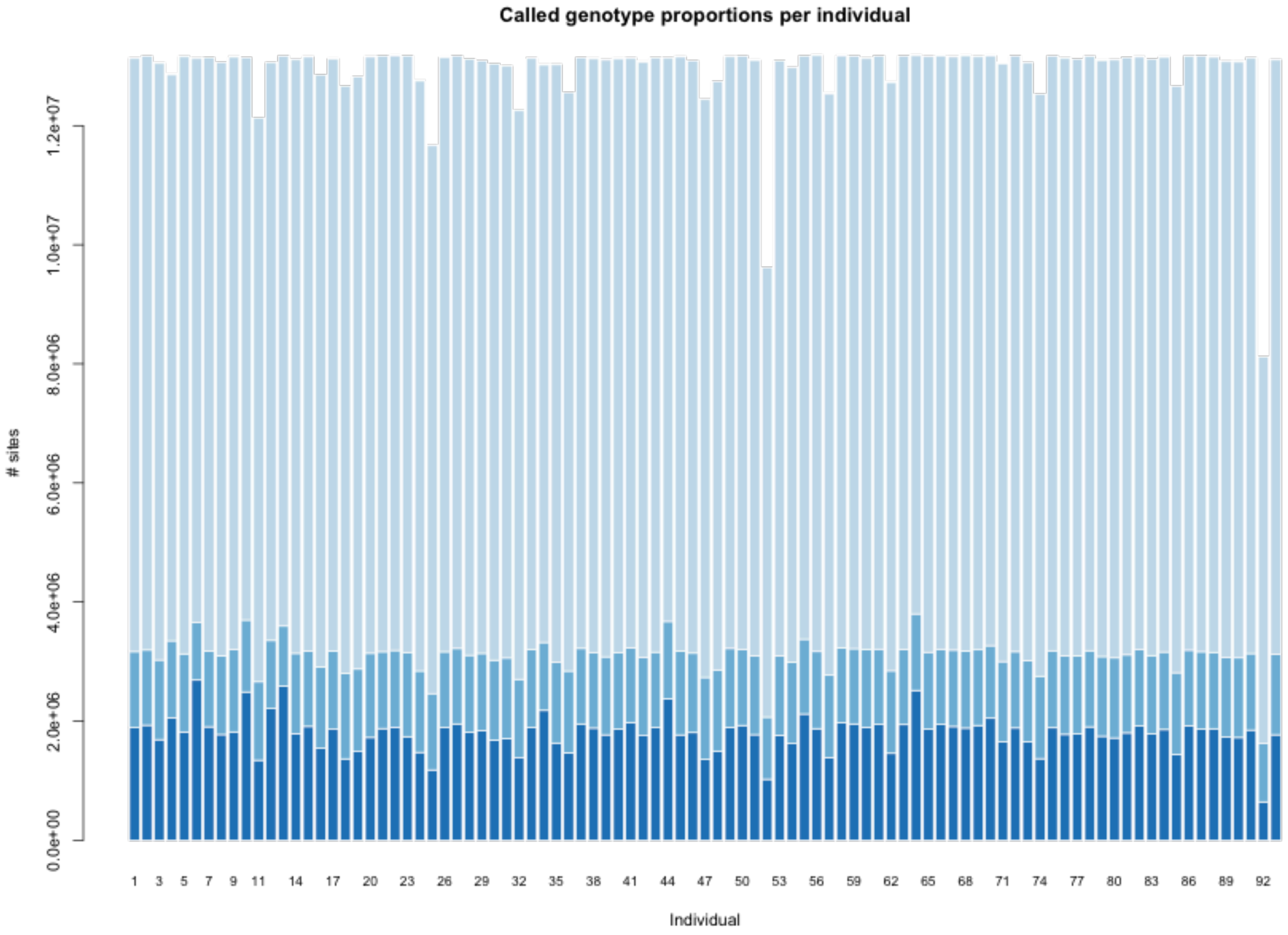

Figure S8. The number of sites (y-axis) called for each individual (x-axis). Stacked bars indicate the number of sites called as homozygote for the alternative allele (dark blue), heterozygous (blue), and homozygous for the reference allele (light blue).

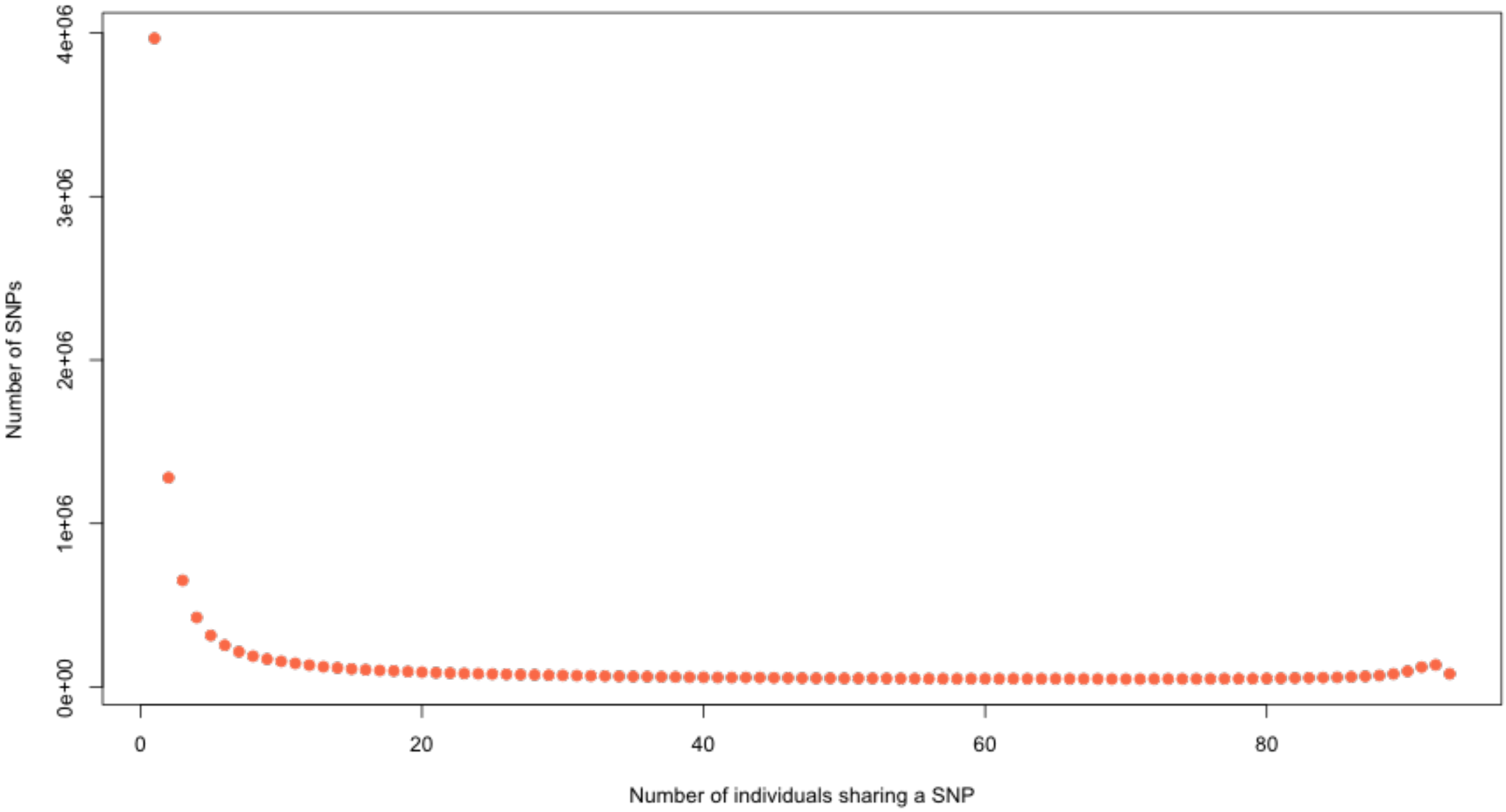

Figure S9. SNP sharing across individuals. The number of variable sites (y-axis) are plotted by the number of individuals that share them (x-axis).

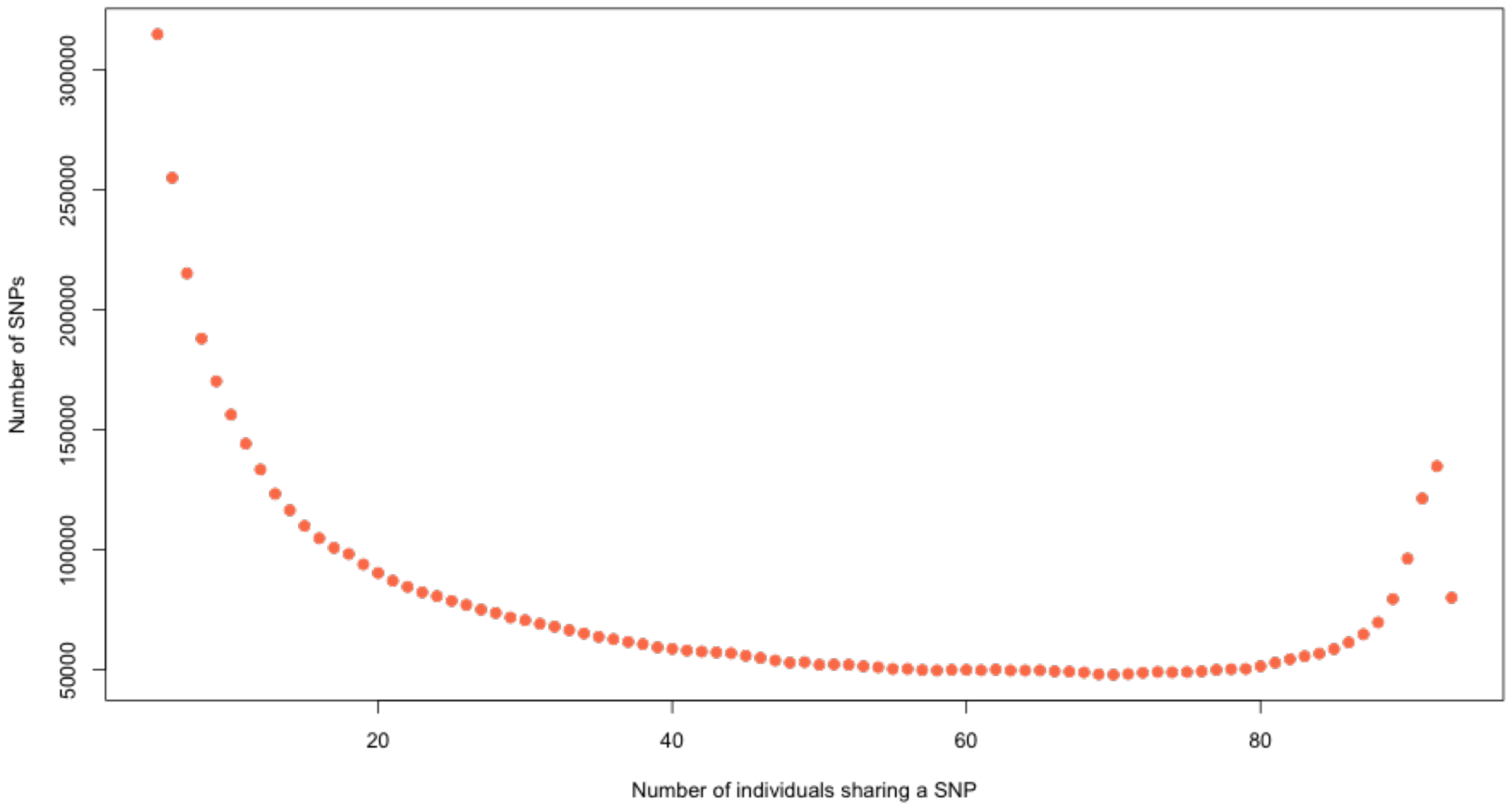

Figure S10. Similar to the previous figure, focusing on sites shared by 10 individuals or more.

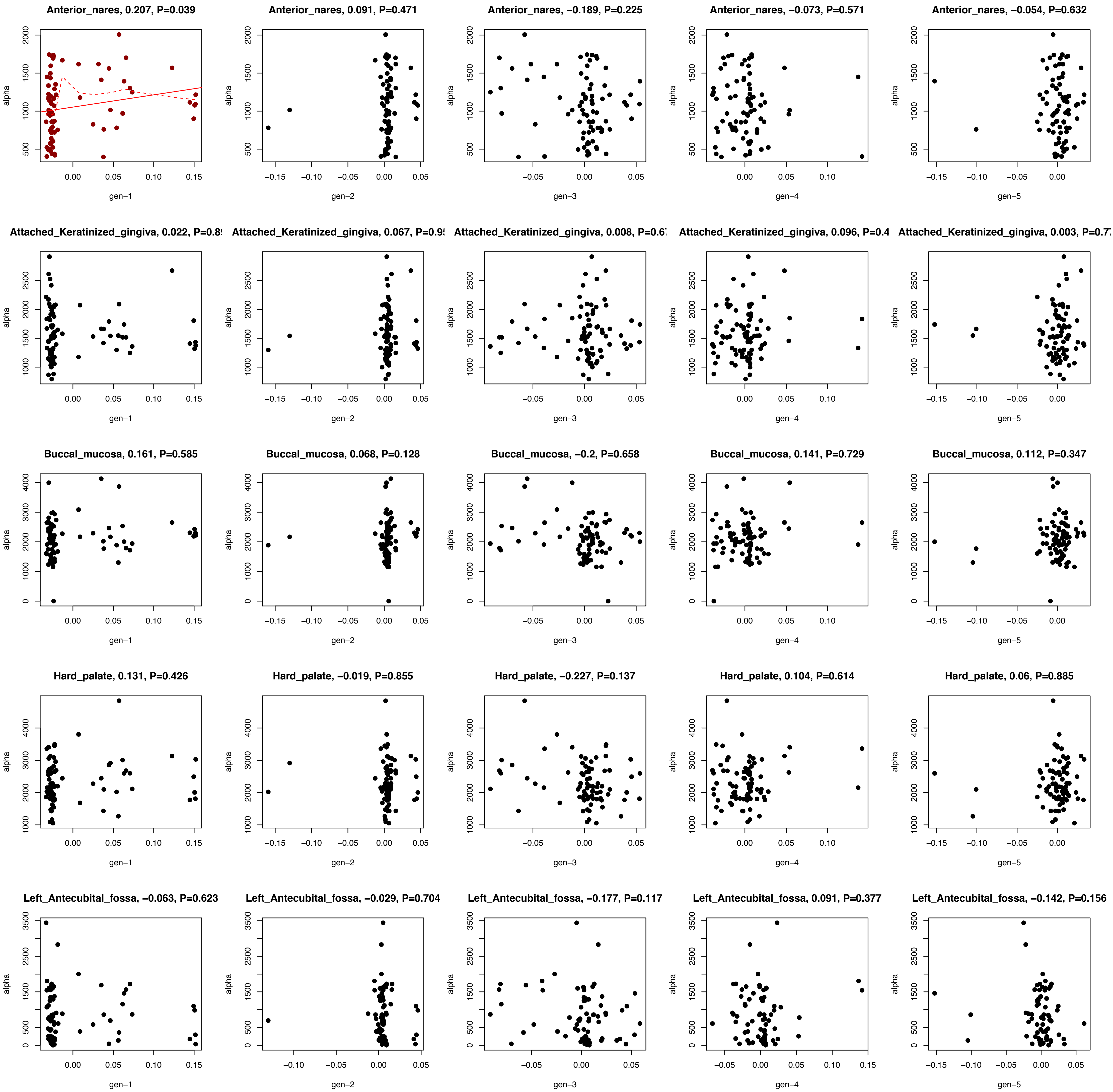

Figure S11. Correlation between host genetic variation PCs and alpha diversity. Each row represents alpha diversity in a specific body site (y-axis), as listed in the title. Each column represents different principal component (x-axis), with the first column to the left showing PC1, the second showing PC2, and so on through PC5.

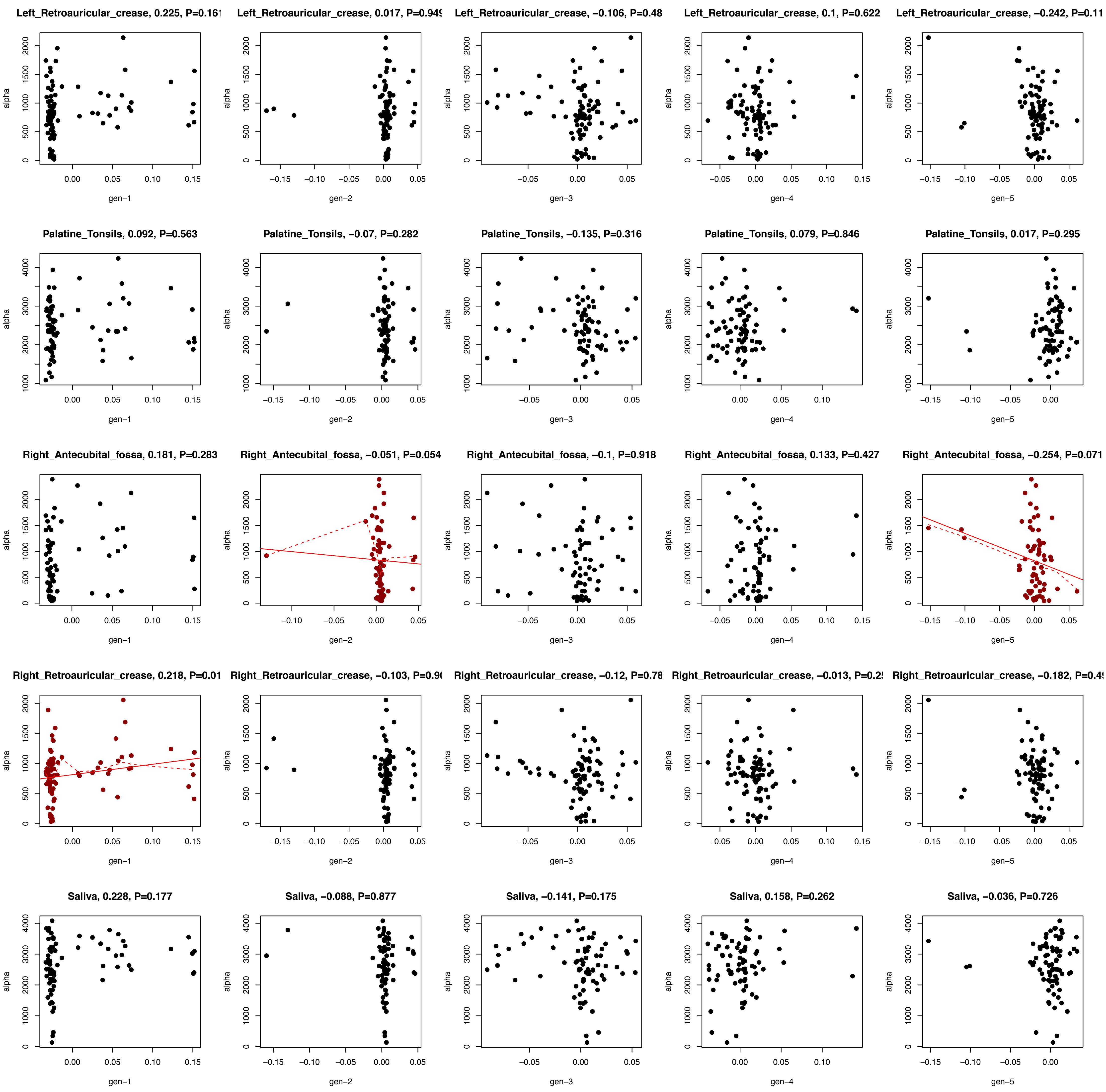

Figure S11. (cont.)

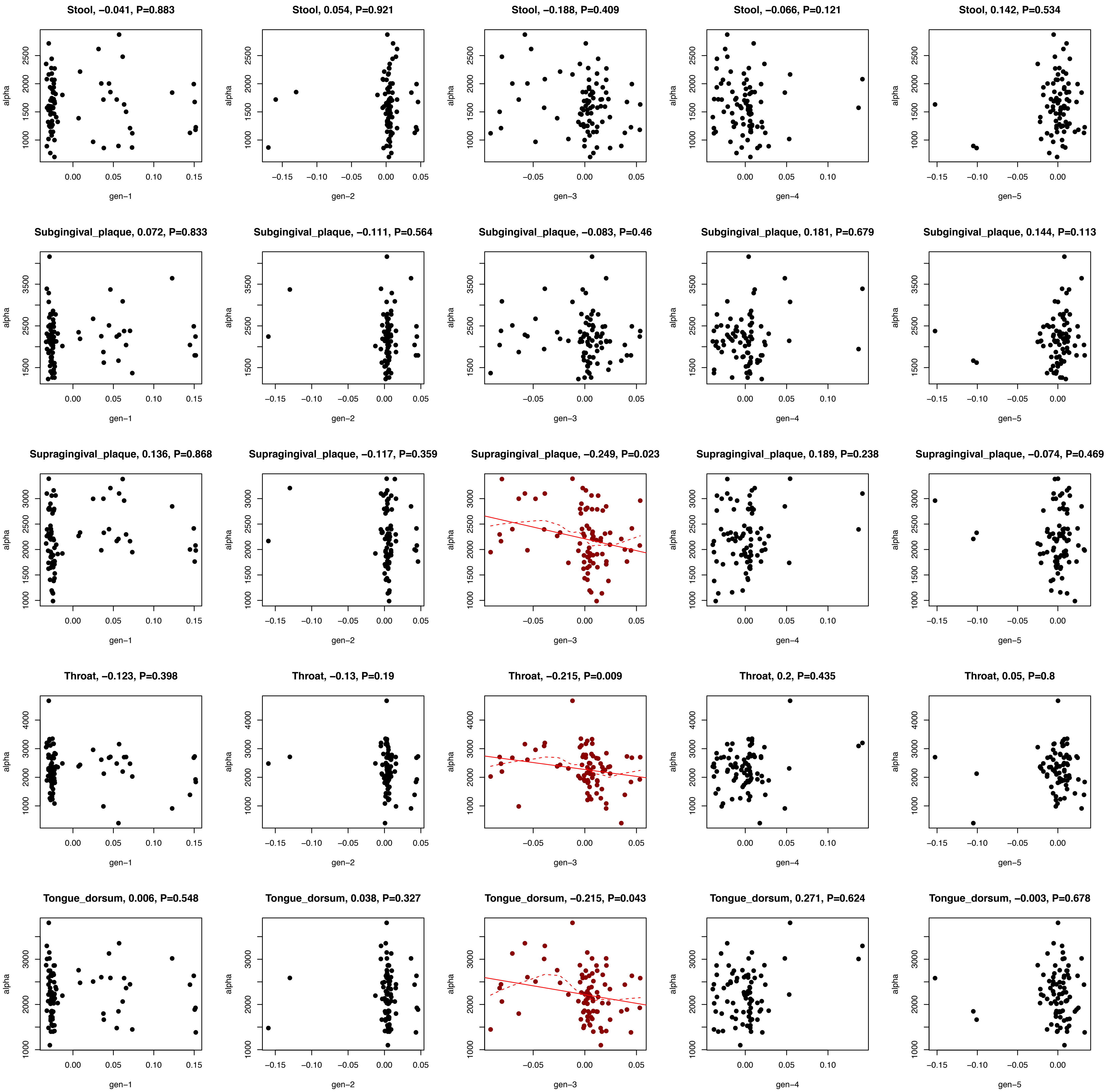

Figure S11. (cont.)

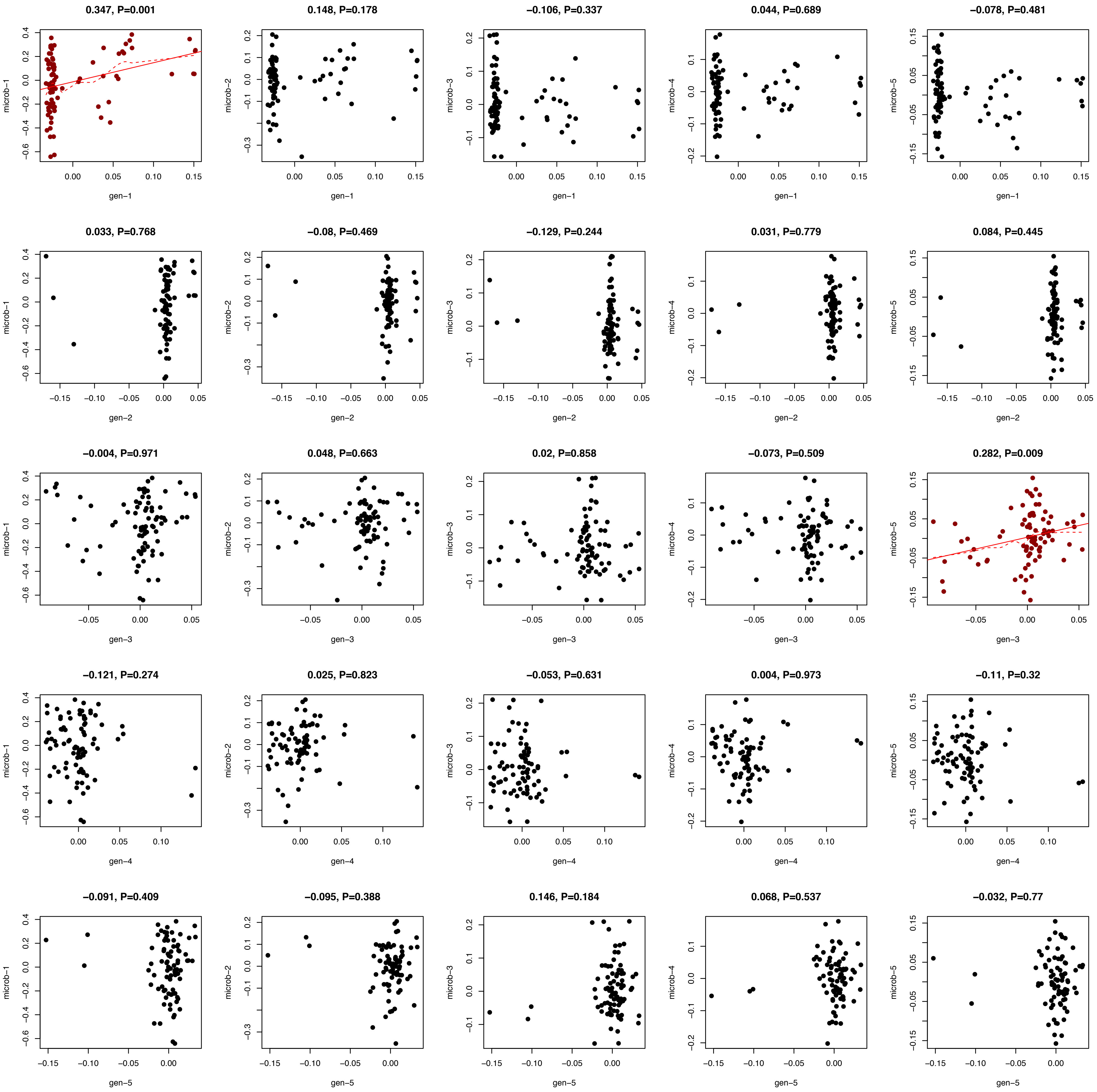

Figure S12. Correlation between host genetic variation (x-axis) and microbiome PCs (y-axis). Each row represents a different host PC (PC1 on top row, PC5 on bottom row), and each column represents a different microbiome PC (PC1 at left and PC5 at right). The correlation coefficient and P-value for the correlation is listed in the title of each figure. This figure shows the stool microbiome.

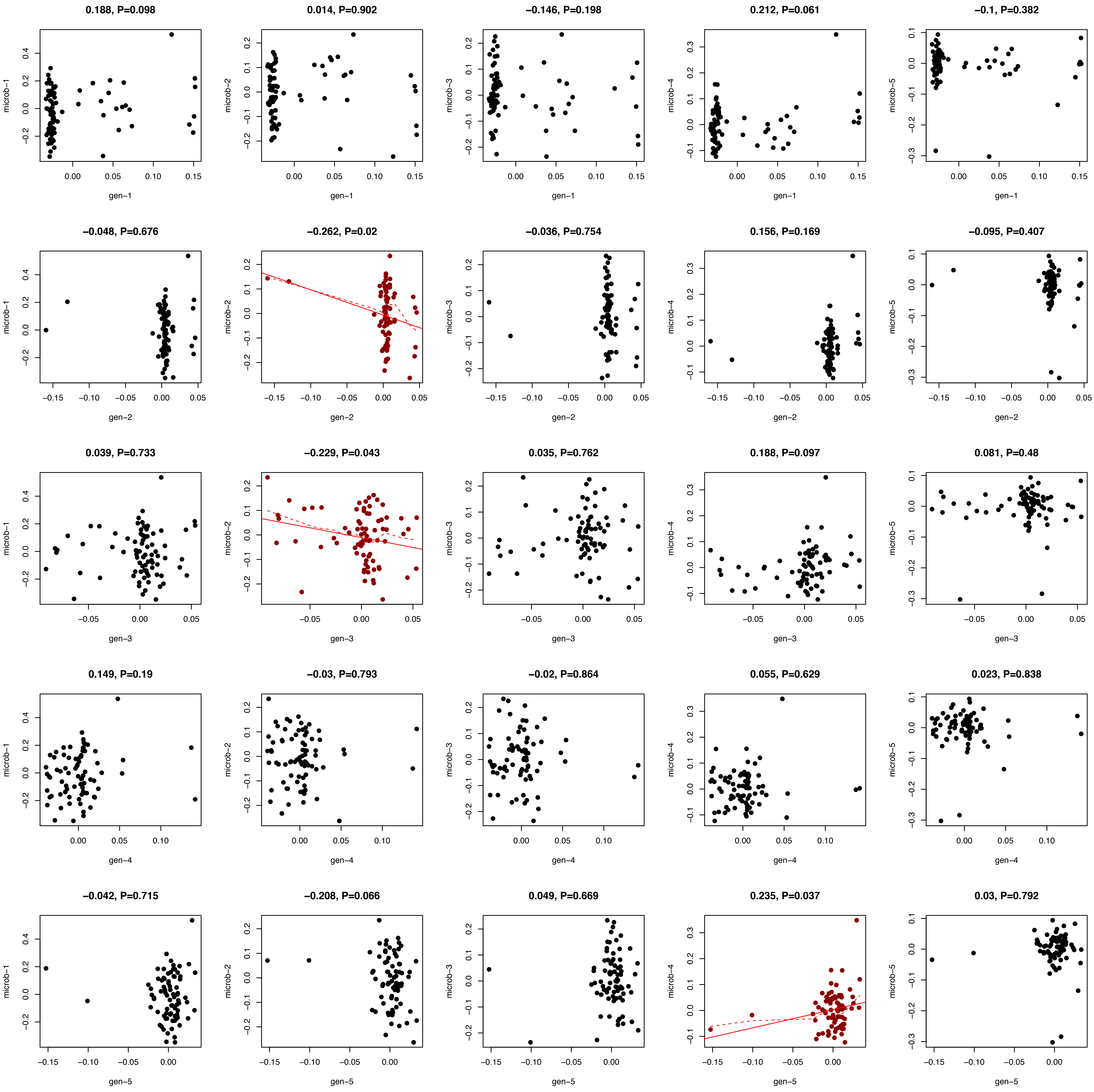

Figure S12. (cont.) Similar to previous page, but showing the throat microbiome data.

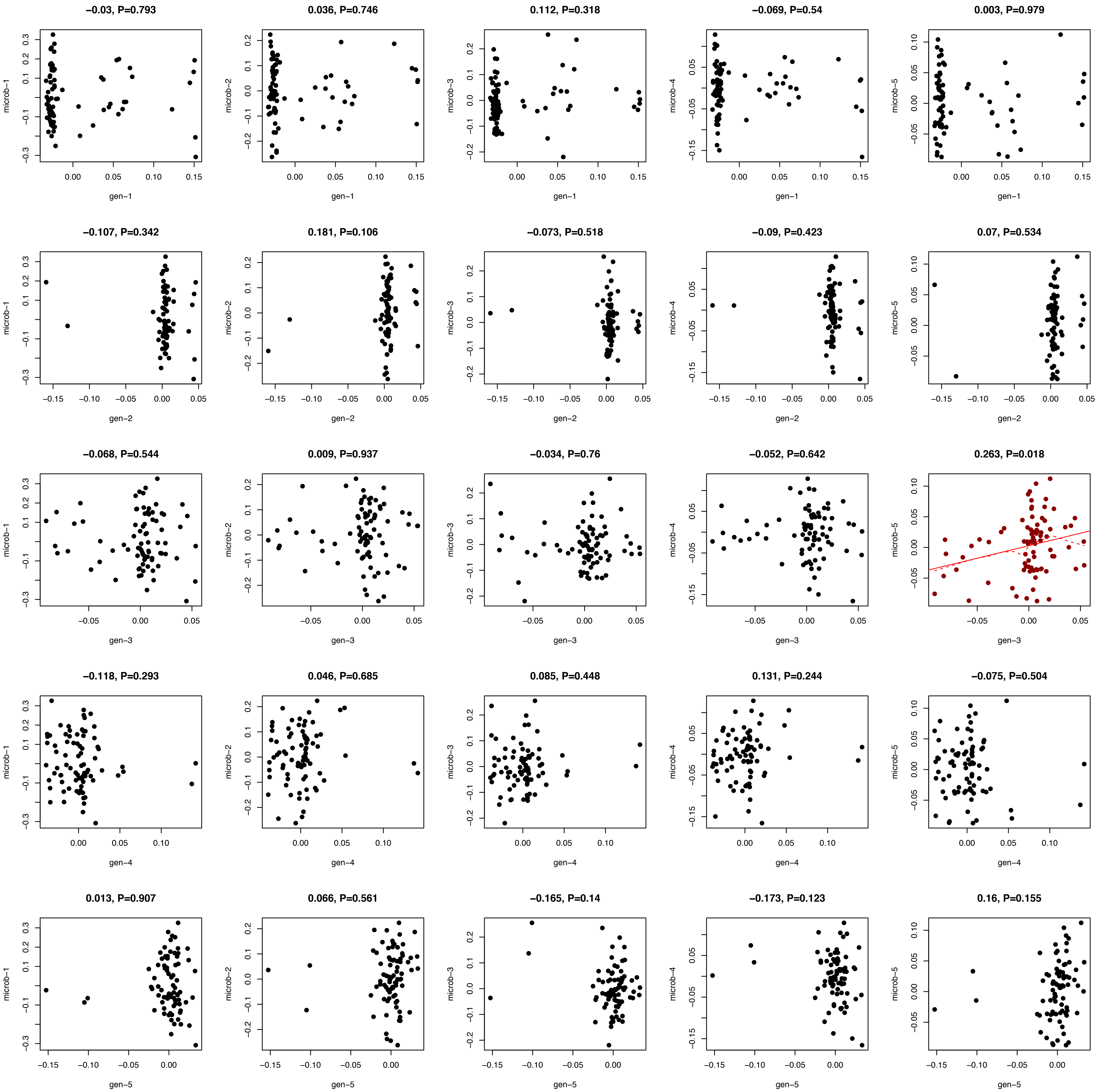

Figure S12. (cont.) Similar to previous page, but showing the Tongue dorsum microbiome data.

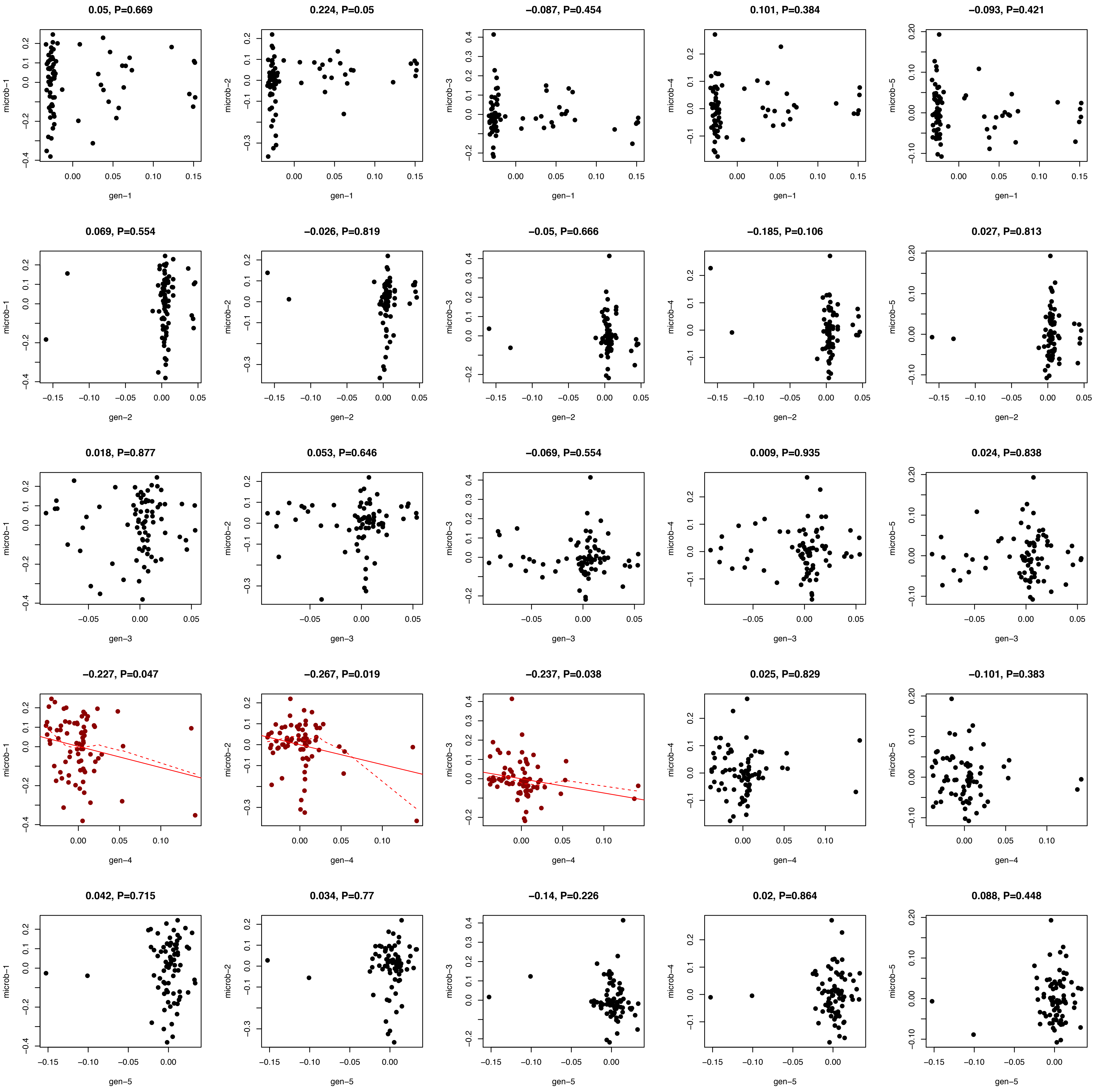

Figure S12. (cont.) Similar to previous page, but showing the Anterior nares microbiome data.

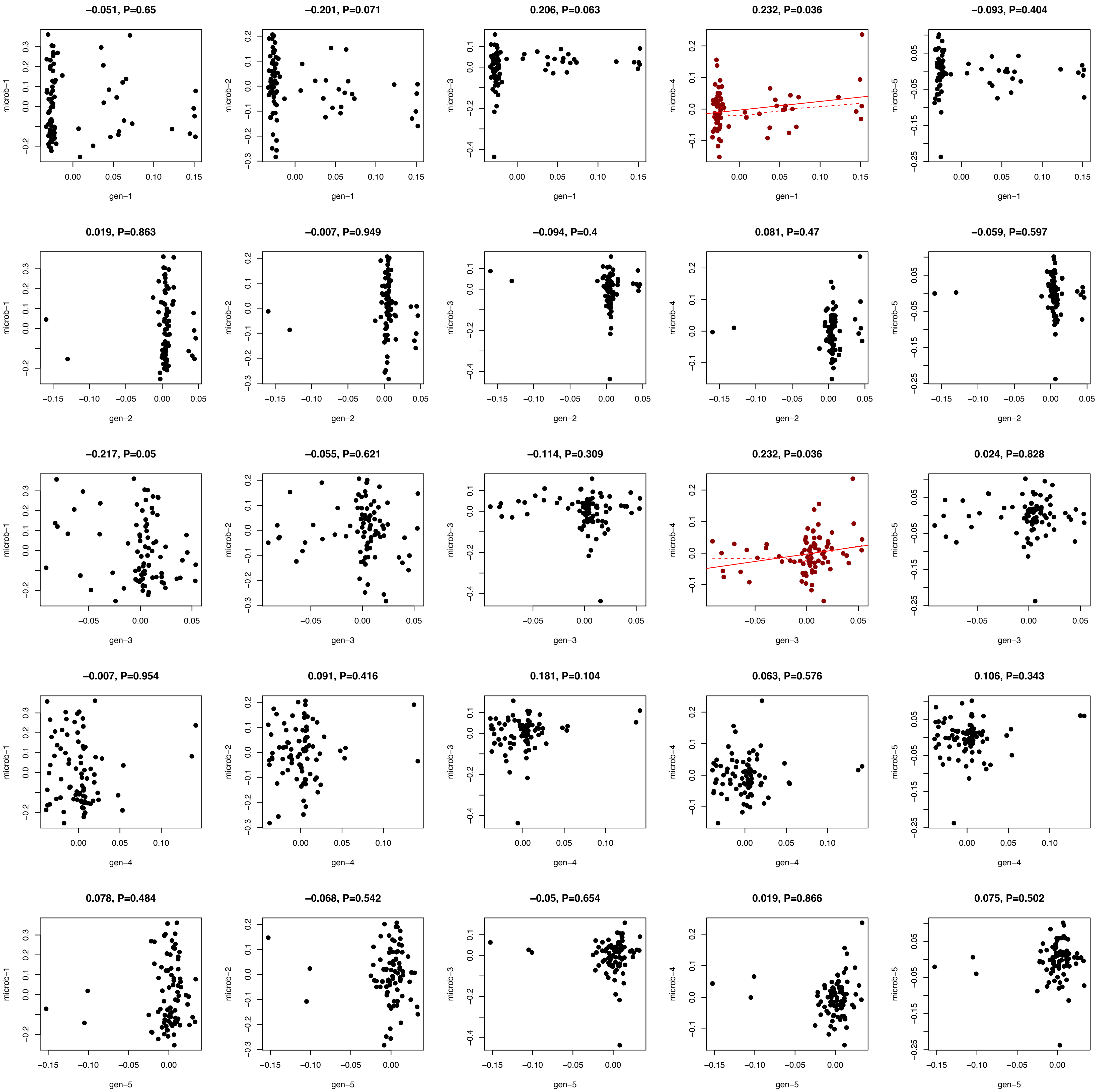

Figure S12. (cont.) Similar to previous page, but showing the Attached keratinized gingiva microbiome data.

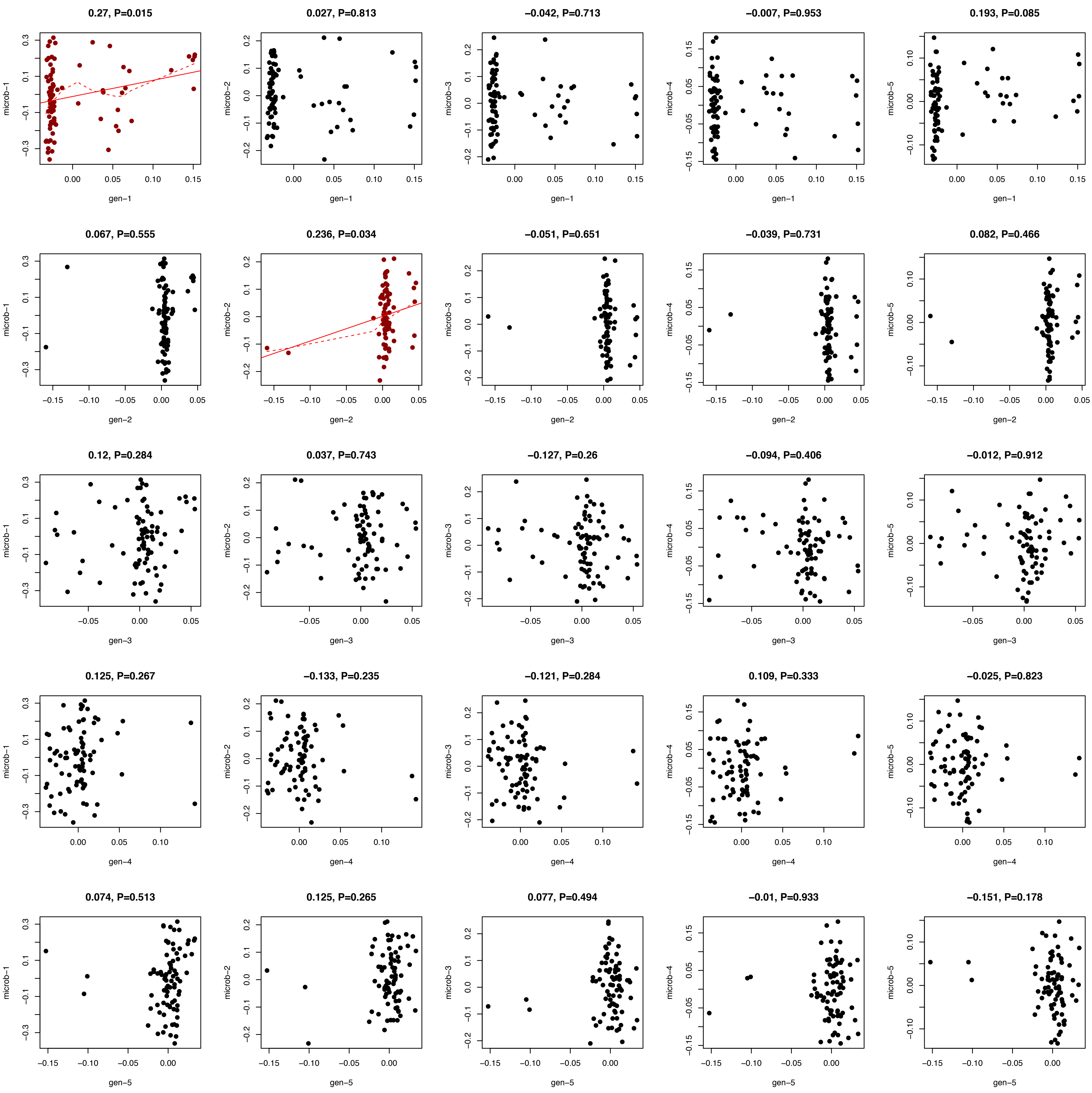

Figure S12. (cont.) Similar to previous page, but showing the Attached Palatine tonsils microbiome data.

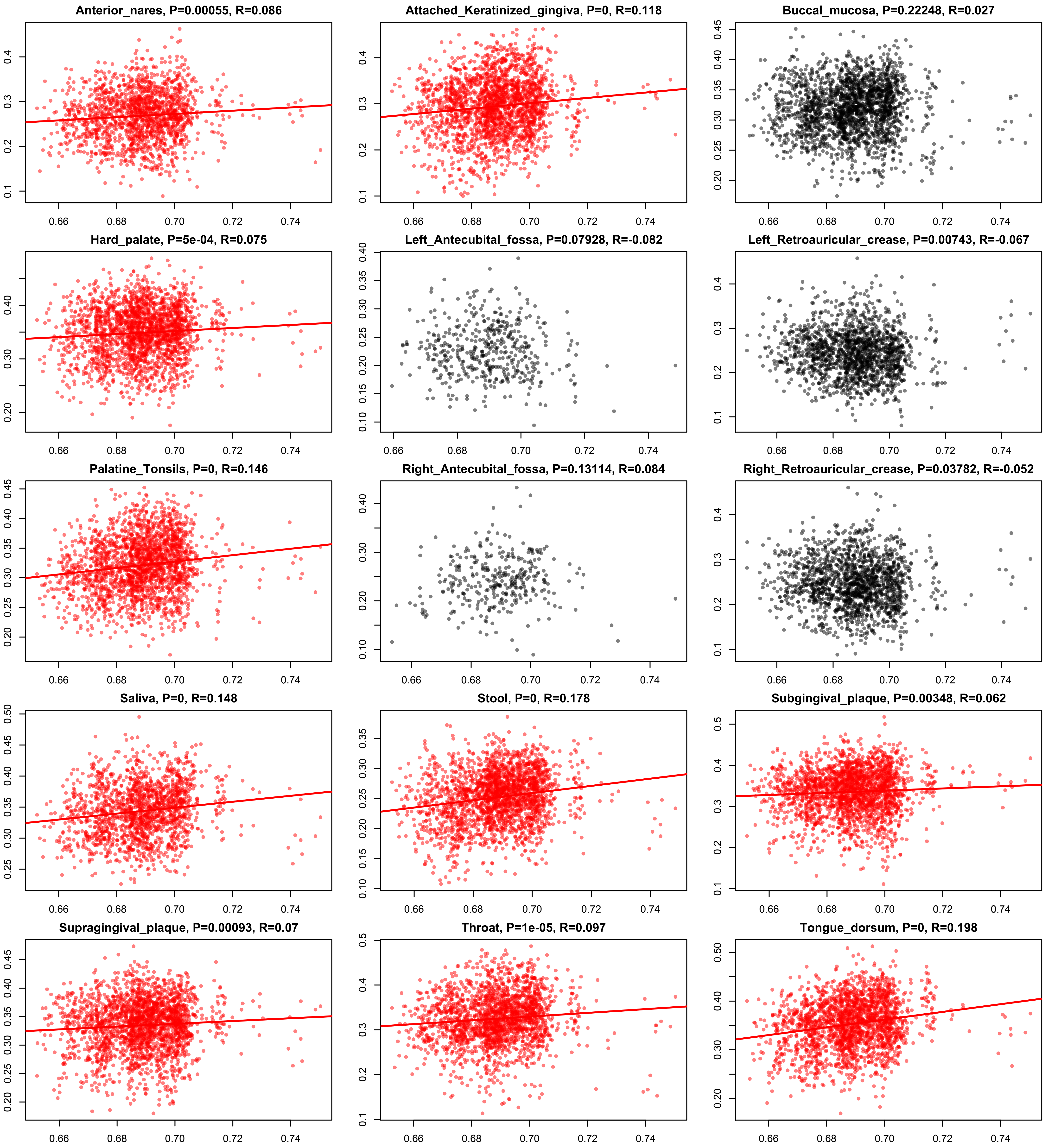

Figure S13. Correlations between host genetic identity-by-state (x-axis) and microbiome beta diversity (inverse, y-axis). Each panel represents microbiome at a different body site, as listed at the title. The title also lists the correlation coefficient and P-value for each panel. The linear regression line is plotted in case the correlation is significant.

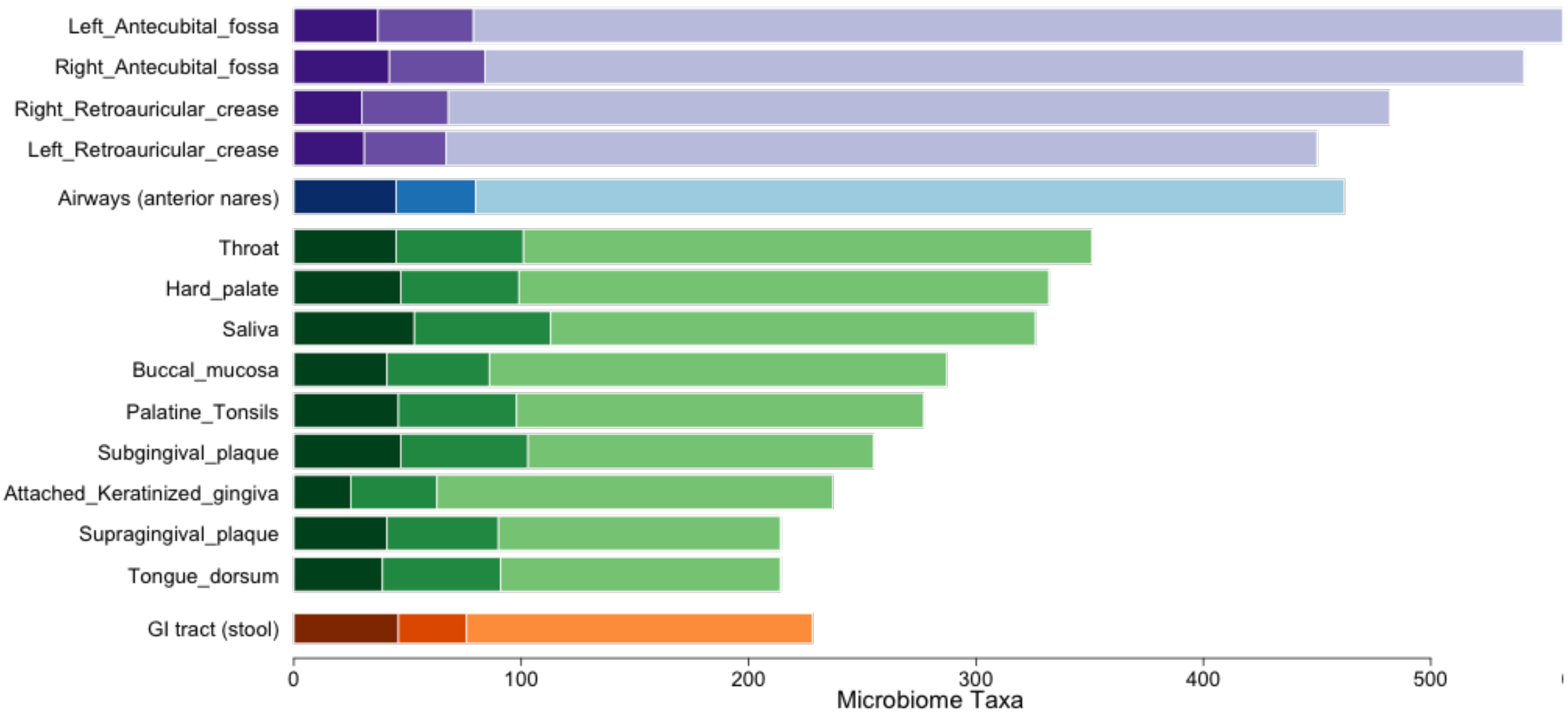

Figure S14. Microbiome and host genetic data used in this study. A breakdown of taxa numbers excluded in each filtering step, depicted by light-to-dark colors: (i) excluded because of sparse data, (ii) excluded because of inter-correlations, (iii) included as traits in final association analysis.

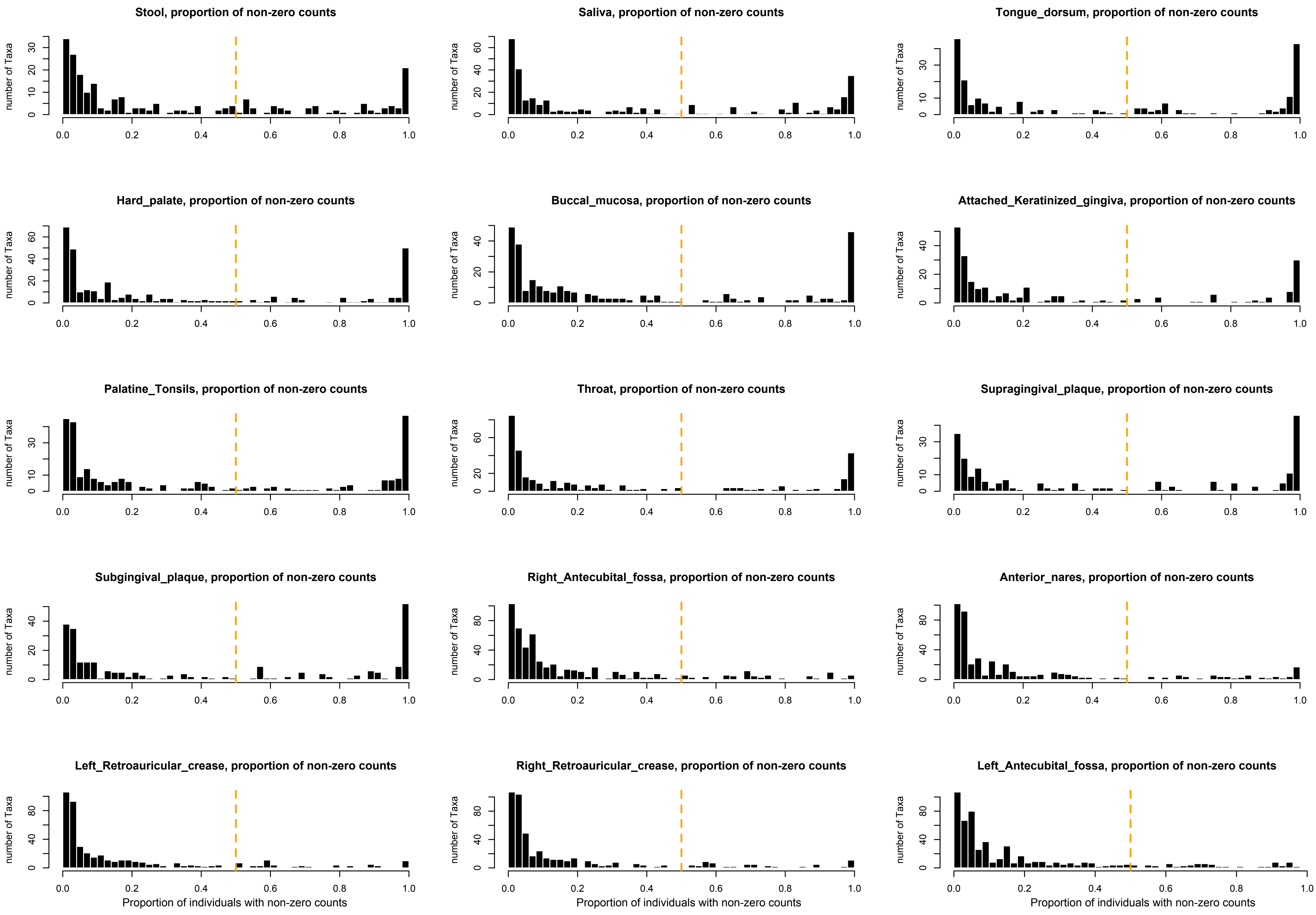

Figure S15. The proportion of individuals with zero-count reads (no data) plotted as a histogram for each body site. Each panel represents data from a single body site. For each taxon, we calculated the proportion of individuals that have a count different than zero, and then, for each body site, we plotted the distribution of these proportions across taxa. For example, at the upper left panel (GI tract data), the leftmost bar represents the fact that about 30 taxa have <2% of individuals with zero counts. The dashed orange line corresponds to the cutoff used in the analysis, where we excluded taxa for which 50% or more of individuals had zero counts.

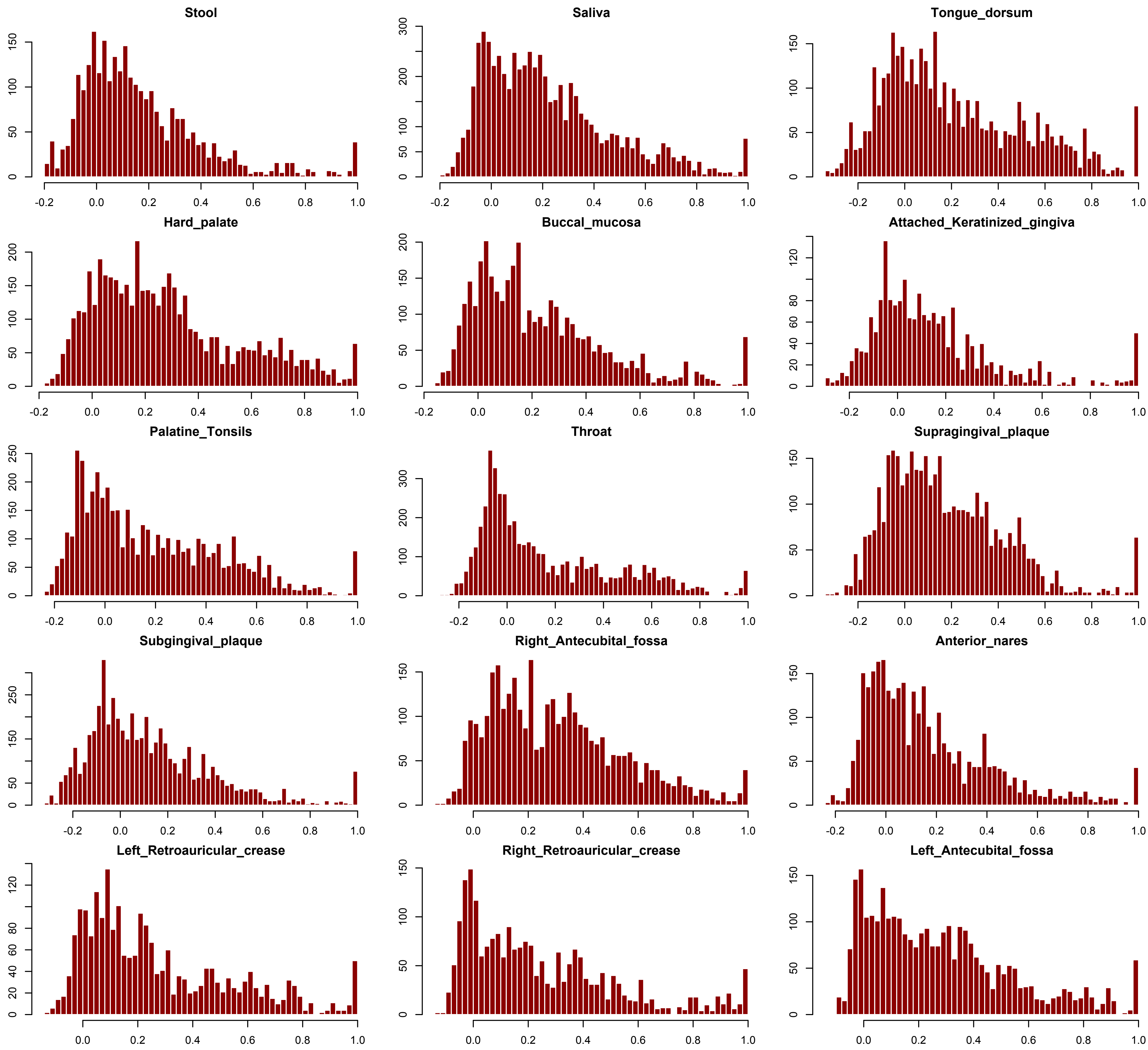

Figure S16. Histograms of pairwise correlation between all pairs of taxa in each body site.

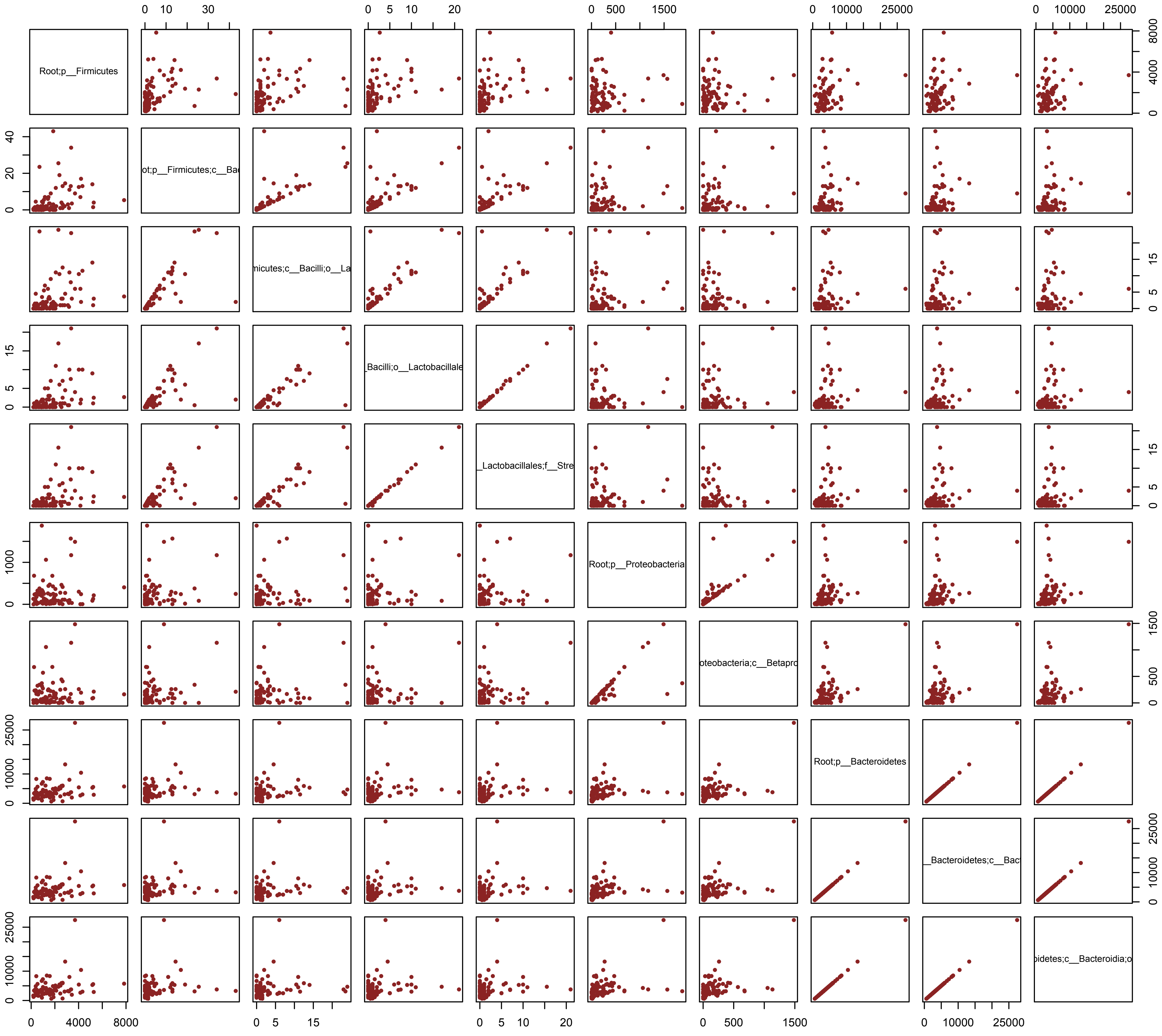

Figure S17. Dotplots of counts showing pairwise correlations among the first 10 taxa in the gut.

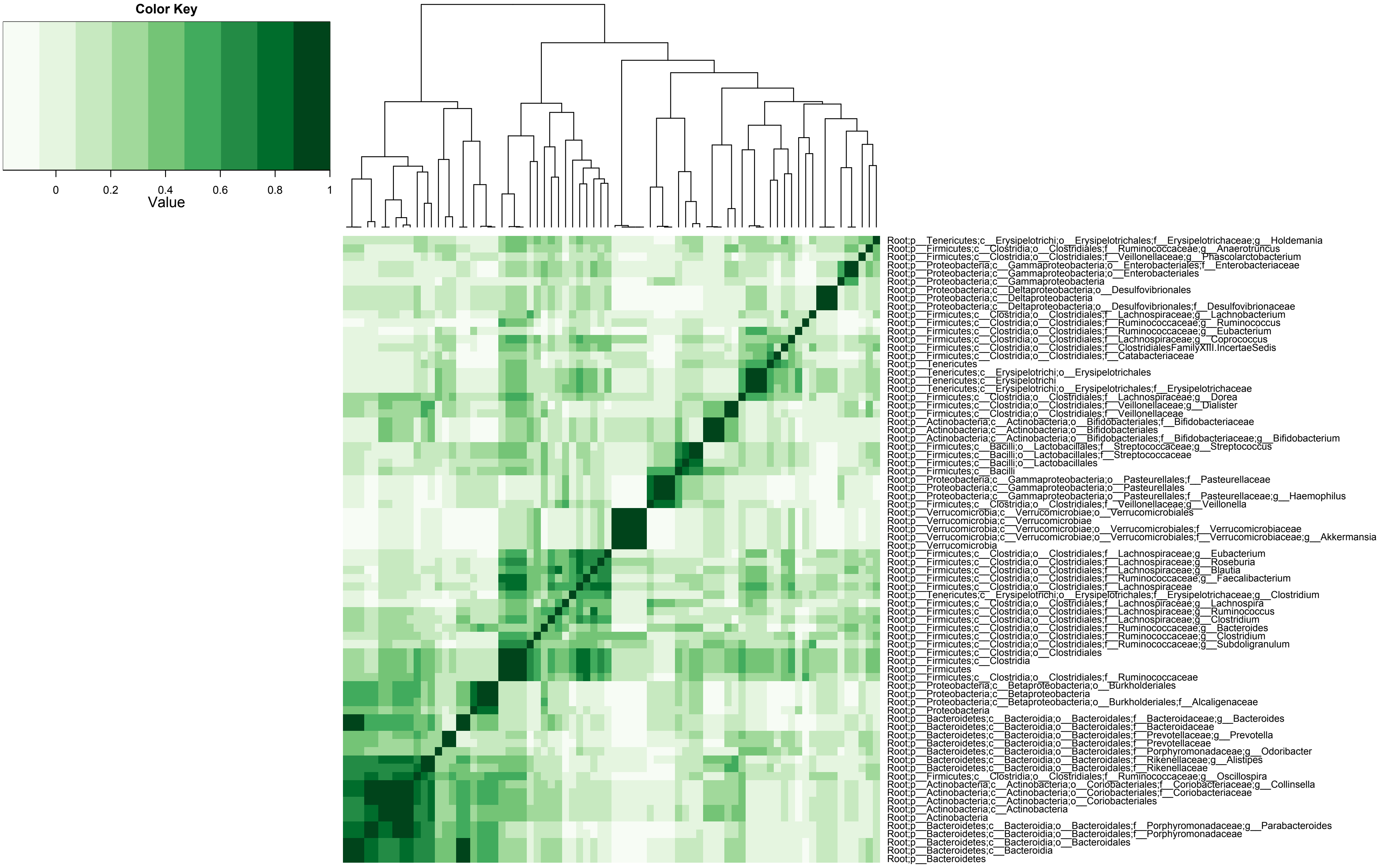

Figure S18. A heatmap of pairwise correlations between all traits in the GI tract. Darker color indicate stronger positive correlation (color key is at the top left corner)

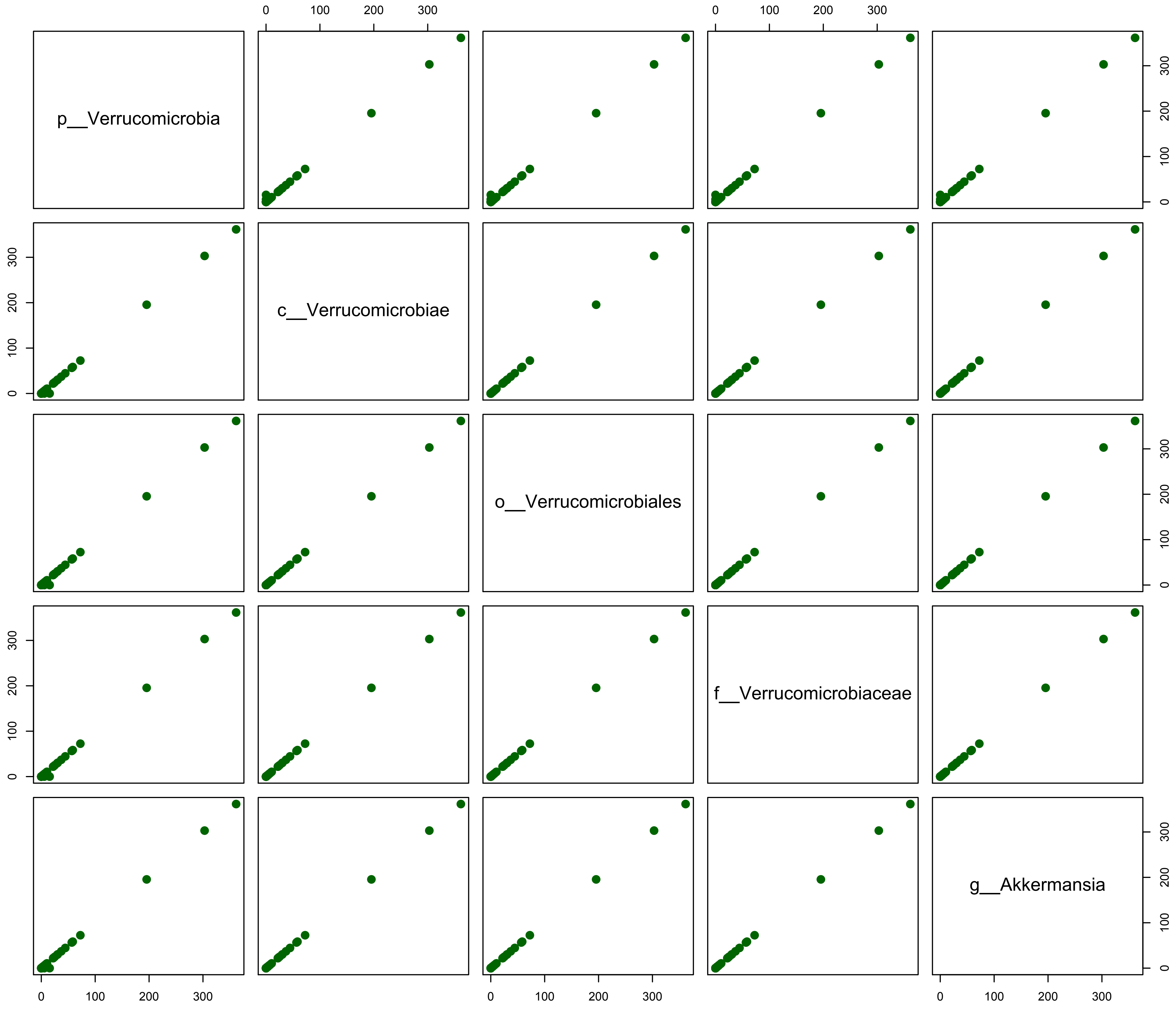

Figure S19. Dotplots showing the pairwise correlations among traits in one of the highly-intercorrelated clusters in the GI tract data. The names of taxa are shown along the diagonal.
