# Supplementary material for: Host genetic variation impacts microbiome composition across human body sites
Source: Genome Biol. 2015 Sep 15;16(1):191. doi: 10.1186/s13059-015-0759-1 (PMC4570153; doi:10.1186/s13059-015-0759-1)
Supplement: Supplementary file 2 — This is a PDF document containing Supplementary Tables S1 through S7. (PDF 622 kb) [file 13059_2015_759_MOESM2_ESM.pdf]

Additional file 2

Table S1. Canonical pathways enriched in genes correlated with microbiome composition

Host pathway	*P*-value	Microbiome taxa
Leptin Signaling in Obesity	2.29×10^{-7}	Veillonella (Anterior nares), Lachnospiraceae (Attached Keratinized gingiva), Haemophilus (Attached Keratinized gingiva), Clostridium (Right Antecubital fossa), Bacteroides (Right Retroauricular crease), Propionibacterium (Subgingival plaque)
G Protein Signaling Mediated by Tubby	9.33×10^{-6}	Bacteroides (Anterior nares), Veillonella (Anterior nares), Lachnospiraceae (Attached Keratinized gingiva), Clostridium (Right Antecubital fossa)
P2Y Purigenic Receptor Signaling Pathway	9.33×10^{-6}	Bacteroides (Anterior nares), Veillonella (Anterior nares), Lachnospiraceae (Attached Keratinized gingiva), Haemophilus (Attached Keratinized gingiva), Clostridium (Right Antecubital fossa), Bacteroides (Right Retroauricular crease), Propionibacterium (Subgingival plaque)
Melatonin Signaling	2.75×10^{-5}	Veillonella (Anterior nares), Lachnospiraceae (Attached Keratinized gingiva), Haemophilus (Attached Keratinized gingiva), Azospira (Buccal mucosa), Clostridium (Right Antecubital fossa), Propionibacterium (Subgingival plaque)
D-myo-inositol (1,4,5)-Trisphosphate Biosynthesis	7.41×10^{-5}	Veillonella (Anterior nares), Lachnospiraceae (Attached Keratinized gingiva), Clostridium (Right Antecubital fossa), Propionibacterium (Subgingival plaque)
UVA-Induced MAPK Signaling	1.23×10^{-4}	Veillonella (Anterior nares), Lachnospiraceae (Attached Keratinized gingiva), Clostridium (Right Antecubital fossa), Propionibacterium (Subgingival plaque), Campylobacter (Supragingival plaque)
CREB Signaling in Neurons	1.23×10^{-4}	Bacteroides (Anterior nares), Veillonella (Anterior nares), Lachnospiraceae (Attached Keratinized gingiva), Haemophilus (Attached Keratinized gingiva), Clostridium (Right Antecubital fossa), Bacteroides (Right Retroauricular crease), Propionibacterium (Subgingival plaque)
Cardiac Hypertrophy Signaling	1.23×10^{-4}	Bacteroides (Anterior nares), Veillonella (Anterior nares), Lachnospiraceae (Attached Keratinized gingiva), Haemophilus (Attached Keratinized gingiva), Clostridium (Right Antecubital fossa), Bacteroides (Right Retroauricular crease), Prevotella (Subgingival plaque), Propionibacterium (Subgingival plaque)
Role of NFAT in Cardiac Hypertrophy	1.62×10^{-4}	Bacteroides (Anterior nares), Veillonella (Anterior nares), Lachnospiraceae (Attached Keratinized gingiva), Haemophilus (Attached Keratinized gingiva), Clostridium (Right Antecubital fossa), Bacteroides (Right Retroauricular crease), Propionibacterium (Subgingival plaque)
Thrombin Signaling	2.23×10^{-4}	Bacteroides (Anterior nares), Veillonella (Anterior nares), Lachnospiraceae (Attached Keratinized gingiva), Haemophilus (Attached Keratinized gingiva), Clostridium (Right Antecubital fossa), Bacteroides (Right Retroauricular crease), Propionibacterium (Subgingival plaque)
Glioblastoma Multiforme Signaling	2.57×10^{-4}	Veillonella (Anterior nares), Lachnospiraceae (Attached Keratinized gingiva), Haemophilus (Attached Keratinized gingiva), Haemophilus (Buccal mucosa), Clostridium (Right Antecubital fossa), Propionibacterium (Subgingival plaque)
JAK/Stat Signaling	2.57×10^{-4}	Haemophilus (Attached Keratinized gingiva), Clostridium (Right Antecubital fossa), Propionibacterium (Subgingival plaque)
Chemokine Signaling	2.95×10^{-4}	Veillonella (Anterior nares), Lachnospiraceae (Attached Keratinized gingiva), Haemophilus (Attached Keratinized gingiva), Clostridium (Right Antecubital fossa), Propionibacterium (Subgingival plaque)
Aldosterone Signaling in Epithelial Cells	3.16×10^{-4}	Veillonella (Anterior nares), Lachnospiraceae (Attached Keratinized gingiva), Haemophilus (Attached Keratinized gingiva), Clostridium (Right Antecubital fossa), Propionibacterium (Subgingival plaque)
CXCR4 Signaling	3.23×10^{-4}	Bacteroides (Anterior nares), Veillonella (Anterior nares), Haemophilus (Attached Keratinized gingiva), Clostridium (Right Antecubital fossa), Bacteroides (Right Retroauricular crease), Propionibacterium (Subgingival plaque)
Renin-Angiotensin Signaling	3.31×10^{-4}	Lachnospiraceae (Attached Keratinized gingiva), Haemophilus (Attached Keratinized gingiva), Clostridium (Right Antecubital fossa), Bacteroides (Right Retroauricular crease), Propionibacterium (Subgingival plaque)
Sphingosine-1-phosphate Signaling	3.46×10^{-4}	Veillonella (Anterior nares), Lachnospiraceae (Attached Keratinized gingiva), Clostridium (Right Antecubital fossa), Bacteroides (Right Retroauricular crease), Propionibacterium (Subgingival plaque)
Gap Junction Signaling	3.63×10^{-4}	Veillonella (Anterior nares), Lachnospiraceae (Attached Keratinized gingiva), Haemophilus (Attached Keratinized gingiva), Clostridium (Right Antecubital fossa), Bacteroides (Right Retroauricular crease), Propionibacterium (Subgingival plaque)
Prolactin Signaling	3.8×10^{-4}	Lachnospiraceae (Attached Keratinized gingiva), Haemophilus (Attached Keratinized gingiva), Clostridium (Right Antecubital fossa), Propionibacterium (Subgingival plaque)
CCR3 Signaling in Eosinophils	4.37×10^{-4}	Bacteroides (Anterior nares), Veillonella (Anterior nares), Haemophilus (Attached Keratinized gingiva), Clostridium (Right Antecubital fossa), Propionibacterium (Subgingival plaque)
14-3-3-mediated Signaling	4.78×10^{-4}	Veillonella (Anterior nares), Lachnospiraceae (Attached Keratinized gingiva), Haemophilus (Attached Keratinized gingiva), Clostridium (Right Antecubital fossa), Propionibacterium (Subgingival plaque)
PDGF Signaling	4.9×10^{-4}	Lachnospiraceae (Attached Keratinized gingiva), Haemophilus (Attached Keratinized gingiva), Streptococcus (Palatine Tonsils), Clostridium (Right Antecubital fossa), Propionibacterium (Subgingival plaque)
p70S6K Signaling	5.5×10^{-4}	Veillonella (Anterior nares), Lachnospiraceae (Attached Keratinized gingiva), Haemophilus (Attached Keratinized gingiva), Clostridium (Right Antecubital fossa), Propionibacterium (Subgingival plaque)
PPARα/RXRα Activation	7.59×10^{-4}	Veillonella (Anterior nares), Lachnospiraceae (Attached Keratinized gingiva), Haemophilus (Attached Keratinized gingiva), Clostridium (Right Antecubital fossa), Bacteroides (Right Retroauricular crease), Propionibacterium (Subgingival plaque)
PI3K Signaling in B Lymphocytes	7.76×10^{-4}	Veillonella (Anterior nares), Lachnospiraceae (Attached Keratinized gingiva), Haemophilus (Attached Keratinized gingiva), Clostridium (Right Antecubital fossa), Streptococcus (Saliva), Propionibacterium (Subgingival plaque)

Table S2. Enrichment of pathways of interest from table S1 using various cutoffs for P-value and distance from genes (D). Each column describes a cutoff combination, with the number of genes tested in the header. Each cell lists the pathway enrichment P-value and the rank in parentheses.

Pathway	$P \leq 10^{-6}$; $D \leq 50k$ (203 genes)	$P \leq 10^{-6}$; $D \leq 20k$ (138 genes)	$P \leq 10^{-6}$; $D \leq 5k$ (90 genes)	$P \leq 5\times10^{-7}$; $D \leq 50k$ (105 genes)	$P \leq 5\times10^{-7}$; $D \leq 20k$ (70 genes)
Leptin signaling in Obesity	2.29×10^{-7} (1)	7.58×10^{-5} (2)	1.44×10^{-5} (2)	1.86×10^{-5} (2)	2.95×10^{-4} (2)
Melatonin Signaling	2.75×10^{-5} (4)	0.063 (83)	0.033 (75)	3.46×10^{-3} (24)	--
JAK/Stat Signaling	2.57×10^{-4} (12)	7.08×10^{-3} (21)	0.031 (70)	3.09×10^{-3} (21)	0.017 (34)
Chemokine Signaling	2.95×10^{-4} (13)	7.76×10^{-3} (23)	2.88×10^{-3} (15)	3.39×10^{-3} (23)	0.018 (39)
CXCR4 Signaling	3.23×10^{-4} (15)	1.34×10^{-3} (7)	3.72×10^{-3} (18)	4.47×10^{-3} (29)	0.011 (21)
Role of Pattern Recognition Receptors in Recognition of Bacteria and Viruses	0.047 (95)	0.018 (38)	7.24×10^{-3} (33)	8.51×10^{-3} (43)	3.09×10^{-3} (10)

Table S3. GO categories enriched in host genes correlated with microbiome composition using InnateDB					
**GO term Name**	**GO term ID**	**Genes Ratio**	**GO term P-value**	**GO term P-value (corrected)**	**Gene Symbols**
interleukin-12-mediated signaling pathway [biological_process]	GO:0035722	67%	0.00020	0.05683	JAK2 ; PLCB1 ;
phosphoric diester hydrolase activity [molecular_function]	GO:0008081	12%	0.00015	0.05746	PLCB1 ; PLCB4 ; PLCG2 ; PLCZ1 ;
androgen receptor signaling pathway [biological_process]	GO:0030521	9%	0.00044	0.06094	CCNE1 ; CDK7 ; FKBP4 ; THRAP3 ;
mitotic cell cycle [biological_process]	GO:0000278	3%	0.00039	0.06254	CCNE1 ; CDCA8 ; CDK5RAP2 ; CDK7 ; CEP192 ; FEN1 ; MAD1L1 ; POLE ; SEH1L ; ZWINT ;
nucleus [cellular_component]	GO:0005634	1%	0.00011	0.06331	ANKRD1 ; BCL10 ; CABLES1 ; CACNA1A ; CBLB ; CCNE1 ; CDCA8 ; CDK7 ; CEBPZ ; CTBP2 ; ETHE1 ; FADS1 ; FEN1 ; FKBP4 ; FOXM1 ; JAK2 ; KAZN ; KDM5B ; KLF15 ; LANCL1 ; LMCD1 ; LPP ; LYRM4 ; MAD1L1 ; MAP2K2 ; MDFIC ; METTL3 ; NARG2 ; NRIP2 ; OSBPL1A ; PAK7 ; PARP4 ; PHAX ; PIAS4 ; PLCB1 ; PLCB4 ; PLCZ1 ; POLE ; RAB2B ; RORA ; RSPO1 ; SALL2 ; SH3BGRL2 ; SLC25A10 ; STK40 ; TFAP2A ; THRAP3 ; TOX4 ; XPA ; XRCC1 ; ZBTB7A ; ZFP28 ; ZNF148 ; ZNF165 ; ZNF343 ; ZNF470 ; ZNF555 ; ZNF556 ; ZNF57 ; ZNF575 ; ZNF71 ; ZNF77 ; ZNF786 ; ZSCAN16 ; ZWINT ;
phospholipase C activity [molecular_function]	GO:0004629	18%	0.00035	0.06495	PLCB4 ; PLCG2 ; PLCZ1 ;
sulfide oxidation, using sulfide:quinone oxidoreductase [biological_process]	GO:0070221	40%	0.00067	0.06814	ETHE1 ; SLC25A10 ;
intracellular [cellular_component]	GO:0005622	1%	0.00074	0.06837	ADCY5 ; ARL16 ; BCL10 ; CAPN13 ; CLNK ; EIF2AK2 ; FKBP4 ; HGS ; KDM5B ; KLF15 ; MRPL12 ; OSBPL1A ; PLCB4 ; PLCZ1 ; PREX1 ; RAB2B ; RANBP17 ; SALL2 ; SLC25A10 ; ZBTB7A ; ZFP28 ; ZNF148 ; ZNF165 ; ZNF343 ; ZNF470 ; ZNF555 ; ZNF556 ; ZNF57 ; ZNF575 ; ZNF71 ; ZNF77 ; ZNF786 ; ZSCAN16 ;
ion transport [biological_process]	GO:0006811	3%	0.00086	0.07326	CACNA1A ; GABRR1 ; GABRR2 ; KCNH1 ; P2RX2 ; RYR3 ; SLC1A2 ; SLC24A3 ; SLC25A10 ;
gamma-aminobutyric acid signaling pathway [biological_process]	GO:0007214	14%	0.00067	0.07439	CACNA1A ; GABRR1 ; GABRR2 ;
intracellular signal transduction [biological_process]	GO:0035556	3%	0.00034	0.07561	ADCY5 ; CBLB ; CLNK ; JAK2 ; PLCB1 ; PLCB4 ; PLCG2 ; PLCZ1 ; PREX1 ; SOCS6 ;
intracellular membrane-bounded organelle [cellular_component]	GO:0043231	3%	0.00064	0.07921	FADS1 ; HGS ; KAZN ; KDM5B ; LANCL1 ; MIB1 ; PREX1 ; SYT2 ; TFAP2A ; THRAP3 ; ZWINT ;
mitotic metaphase [biological_process]	GO:0000089	33%	0.00100	0.07986	CDCA8 ; MAD1L1 ;
sulfur amino acid metabolic process [biological_process]	GO:0000096	12%	0.00127	0.09405	CACNA1A ; ETHE1 ; SLC25A10 ;

Table S4. Pathways enriched in host genes correlated with microbiome composition using InnateDB

Pathway Name	Pathway Database	Genes Ratio	Pathway P-value	Pathway P-value (corrected)	Gene Symbols
Inositol phosphate metabolism	KEGG	9%	0.00012	0.02097	PIK3C2G ; PLCB1 ; PLCB4 ; PLCG2 ; PLCZ1 ;
Base excision repair	KEGG	12%	0.00017	0.01468	FEN1 ; PARP4 ; POLE ; XRCC1 ;
GABA A (rho) receptor activation	REACTOME	67%	0.00022	0.01234	GABRR1 ; GABRR2 ;
Inositol phosphate metabolism (Inositol phosphate metabolism)	INOH	10%	0.00045	0.01893	PLCB1 ; PLCB4 ; PLCG2 ; PLCZ1 ;
Phosphatidylinositol signaling system	KEGG	7%	0.00046	0.01532	PIK3C2G ; PLCB1 ; PLCB4 ; PLCG2 ; PLCZ1 ;
Calcium signaling pathway	KEGG	4%	0.00066	0.01847	CACNA1A ; P2RX2 ; PLCB1 ; PLCB4 ; PLCG2 ; PLCZ1 ; RYR3 ;
Sulfide oxidation to sulfate	REACTOME	40%	0.00073	0.01743	ETHE1 ; SLC25A10 ;
PLC beta signaling (GPCR GroupI metabotropic glutamate receptor signaling pathway)	INOH	29%	0.00151	0.02814	PLCB1 ; PLCB4 ;
PLC beta signaling (GPCR signaling (G alpha q))	INOH	29%	0.00151	0.02814	PLCB1 ; PLCB4 ;
Heterotrimeric GPCR signaling pathway (through glutamate, G alpha q and PLC beta) (GPCR GroupI metabotropic glutamate receptor signaling pathway)	INOH	11%	0.00174	0.02929	MAP2K2 ; PLCB1 ; PLCB4 ;
KitReceptor	NETPATH	5%	0.00200	0.03057	CBLB ; JAK2 ; MAP2K2 ; PLCG2 ; SOCS6 ;
Fmlp induced chemokine gene expression in hmc-1 cells	PID BIOCARTA	10%	0.00213	0.02988	MAP2K2 ; PIK3C2G ; PLCB1 ;
BCR	NETPATH	4%	0.00246	0.03175	BCL10 ; CBLB ; CCNE1 ; CDK7 ; MAP2K2 ; PLCG2 ;
Heterotrimeric GPCR signaling pathway (through Adenosine G alpha s) (GPCR Adenosine A2A receptor signaling pathway)	INOH	9%	0.00307	0.03683	ADCY5 ; CBLB ; MAP2K2 ;
Long-term depression	KEGG	6%	0.00308	0.03447	CACNA1A ; MAP2K2 ; PLCB1 ; PLCB4 ;
Synthesis of bile acids and bile salts via 24-hydroxycholesterol	REACTOME	20%	0.00318	0.03139	CYP46A1 ; CYP8B1 ;
Synthesis of bile acids and bile salts via 24-hydroxycholesterol	PID NCI	20%	0.00318	0.03139	CYP46A1 ; CYP8B1 ;
G alpha (q) signalling events	REACTOME	8%	0.00422	0.03939	GNG2 ; PLCB1 ; PLCB4 ;
Chemokine signaling pathway	KEGG	3%	0.00505	0.04462	ADCY5 ; GNG2 ; JAK2 ; PLCB1 ; PLCB4 ; PREX1 ;
IL2	NETPATH	5%	0.00520	0.04368	CCNE1 ; JAK2 ; MAP2K2 ; PLCB1 ;
Cyclin E associated events during G1/S transition	PID NCI	15%	0.00541	0.04332	CCNE1 ; CDK7 ;
Salivary secretion	KEGG	5%	0.00543	0.04147	ADCY5 ; PLCB1 ; PLCB4 ; RYR3 ;
Mitotic Prometaphase	PID NCI	5%	0.00591	0.04320	CDCA8 ; MAD1L1 ; SEH1L ; ZWINT ;
Cyclin E associated events during G1/S transition	REACTOME	14%	0.00628	0.04397	CCNE1 ; CDK7 ;
Nucleotide excision repair	KEGG	7%	0.00639	0.04291	CDK7 ; POLE ; XPA ;
ErbB signaling pathway	KEGG	5%	0.00643	0.03998	CBLB ; MAP2K2 ; PAK7 ; PLCG2 ;
Gap junction	KEGG	5%	0.00643	0.03998	ADCY5 ; MAP2K2 ; PLCB1 ; PLCB4 ;
TCR	NETPATH	3%	0.00647	0.03885	ANXA2 ; BCL10 ; CBLB ; HGS ; MAP2K2 ; PHLDB3 ; PLCG2 ;
Mitotic Prometaphase	REACTOME	5%	0.00669	0.03876	CDCA8 ; MAD1L1 ; SEH1L ; ZWINT ;
PLC beta mediated events	REACTOME	13%	0.00721	0.04037	PLCB1 ; PLCB4 ;
Cadmium induces dna synthesis and proliferation in macrophages	PID BIOCARTA	13%	0.00819	0.04301	MAP2K2 ; PLCB1 ;
Primary bile acid biosynthesis	KEGG	13%	0.00819	0.04301	CYP46A1 ; CYP8B1 ;
CXCR4-mediated signaling events	PID NCI	6%	0.00911	0.04636	GNG2 ; HGS ; JAK2 ;
GnRH signaling pathway	KEGG	4%	0.01011	0.04855	ADCY5 ; MAP2K2 ; PLCB1 ; PLCB4 ;
Melanogenesis	KEGG	4%	0.01011	0.04855	ADCY5 ; MAP2K2 ; PLCB1 ; PLCB4 ;
Ligand-gated ion channel transport	REACTOME	11%	0.01033	0.04820	GABRR1 ; GABRR2 ;
Resolution of Sister Chromatid Cohesion	REACTOME	4%	0.01084	0.04921	CDCA8 ; MAD1L1 ; SEH1L ; ZWINT ;
Dual incision reaction in GG-NER	REACTOME	10%	0.01268	0.04956	CDK7 ; XPA ;
Dual incision reaction in GG-NER	PID NCI	10%	0.01268	0.04956	CDK7 ; XPA ;
Formation of incision complex in GG-NER	REACTOME	10%	0.01268	0.04956	CDK7 ; XPA ;
Formation of incision complex in GG-NER	PID NCI	10%	0.01268	0.04956	CDK7 ; XPA ;
G beta:gamma signalling through PLC beta	REACTOME	10%	0.01268	0.04956	GNG2 ; PLCB1 ;
Global Genomic NER (GG-NER)	PID NCI	10%	0.01268	0.04956	CDK7 ; XPA ;
Pkc-catalyzed phosphorylation of inhibitory phosphoprotein of myosin phosphatase	PID BIOCARTA	10%	0.01394	0.04984	CACNA1A ; PLCB1 ;
Presynaptic function of Kainate receptors	REACTOME	10%	0.01394	0.04984	GNG2 ; PLCB1 ;
Signal transduction by L1	PID NCI	10%	0.01394	0.04984	MAP2K2 ; NCAM1 ;

Table S5. Correlations between host coding genomic sites and microbiome taxa by body site

Body Site	Chrom	Position [1]	SNP [2]	P-value [3]	R^2	Type	Annotation [4]	FDR Q-value	Bacteria
**Anterior_nares**	2	24290671	rs61742149	1.13E-05	0.018	synonymous	SF3B14:NM_016047:exon4:c.C339T:p.L113L,	0.08990352	Root.p__Firmicutes.c__Bacilli
	10	129921195	rs1063536	1.07E-05	0.063	synonymous	MKI67:NM_001145966:exon4:c.T237A:p.P79P,MKI67:NM_002417:exon4:c.T237A:p.P79P,	0.08990352	Root.p__Firmicutes.c__Bacilli
	14	93276654	rs1040835	1.70E-06	0.092	nonsynonymous	GOLGA5:NM_005113:exon5:c.T1048C:p.F350L,	0.04038523	Root.p__Firmicutes.c__Bacilli
	16	84522897	rs436278	8.09E-07	0.050	nonsynonymous	KIAA1609:NM_020947:exon4:c.C516G:p.D172E,	0.01925077	Root.p__Firmicutes.c__Clostridia.o__Clostridiales.f__Veillonellaceae
	13	78216873	rs1053985	3.51E-06	0.071	synonymous	SCEL:NM_001160706:exon30:c.A1854G:p.L618L,SCEL:NM_003843:exon31:c.A1920G:p.L640L, SCEL:NM_144777:exon32:c.A1980G:p.L660L,	0.08345806	Root.p__Proteobacteria
	2	186625770 - 186671912	multiple	2.41E-06 (min)	0.1339 (max)	syn+nonsyn	FSIP2:NM_173651:exons 10, 12,16,17	0.019-0.037	Root.p__Proteobacteria.c__Gammaproteobacteria.o__Pseudomonadales.f__Moraxellaceae
**Attached_Keratinized_gingiva**	6	80741243	rs1801465	2.94E-06	0.104	synonymous	TTK:NM_001166691:exon14:c.A1578T:p.I526I,TTK:NM_003318:exon14:c.A1581T:p.I527I,	0.06981808	Root.p__Actinobacteria.c__Actinobacteria.o__Actinomycetales.f__Actinomycetaceae.g__Actinomyces
	17	67125840	rs4968839	3.37E-07	0.099	nonsynonymous	ABCA6:NM_080284:exon7:c.G844A:p.V282I,	0.008009104	Root.p__Bacteroidetes.c__Bacteroidia.o__Bacteroidales.f__Prevotellaceae.g__Prevotella
	17	67212031	rs11077414	9.99E-07	0.084	synonymous	ABCA10:NM_080282:exon9:c.T783C:p.Y261Y,	0.01187811	Root.p__Bacteroidetes.c__Bacteroidia.o__Bacteroidales.f__Prevotellaceae.g__Prevotella
	3	174814920	rs9836841	4.33E-06	0.043	nonsynonymous	NAALADL2:NM_207015:exon2:c.A384G:p.I128M,	0.09829463	Root.p__Bacteroidetes.c__Flavobacteria.o__Flavobacteriales.f__Flavobacteriaceae.g__Capnocytophaga
	10	125528048	rs1219725	8.27E-06	0.056	synonymous	CPXM2:NM_198148:exon9:c.C1293T:p.Y431Y,	0.09829463	Root.p__Bacteroidetes.c__Flavobacteria.o__Flavobacteriales.f__Flavobacteriaceae.g__Capnocytophaga
	9	104309445	rs2273976	3.74E-07	0.096	synonymous	RNF20:NM_019592:exon8:c.T921C:p.Y307Y,	0.008884208	Root.p__Firmicutes.c__Bacilli.o__Gemellales.f__Gemellaceae.g__Gemella
	16	81398635	rs2608555	7.34E-06	0.042	synonymous	GAN:NM_022041:exon8:c.C1293T:p.Y431Y,	0.0872726	Root.p__Firmicutes.c__Bacilli.o__Gemellales.f__Gemellaceae.g__Gemella
	2	228230945	228230945	4.48E-06	0.187	nonsynonymous	TM4SF20:NM_024795:exon3:c.C265T:p.L89F,	0.05329098	Root.p__Firmicutes.c__Bacilli.o__Lactobacillales.f__Streptococcaceae.g__Streptococcus
	7	47840387	rs13231277	4.18E-06	0.160	nonsynonymous	PKD1L1:NM_138295:exon54:c.G8053A:p.A2685T,	0.05329098	Root.p__Firmicutes.c__Bacilli.o__Lactobacillales.f__Streptococcaceae.g__Streptococcus
	1	159824967	rs2501340	3.08E-06	0.088	nonsynonymous	C1orf204:NM_001134233:exon1:c.C61G:p.Q21E,	0.07317106	Root.p__Firmicutes.c__Clostridia.o__Clostridiales.f__Lachnospiraceae
	3	128344786	rs1126828	1.19E-06	0.113	synonymous	RPN1:NM_002950:exon7:c.C1206T:p.D402D,	0.02827442	Root.p__Proteobacteria.c__Gammaproteobacteria.o__Pasteurellales.f__Pasteurellaceae.g__Haemophilus
**Buccal_mucosa**	22	30689998	rs11089442	3.80E-06	0.117	synonymous	TBC1D10A:NM_031937:exon7:c.C807T:p.L269L,TBC1D10A:NM_001204240:exon7:c.C828T:p.L276L,	0.09026129	Root.p__Firmicutes.c__Clostridia.o__Clostridiales.f__ClostridialesFamilyXI.IncertaeSedis
	11	87013438	rs2276102	1.34E-06	0.001	nonsynonymous	TMEM135:NM_001168724:exon7:c.G586A:p.G196R,TMEM135:NM_022918:exon8:c.G652A:p.G218R,	0.03183874	Root.p__Firmicutes.c__Clostridia.o__Clostridiales.f__Lachnospiraceae.g__Moryella
	17	76157266	rs4071641	4.27E-06	0.023	nonsynonymous	C17orf99:NM_001163075:exon3:c.T301C:p.W101R,	0.05070658	Root.p__Firmicutes.c__Clostridia.o__Clostridiales.f__Lachnospiraceae.g__Moryella
	3	183211906	rs1520101	2.38E-06	0.025	synonymous	KLHL6:NM_130446:exon5:c.T1311C:p.N437N,	0.05661542	Root.p__Proteobacteria.c__Betaproteobacteria.o__Burkholderiales.f__Burkholderiaceae.g__Lautropia
	4	40337523	rs56241474	7.33E-07	0.027	synonymous	CHRNA9:NM_017581:exon1:c.C39T:p.I13I,	0.01743165	Root.p__Proteobacteria.c__Betaproteobacteria.o__Neisseriales.f__Neisseriaceae.g__Eikenella
**Hard_palate**	16	842450	rs2277897	3.70E-06	0.025	synonymous	CHTF18:NM_022092:exon11:c.C1338T:p.N446N,	0.08801875	Root.p__Firmicutes.c__Clostridia.o__Clostridiales.f__ClostridialesFamilyXIII.IncertaeSedis.g__Eubacterium
	19	10794630	rs2229383	4.07E-07	0.201	synonymous	ILF3:NM_012218:exon17:c.G2043T:p.S681S,ILF3:NM_001137673:exon17:c.G2055T:p.S685S,ILF3:NM_017620:exon17:c.G2055T:p.S685S,ILF3:NM_153464:exon17:c.G2043T:p.S681S,ILF3:NM_004516:exon17:c.G2043T:p.S681S,	0.009683965	Root.p__Firmicutes.c__Clostridia.o__Clostridiales.f__Veillonellaceae.g__Veillonella
	4	41673604	rs11734372	2.43E-06	0.083	nonsynonymous	LIMCH1:NM_001112720:exon11:c.T1778C:p.M593T,LIMCH1:NM_001112717:exon17:c.T2276C:p.M759T,LIMCH1:NM_014988:exon17:c.T2276C:p.M759T,LIMCH1:NM_001112718:exon17:c.T2276C:p.M759T,LIMCH1:NM_001112719:exon12:c.T1814C:p.M605T,	0.05768058	Root.p__Proteobacteria.c__Betaproteobacteria.o__Neisseriales.f__Neisseriaceae.g__Neisseria
**Left_Antecubital_fossa**	1	92457843	rs10747493	8.08E-07	0.015	nonsynonymous	BRDT:NM_207189:exon14:c.C2087T:p.P696L,BRDT:NM_001242810:exon14:c.C1868T:p.P623L, BRDT:NM_001726:exon14:c.C2087T:p.P696L,BRDT:NM_001242805:exon15:c.C2087T:p.P696L, BRDT:NM_001242807:exon13:c.C1949T:p.P650L,BRDT:NM_001242808:exon13:c.C1949T:p.P650L,BRDT:NM_001242806:exon14:c.C2099T:p.P700L,	0.01921567	Root.p__Firmicutes.c__Clostridia.o__Clostridiales.f__Ruminococcaceae.g__Faecalibacterium
	9	38615698	rs1832313	2.15E-06	0.008	nonsynonymous	ANKRD18A:NM_147195:exon3:c.G388A:p.E130K,	0.02555275	Root.p__Firmicutes.c__Clostridia.o__Clostridiales.f__Veillonellaceae
	15	100514614	rs2573652	5.76E-07	0.010	nonsynonymous	ADAMTS17:NM_139057:exon22:c.A3281G:p.N1094S,	0.01369865	Root.p__Firmicutes.c__Clostridia.o__Clostridiales.f__Veillonellaceae
	14	88693725	rs2277524	3.11E-06	0.004	synonymous	KCNK10:NM_138317:exon4:c.C675G:p.V225V,KCNK10:NM_021161:exon4:c.C660G:p.V220V,KCNK10:NM_138318:exon4:c.C675G:p.V225V,	0.07394847	Root.p__Fusobacteria.c__Fusobacteria.o__Fusobacteriales.f__Fusobacteriaceae.g__Fusobacterium
	2	163208893	rs17783344	3.67E-06	0.002	nonsynonymous	GCA:NM_012198:exon3:c.T238G:p.S80A,	0.08730721	Root.p__Proteobacteria.c__Alphaproteobacteria.o__Rhizobiales
**Left_Retroauricular_crease**	9	33442952	rs2228332	1.19E-06	0.023	synonymous	AQP3:NM_004925:exon4:c.C390T:p.F130F,	0.0283767	Root.p__Bacteroidetes.c__Bacteroidia.o__Bacteroidales.f__Bacteroidaceae.g__Bacteroides
	1	111862074	rs2820092	2.41E-06	0.114	synonymous	CHIA:NM_201653:exon11:c.C1161T:p.L387L,CHIA:NM_021797:exon8:c.C837T:p.L279L,	0.05720533	Root.p__Firmicutes.c__Clostridia.o__Clostridiales.f__Veillonellaceae.g__Veillonella
	12	10782115 - 10783729	rs3759259, rs2290717	6.79E-06	0.061	syn, nonsyn	STYK1:NM_018423:exon5, 6	0.097	Root.p__Proteobacteria.c__Gammaproteobacteria.o__Enterobacteriales.f__Enterobacteriaceae
	10	134017295	rs12313	2.54E-06	0.068	synonymous	DPYSL4:NM_006426:exon13:c.A1491G:p.G497G,	0.06029751	Root.p__Proteobacteria.c__Gammaproteobacteria.o__Pseudomonadales.f__Moraxellaceae
**Palatine_Tonsils**	17	78073355	rs56407805	2.92E-06	0.122	synonymous	CCDC40:NM_017950:exon20:c.A3210G:p.T1070T,	0.06950309	Root.p__Bacteroidetes.c__Bacteroidia.o__Bacteroidales.f__Prevotellaceae.g__Prevotella
	1	169512027	rs6021	1.51E-06	0.103	synonymous	F5:NM_000130:exon13:c.A2301G:p.S767S,	0.03599989	Root.p__Proteobacteria.c__Gammaproteobacteria.o__Pasteurellales.f__Pasteurellaceae
	4	155241572	rs11935573	2.52E-06	0.027	nonsynonymous	DCHS2:NM_017639:exon14:c.C3614T:p.S1205L,	0.06001567	Root.p__TM7.c__TM7-3
**Right_Retroauricular_crease**	6	34831866	rs3734264	7.71E-07	0.103	synonymous	UHRF1BP1:NM_017754:exon15:c.G3303A:p.T1101T,	0.01834216	Root.p__Firmicutes.c__Bacilli.o__Lactobacillales.f__Carnobacteriaceae.g__Granulicatella
	1	223116240	rs61746477	3.56E-07	0.168	synonymous	DISP1:NM_032890:exon4:c.G75A:p.P25P,	0.008468172	Root.p__Proteobacteria.c__Gammaproteobacteria.o__Pseudomonadales.f__Moraxellaceae
**Saliva**	17	40714804	rs615942	3.14E-07	0.038	nonsynonymous	COASY:NM_001042529:exon2:c.C164A:p.S55Y,COASY:NM_025233:exon1:c.C164A:p.S55Y,COASY:NM_001042532:exon3:c.C251A:p.S84Y,COASY:NM_001042530:exon2:c.C164A:p.S55Y,	0.007458662	Root.p__Bacteroidetes.c__Bacteroidia.o__Bacteroidales.f__Porphyromonadaceae.g__Porphyromonas
	10	64927823	rs1935	1.98E-06	0.195	nonsynonymous	JMJD1C:NM_004241:exon23:c.G6894C:p.E2298D,JMJD1C:NM_032776:exon26:c.G7605C:p.E2535D,	0.0469734	Root.p__Firmicutes.c__Clostridia.o__Clostridiales.f__Ruminococcaceae
	15	43632549	rs2278857	8.67E-06	0.058	synonymous	ADAL:NM_001159280:exon7:c.T342C:p.T114T,ADAL:NM_001012969:exon6:c.T342C:p.T114T,	0.06876747	Root.p__Fusobacteria.c__Fusobacteria.o__Fusobacteriales.f__Fusobacteriaceae.g__Leptotrichia
	15	43661802	rs3809482	1.04E-06	0.104	nonsynonymous	ZSCAN29:NM_152455:exon1:c.A310G:p.R104G,	0.02475914	Root.p__Fusobacteria.c__Fusobacteria.o__Fusobacteriales.f__Fusobacteriaceae.g__Leptotrichia
	15	43748304	rs690367	6.95E-06	0.067	synonymous	TP53BP1:NM_005657:exon12:c.T2487C:p.D829D,TP53BP1:NM_001141979:exon12:c.T2502C:p.D834D,TP53BP1:NM_001141980:exon12:c.T2502C:p.D834D,	0.06876747	Root.p__Fusobacteria.c__Fusobacteria.o__Fusobacteriales.f__Fusobacteriaceae.g__Leptotrichia
**Stool**	2	136511817	rs1050115	6.51E-06	0.027	synonymous	UBXN4:NM_014607:exon4:c.A303G:p.E101E,	0.07742343	Root.p__Actinobacteria.c__Actinobacteria.o__Bifidobacteriales.f__Bifidobacteriaceae.g__Bifidobacterium
	2	136561557	rs2304371	4.14E-06	0.025	synonymous	LCT:NM_002299:exon11:c.C4606T:p.L1536L,	0.07742343	Root.p__Actinobacteria.c__Actinobacteria.o__Bifidobacteriales.f__Bifidobacteriaceae.g__Bifidobacterium
	2	136590746	rs3754689	1.16E-05	0.033	nonsynonymous	LCT:NM_002299:exon2:c.G655A:p.V219I,	0.09173467	Root.p__Actinobacteria.c__Actinobacteria.o__Bifidobacteriales.f__Bifidobacteriaceae.g__Bifidobacterium
	3	129297223	rs1110168	6.92E-06	0.039	synonymous	PLXND1:NM_015103:exon9:c.G2295C:p.T765T,	0.05488223	Root.p__Bacteroidetes.c__Bacteroidia.o__Bacteroidales.f__Prevotellaceae.g__Prevotella
	11	57982620	rs1966834	4.52E-06	0.039	nonsynonymous	OR1S1:NM_001004458:exon1:c.A404G:p.H135R,	0.05488223	Root.p__Bacteroidetes.c__Bacteroidia.o__Bacteroidales.f__Prevotellaceae.g__Prevotella
	14	55907172	rs8019270	5.84E-06	0.040	nonsynonymous	TBPL2:NM_199047:exon1:c.G92C:p.R31P,	0.05488223	Root.p__Bacteroidetes.c__Bacteroidia.o__Bacteroidales.f__Prevotellaceae.g__Prevotella
	20	2816821	rs2274669	1.49E-06	0.065	synonymous	FAM113A:NM_022760:exon7:c.G981A:p.P327P,	0.03546493	Root.p__Bacteroidetes.c__Bacteroidia.o__Bacteroidales.f__Rikenellaceae.g__Alistipes
	7	37961001	rs10248138	1.34E-06	0.096	synonymous	EPDR1:NM_001242948:exon1:c.C34A:p.R12R,	0.03184945	Root.p__Firmicutes.c__Clostridia.o__Clostridiales.f__Lachnospiraceae.g__Lachnobacterium
**Subgingival_plaque**	10	73111408	rs780668	3.38E-06	0.048	nonsynonymous	SLC29A3:NM_018344:exon4:c.C473T:p.S158F,SLC29A3:NM_001174098:exon4:c.C473T:p.S158F,	0.08029831	Root.p__Firmicutes.c__Clostridia.o__Clostridiales.f__ClostridialesFamilyXI.IncertaeSedis
	2	216300482	rs1250259	2.89E-06	0.123	nonsynonymous	FN1:NM_212476:exon1:c.A44T:p.Q15L,FN1:NM_212478:exon1:c.A44T:p.Q15L,FN1:NM_002026:exon1:c.A44T:p.Q15L,FN1:NM_054034:exon1:c.A44T:p.Q15L,FN1:NM_212474:exon1:c.A44T:p.Q15L,FN1:NM_212482:exon1:c.A44T:p.Q15L,	0.06869464	Root.p__Firmicutes.c__Clostridia.o__Clostridiales.f__ClostridialesFamilyXIII.IncertaeSedis
	5	41049389	rs325863	1.19E-06	0.007	synonymous	HEATR7B2:NM_173489:exon14:c.A1494G:p.T498T,	0.01418358	Root.p__Fusobacteria.c__Fusobacteria.o__Fusobacteriales.f__Fusobacteriaceae.g__Fusobacterium
	5	41049397	rs325864	1.19E-06	0.007	nonsynonymous	HEATR7B2:NM_173489:exon14:c.G1486A:p.V496I,	0.01418358	Root.p__Fusobacteria.c__Fusobacteria.o__Fusobacteriales.f__Fusobacteriaceae.g__Fusobacterium
**Supragingival_plaque**	15	100821576	rs7496668	3.93E-06	0.091	nonsynonymous	ADAMTS17:NM_139057:exon4:c.C647T:p.S216L,	0.09343594	Root.p__Bacteroidetes.c__Bacteroidia.o__Bacteroidales.f__Porphyromonadaceae.g__Porphyromonas
	5	150696447	rs369907	1.08E-06	0.001	synonymous	SLC36A2:NM_181776:exon10:c.G1383A:p.Q461Q,	0.02559921	Root.p__Bacteroidetes.c__Bacteroidia.o__Bacteroidales.f__Porphyromonadaceae.g__Tannerella
	10	96954360	rs11188225	6.82E-06	0.089	nonsynonymous	C10orf129:NM_207321:exon2:c.T118A:p.C40S,	0.05406655	Root.p__Bacteroidetes.c__Bacteroidia.o__Bacteroidales.f__Porphyromonadaceae.g__Tannerella
	11	124096247	rs2466613	2.17E-06	0.004	nonsynonymous	OR8G2:NM_001007249:exon1:c.G850A:p.G284R,	0.02578936	Root.p__Bacteroidetes.c__Bacteroidia.o__Bacteroidales.f__Porphyromonadaceae.g__Tannerella
	3	52556890	rs4434138	9.53E-06	0.150	nonsynonymous	STAB1:NM_015136:exon62:c.A6844G:p.I2282V,	0.06613724	Root.p__Firmicutes.c__Bacilli.o__Lactobacillales.f__Streptococcaceae.g__Streptococcus
	3	52558133	rs13621	1.11E-05	0.142	synonymous	STAB1:NM_015136:exon68:c.T7560C:p.D2520D,	0.06613724	Root.p__Firmicutes.c__Bacilli.o__Lactobacillales.f__Streptococcaceae.g__Streptococcus
	3	52584787	rs2251219	7.98E-06	0.151	synonymous	PBRM1:NM_018313:exon28:c.A4335G:p.P1445P,	0.06613724	Root.p__Firmicutes.c__Bacilli.o__Lactobacillales.f__Streptococcaceae.g__Streptococcus
	3	52820981	rs678	1.40E-05	0.131	nonsynonymous	ITIH1:NM_001166434:exon12:c.A1328T:p.E443V,ITIH1:NM_001166435:exon10:c.A890T:p.E297V,ITIH1:NM_001166436:exon10:c.A890T:p.E297V,ITIH1:NM_002215:exon14:c.A1754T:p.E585V,	0.06641059	Root.p__Firmicutes.c__Bacilli.o__Lactobacillales.f__Streptococcaceae.g__Streptococcus
	3	52821011	rs1042779	4.46E-06	0.144	nonsynonymous	ITIH1:NM_001166434:exon12:c.A1358G:p.Q453R,ITIH1:NM_001166435:exon10:c.A920G:p.Q307R,ITIH1:NM_001166436:exon10:c.A920G:p.Q307R,ITIH1:NM_002215:exon14:c.A1784G:p.Q595R,	0.06613724	Root.p__Firmicutes.c__Bacilli.o__Lactobacillales.f__Streptococcaceae.g__Streptococcus
	1	231906589	rs2492367	5.80E-06	0.063	synonymous	DISC1:NM_001164539:exon6:c.C1407T:p.I469I,DISC1:NM_001164537:exon7:c.C1503T:p.I501I,DISC1:NM_001164548:exon6:c.C1407T:p.I469I,DISC1:NM_001164541:exon6:c.C1407T:p.I469I,DISC1:NM_001164544:exon6:c.C1407T:p.I469I,DISC1:NM_001164545:exon6:c.C1407T:p.I469I,DISC1:NM_001164549:exon6:c.C1407T:p.I469I,DISC1:NM_001164556:exon4:c.C357T:p.I119I,DISC1:NM_001164538:exon6:c.C1407T:p.I469I,DISC1:NM_001012959:exon6:c.C1407T:p.I469I,DISC1:NM_001164546:exon6:c.C1407T:p.I469I,DISC1:NM_018662:exon6:c.C1407T:p.I469I,DISC1:NM_001164542:exon6:c.C1407T:p.I469I,DISC1:NM_001164547:exon6:c.C1407T:p.I469I,DISC1:NM_001012957:exon6:c.C1407T:p.I469I,	0.068882562	Root.p__Firmicutes.c__Clostridia.o__Clostridiales.f__Lachnospiraceae.g__Moryella
	5	6600150	rs3822434	3.60E-07	0.113	synonymous	NSUN2:NM_017755:exon19:c.C2193T:p.D731D,NSUN2:NM_001193455:exon18:c.C2088T:p.D696D,	0.008549709	Root.p__Firmicutes.c__Clostridia.o__Clostridiales.f__Lachnospiraceae.g__Moryella
	5	150696447	rs369907	4.76E-06	0.012	synonymous	SLC36A2:NM_181776:exon10:c.G1383A:p.Q461Q,	0.05654645	Root.p__Firmicutes.c__Clostridia.o__Clostridiales.f__Veillonellaceae.g__Selenomonas
	8	21956108	rs35497596	3.08E-06	0.027	nonsynonymous	FAM160B2:NM_022749:exon7:c.A943G:p.T315A,	0.05654645	Root.p__Firmicutes.c__Clostridia.o__Clostridiales.f__Veillonellaceae.g__Selenomonas
	11	124096247	rs2466613	1.26E-05	0.004	nonsynonymous	OR8G2:NM_001007249:exon1:c.G850A:p.G284R,	0.09975057	Root.p__Firmicutes.c__Clostridia.o__Clostridiales.f__Veillonellaceae.g__Selenomonas
	3	52556890	rs4434138	1.17E-05	0.085	nonsynonymous	STAB1:NM_015136:exon62:c.A6844G:p.I2282V,	0.09254064	Root.p__Proteobacteria.c__Gammaproteobacteria.o__Pasteurellales.f__Pasteurellaceae.g__Aggregatibacter
	3	52558133	rs13621	1.05E-05	0.111	synonymous	STAB1:NM_015136:exon68:c.T7560C:p.D2520D,	0.09254064	Root.p__Proteobacteria.c__Gammaproteobacteria.o__Pasteurellales.f__Pasteurellaceae.g__Aggregatibacter
	11	64081445	rs2276014	1.12E-05	0.032	synonymous	ESRRA:NM_004451:exon3:c.G348A:p.P116P,	0.09254064	Root.p__Proteobacteria.c__Gammaproteobacteria.o__Pasteurellales.f__Pasteurellaceae.g__Aggregatibacter
**Throat**	4	121957559	rs34766411	2.26E-06	0.015	synonymous	NDNF:NM_024574:exon4:c.C1567T:p.L523L,	0.05365219	Root.p__Actinobacteria.c__Actinobacteria.o__Actinomycetales.f__Micrococcaceae.g__Rothia
	6	32410987	rs3135391	3.72E-06	0.006	synonymous	HLA-DRA:NM_019111:exon3:c.A354G:p.T118T,	0.08849282	Root.p__Firmicutes.c__Clostridia.o__Clostridiales.f__Veillonellaceae.g__Selenomonas
	10	11805354	rs12258196	1.46E-06	0.058	synonymous	ECHDC3:NM_024693:exon5:c.A723G:p.A241A,	0.03467416	Root.p__Firmicutes.c__Clostridia.o__Clostridiales.f__Veillonellaceae.g__Veillonella
	10	11805357	rs10906011	4.02E-06	0.067	synonymous	ECHDC3:NM_024693:exon5:c.G726A:p.S242S,	0.04778993	Root.p__Firmicutes.c__Clostridia.o__Clostridiales.f__Veillonellaceae.g__Veillonella
**Tongue_dorsum**	7	149474393	rs709061	1.58E-07	0.146	nonsynonymous	SSPO:NM_198455:exon4:c.A437G:p.Q146R,	0.003748043	Root.p__Firmicutes.c__Clostridia.o__Clostridiales.f__Lachnospiraceae.g__Oribacterium
	12	1893170	rs9805042	1.87E-06	0.039	synonymous	ADIPOR2:NM_024551:exon7:c.C963T:p.Y321Y,	0.04456747	Root.p__Firmicutes.c__Clostridia.o__Clostridiales.f__Peptococcaceae.g__Peptococcus
	4	38799710	rs4833095	5.04E-06	0.073	nonsynonymous	TLR1:NM_003263:exon4:c.A743G:p.N248S,	0.05994253	Root.p__Proteobacteria.c__Betaproteobacteria.o__Burkholderiales.f__Burkholderiaceae.g__Lautropia
	12	661656	rs7298766	3.45E-06	0.002	nonsynonymous	B4GALNT3:NM_173593:exon13:c.A1232G:p.K411R,	0.05994253	Root.p__Proteobacteria.c__Betaproteobacteria.o__Burkholderiales.f__Burkholderiaceae.g__Lautropia
	6	168709385	rs6925614	2.19E-08	0.181	nonsynonymous	DACT2:NM_214462:exon4:c.A1052G:p.E351G,	0.000521777	Root.p__Tenericutes

[1]Position is listed in hg19 genome coordinates. In case there are several positions within the same gene the range of positions was listed.

[2]RsID of SNP. In case rsID was not available, the position is listed. In case there are several rsIDs within the same gene the one with lowest P-value is listed.

[3]P-value of correlation (see Supplementary Methods). In case there were several sites within the same gene the minimal P-value is reported.

[4]For each correlated site, we list the appropriate ensembl transcripts, the exon, the protein position, and the amino acid change.

Table S6. Diseases and functional annotations enriched among genes with coding SNPs that are correlated with bacterial taxa abundance.

Category	Diseases or Functions Annotation	P-Value	Molecules	# Molecules
Cancer	melanoma	1.41E-05	ABCA10,ABCA6,ADAMTS17,ANKRD18A,B4GALNT3,BRDT,C10orf129,CCDC40,CHIA,CHRNA9,CPXM2,DCHS2,DISC1,DISP1,F5,FN1,FSIP2,GOLGA5,HLA-DRA,ILF3,ITIH1,JMJD1C,KCNK10,KLHL6,LCT,LIMCH1,MKI67,MROH2B,NAALADL2,NDNF,PKD1L1,PLXND1,RNF20,RPN1,SLC36A2,STAB1,STYK1,TBC1D10A,TLDC1,TLR1,TMEM135,TP53BP1,TTK,UBXN4,ZSCAN29	45
Cellular Movement	transendothelial migration of lymphocytes	8.75E-05	AQP3,FN1,STAB1	3
Hematological System Development and Function	transendothelial migration of lymphocytes	8.75E-05	AQP3,FN1,STAB1	3
Immune Cell Trafficking	transendothelial migration of lymphocytes	8.75E-05	AQP3,FN1,STAB1	3
Cellular Movement	migration of pyramidal neurons	1.09E-04	DISC1,FN1	2
Nervous System Development and Function	migration of pyramidal neurons	1.09E-04	DISC1,FN1	2
Tissue Morphology	lack of blood island	8.36E-04	F5,FN1	2
Dermatological Diseases and Conditions	psoriasiform dermatitis	9.74E-04	FN1,MKI67	2
Inflammatory Disease	psoriasiform dermatitis	9.74E-04	FN1,MKI67	2
Cellular Movement	transendothelial migration of T lymphocytes	2.01E-03	AQP3,STAB1	2
Hematological System Development and Function	transendothelial migration of T lymphocytes	2.01E-03	AQP3,STAB1	2
Immune Cell Trafficking	transendothelial migration of T lymphocytes	2.01E-03	AQP3,STAB1	2
Cell-mediated Immune Response	transendothelial migration of T lymphocytes	2.01E-03	AQP3,STAB1	2
Inflammatory Disease	Meningitis	2.89E-03	F5,TLR1	2
Neurological Disease	Meningitis	2.89E-03	F5,TLR1	2
Cancer	growth of mammary tumor	5.65E-03	ILF3,MKI67,TP53BP1	3
Organismal Injury and Abnormalities	growth of mammary tumor	5.65E-03	ILF3,MKI67,TP53BP1	3
Reproductive System Disease	growth of mammary tumor	5.65E-03	ILF3,MKI67,TP53BP1	3
Gastrointestinal Disease	Sjogren's syndrome	5.80E-03	FN1,MKI67,TLR1	3
Ophthalmic Disease	Sjogren's syndrome	5.80E-03	FN1,MKI67,TLR1	3
Immunological Disease	Sjogren's syndrome	5.80E-03	FN1,MKI67,TLR1	3
Cardiovascular System Development and Function	binding of endothelial cells	5.80E-03	F5,FN1,STAB1	3
Cell-To-Cell Signaling and Interaction	binding of endothelial cells	5.80E-03	F5,FN1,STAB1	3
Tissue Morphology	abnormal morphology of vascular smooth muscle	6.09E-03	FN1,PLXND1	2
Cardiovascular System Development and Function	abnormal morphology of vascular smooth muscle	6.09E-03	FN1,PLXND1	2
Lymphoid Tissue Structure and Development	abnormal morphology of vascular smooth muscle	6.09E-03	FN1,PLXND1	2
Organ Morphology	abnormal morphology of vascular smooth muscle	6.09E-03	FN1,PLXND1	2
Organismal Development	abnormal morphology of vascular smooth muscle	6.09E-03	FN1,PLXND1	2
Skeletal and Muscular System Development and Function	abnormal morphology of vascular smooth muscle	6.09E-03	FN1,PLXND1	2
Cancer	Cancer	8.23E-03	ABCA10,ABCA6,ADAMTS17,ANKRD18A,AQP3,B4GALNT3,BRDT,C10orf129,CCDC40,CHIA,CHRNA9,CPXM2,DACT2,DCHS2,DISC1,DISP1,ESRRA,F5,FN1,FSIP2,GAN,GOLGA5,HLA-DRA,ILF3,ITIH1,JMJD1C,KCNK10,KLHL6,LCT,LIMCH1,MKI67,MROH2B,NAALADL2,NDNF,NSUN2,OR1S1,PBRM1,PKD1L1,PLXND1,RNF20,RPN1,SCEL,SLC36A2,STAB1,STYK1,TBC1D10A,TBPL2,TLDC1,TLR1,TMEM135,TP53BP1,TTK,UBXN4,ZSCAN29	54
Cell Death and Survival	cell viability of embryonic cells	9.91E-03	FN1,TP53BP1	2
Embryonic Development	cell viability of embryonic cells	9.91E-03	FN1,TP53BP1	2
Cell-To-Cell Signaling and Interaction	adhesion of kidney cell lines	1.08E-02	FN1,PLXND1	2
Tissue Development	adhesion of kidney cell lines	1.08E-02	FN1,PLXND1	2
Renal and Urological System Development and Function	adhesion of kidney cell lines	1.08E-02	FN1,PLXND1	2
Hematological System Development and Function	binding of blood platelets	1.17E-02	F5,FN1	2
Cell-To-Cell Signaling and Interaction	binding of blood platelets	1.17E-02	F5,FN1	2
Inflammatory Response	binding of blood platelets	1.17E-02	F5,FN1	2
Cancer	endocrine gland tumor	1.18E-02	DPYSL4,ESRRA,FN1,GOLGA5,HLA-DRA,MKI67	6
Cellular Development	differentiation of keratinocytes	1.36E-02	AQP3,FN1,SCEL	3
Hair and Skin Development and Function	differentiation of keratinocytes	1.36E-02	AQP3,FN1,SCEL	3
Carbohydrate Metabolism	release of carbohydrate	1.41E-02	ESRRA,FN1	2
Cell-To-Cell Signaling and Interaction	adhesion of fibroblast cell lines	1.61E-02	FN1,PLXND1	2
Connective Tissue Development and Function	adhesion of fibroblast cell lines	1.61E-02	FN1,PLXND1	2
Tissue Development	adhesion of fibroblast cell lines	1.61E-02	FN1,PLXND1	2
Cardiovascular System Development and Function	binding of vascular endothelial cells	1.61E-02	FN1,STAB1	2
Cell-To-Cell Signaling and Interaction	binding of vascular endothelial cells	1.61E-02	FN1,STAB1	2
Cellular Movement	migration of vascular endothelial cells	1.64E-02	FN1,PLXND1,STAB1	3
Cardiovascular System Development and Function	migration of vascular endothelial cells	1.64E-02	FN1,PLXND1,STAB1	3
Cancer	gastrointestinal adenocarcinoma	1.76E-02	ABCA6,DACT2,ESRRA,F5,FN1,GAN,JMJD1C,KCNK10,LCT,LIMCH1,MKI67,NSUN2,PKD1L1,RPN1,SCEL,STAB1,STYK1,TP53BP1,TTK	19
Gastrointestinal Disease	gastrointestinal adenocarcinoma	1.76E-02	ABCA6,DACT2,ESRRA,F5,FN1,GAN,JMJD1C,KCNK10,LCT,LIMCH1,MKI67,NSUN2,PKD1L1,RPN1,SCEL,STAB1,STYK1,TP53BP1,TTK	19
Cancer	adenocarcinoma	1.89E-02	ABCA10,ABCA6,BRDT,CCDC40,CHRNA9,DACT2,DISC1,ESRRA,F5,FN1,GAN,GOLGA5,HLA-DRA,JMJD1C,KCNK10,LCT,LIMCH1,MKI67,MROH2B,NSUN2,OR1S1,PBRM1,PKD1L1,RNF20,RPN1,SCEL,SLC36A2,STAB1,STYK1,TLR1,TP53BP1,TTK,ZSCAN29	33
Nervous System Development and Function	morphology of neurites	1.93E-02	DISC1,DPYSL4,GAN	3
Tissue Morphology	morphology of neurites	1.93E-02	DISC1,DPYSL4,GAN	3
Cell Morphology	morphology of neurites	1.93E-02	DISC1,DPYSL4,GAN	3
Cardiovascular Disease	Heart Disease	2.52E-02	CHRNA9,DISP1,ESRRA,F5,FN1,PKD1L1,PLXND1,RNF20,STAB1	9
Cardiovascular Disease	congenital heart disease	2.54E-02	PKD1L1,PLXND1,RNF20	3
Developmental Disorder	congenital heart disease	2.54E-02	PKD1L1,PLXND1,RNF20	3
Cellular Movement	transmigration of phagocytes	2.54E-02	FN1,STAB1	2
Hematological System Development and Function	transmigration of phagocytes	2.54E-02	FN1,STAB1	2
Immune Cell Trafficking	transmigration of phagocytes	2.54E-02	FN1,STAB1	2
Inflammatory Response	transmigration of phagocytes	2.54E-02	FN1,STAB1	2
Cancer	colon adenocarcinoma	2.56E-02	DACT2,ESRRA,F5,FN1,GAN,JMJD1C,KCNK10,LCT,LIMCH1,MKI67,NSUN2,PKD1L1,RPN1,SCEL,STAB1,STYK1,TP53BP1,TTK	18
Gastrointestinal Disease	colon adenocarcinoma	2.56E-02	DACT2,ESRRA,F5,FN1,GAN,JMJD1C,KCNK10,LCT,LIMCH1,MKI67,NSUN2,PKD1L1,RPN1,SCEL,STAB1,STYK1,TP53BP1,TTK	18
Infectious Disease	flu	2.67E-02	FN1,PLXND1	2
Cellular Movement	innervation of neurons	2.87E-02	CHRNA9,GAN	2
Nervous System Development and Function	innervation of neurons	2.87E-02	CHRNA9,GAN	2
Cell Morphology	innervation of neurons	2.87E-02	CHRNA9,GAN	2
Cancer	proliferation of mammary tumor cells	2.87E-02	MKI67,TP53BP1	2
Cellular Development	proliferation of mammary tumor cells	2.87E-02	MKI67,TP53BP1	2
Cellular Growth and Proliferation	proliferation of mammary tumor cells	2.87E-02	MKI67,TP53BP1	2
Organismal Injury and Abnormalities	proliferation of mammary tumor cells	2.87E-02	MKI67,TP53BP1	2
Reproductive System Disease	proliferation of mammary tumor cells	2.87E-02	MKI67,TP53BP1	2
Tumor Morphology	proliferation of mammary tumor cells	2.87E-02	MKI67,TP53BP1	2
Cancer	gastric carcinoma	2.89E-02	ABCA6,MKI67,TTK	3
Gastrointestinal Disease	gastric carcinoma	2.89E-02	ABCA6,MKI67,TTK	3
Cancer	head and neck tumor	2.93E-02	CPXM2,DPYSL4,ESRRA,F5,FN1,GOLGA5,MKI67,RNF20	8
Tissue Development	adhesion of extracellular matrix	2.94E-02	FN1,PLXND1	2
Cellular Assembly and Organization	retraction of cellular protrusions	3.00E-02	FN1,PLXND1	2
Cellular Compromise	retraction of cellular protrusions	3.00E-02	FN1,PLXND1	2
Cardiovascular Disease	ischemic cardiomyopathy	3.07E-02	CHRNA9,F5	2
Nervous System Development and Function	morphology of dendrites	3.07E-02	DISC1,DPYSL4	2
Tissue Morphology	morphology of dendrites	3.07E-02	DISC1,DPYSL4	2
Cell Morphology	morphology of dendrites	3.07E-02	DISC1,DPYSL4	2
Cancer	thyroid cancer	3.32E-02	ESRRA,FN1,GOLGA5	3
Endocrine System Disorders	thyroid cancer	3.32E-02	ESRRA,FN1,GOLGA5	3
Cellular Movement	migration of macrophages	3.35E-02	PLXND1,STAB1	2
Hematological System Development and Function	migration of macrophages	3.35E-02	PLXND1,STAB1	2
Immune Cell Trafficking	migration of macrophages	3.35E-02	PLXND1,STAB1	2
Inflammatory Response	migration of macrophages	3.35E-02	PLXND1,STAB1	2
Dermatological Diseases and Conditions	leprosy	3.43E-02	ILF3,TLR1	2
Infectious Disease	leprosy	3.43E-02	ILF3,TLR1	2
Inflammatory Disease	polyarticular juvenile rheumatoid arthritis	3.43E-02	HLA-DRA,TLR1	2
Connective Tissue Disorders	polyarticular juvenile rheumatoid arthritis	3.43E-02	HLA-DRA,TLR1	2
Skeletal and Muscular Disorders	polyarticular juvenile rheumatoid arthritis	3.43E-02	HLA-DRA,TLR1	2
Immunological Disease	polyarticular juvenile rheumatoid arthritis	3.43E-02	HLA-DRA,TLR1	2
Cellular Assembly and Organization	quantity of plasma membrane projections	3.50E-02	DPYSL4,FN1	2
Cellular Function and Maintenance	quantity of plasma membrane projections	3.50E-02	DPYSL4,FN1	2
Nervous System Development and Function	abnormal morphology of ventral root	3.61E-02	GAN	1
Cancer	colon cancer	3.63E-02	AQP3,DACT2,ESRRA,F5,FN1,GAN,JMJD1C,KCNK10,LCT,LIMCH1,MKI67,NSUN2,PKD1L1,RPN1,SCEL,STAB1,STYK1,TP53BP1,TTK	19
Gastrointestinal Disease	colon cancer	3.63E-02	AQP3,DACT2,ESRRA,F5,FN1,GAN,JMJD1C,KCNK10,LCT,LIMCH1,MKI67,NSUN2,PKD1L1,RPN1,SCEL,STAB1,STYK1,TP53BP1,TTK	19
Cancer	head and neck cancer	3.69E-02	CPXM2,ESRRA,F5,FN1,GOLGA5,MKI67,RNF20	7
Cancer	epithelial neoplasia	3.97E-02	ABCA10,ABCA6,BRDT,CCDC40,CHRNA9,CPXM2,DACT2,DCHS2,DISC1,DPYSL4,ESRRA,F5,FN1,GAN,GOLGA5,HLA-DRA,JMJD1C,KCNK10,LCT,LIMCH1,MKI67,MROH2B,NAALADL2,NSUN2,OR1S1,PBRM1,PKD1L1,RNF20,RPN1,SCEL,SLC36A2,STAB1,STYK1,TBPL2,TLR1,TP53BP1,TTK,ZSCAN29	38
Tissue Morphology	abnormal morphology of pericardium	4.02E-02	DISP1,FN1	2
Cardiovascular System Development and Function	abnormal morphology of pericardium	4.02E-02	DISP1,FN1	2
Organ Morphology	abnormal morphology of pericardium	4.02E-02	DISP1,FN1	2
Organismal Development	abnormal morphology of pericardium	4.02E-02	DISP1,FN1	2
Embryonic Development	development of somites	4.02E-02	DISP1,F5	2
Organismal Development	development of somites	4.02E-02	DISP1,F5	2
Tissue Development	development of somites	4.02E-02	DISP1,F5	2
Cellular Movement	migration of prostate cancer cell lines	4.02E-02	ESRRA,FN1	2
Inflammatory Response	antibacterial response	4.10E-02	STAB1,TLR1	2
Antimicrobial Response	antibacterial response	4.10E-02	STAB1,TLR1	2
Cancer	papillary thyroid cancer	4.10E-02	FN1,GOLGA5	2
Endocrine System Disorders	papillary thyroid cancer	4.10E-02	FN1,GOLGA5	2
Inflammatory Response	antimicrobial response	4.21E-02	CHIA,STAB1,TLR1	3
Antimicrobial Response	antimicrobial response	4.21E-02	CHIA,STAB1,TLR1	3
Tissue Development	organization of extracellular matrix	4.25E-02	FN1,NDNF	2
Dermatological Diseases and Conditions	lichen planus	4.49E-02	HLA-DRA,PLXND1	2
Inflammatory Disease	lichen planus	4.49E-02	HLA-DRA,PLXND1	2
Immunological Disease	lichen planus	4.49E-02	HLA-DRA,PLXND1	2
Cellular Movement	cell movement of T lymphocytes	4.53E-02	AQP3,FN1,STAB1	3
Hematological System Development and Function	cell movement of T lymphocytes	4.53E-02	AQP3,FN1,STAB1	3
Immune Cell Trafficking	cell movement of T lymphocytes	4.53E-02	AQP3,FN1,STAB1	3
Cell-mediated Immune Response	cell movement of T lymphocytes	4.53E-02	AQP3,FN1,STAB1	3
Cellular Movement	migration of breast cancer cell lines	4.63E-02	ESRRA,FN1,ILF3	3
Cancer	glioblastoma cancer	4.77E-02	FN1,MKI67,RNF20	3
Neurological Disease	glioblastoma cancer	4.77E-02	FN1,MKI67,RNF20	3
Cancer	hemangioma	4.90E-02	MKI67,STAB1	2

Table S7. Interaction networks enriched among genes with coding SNPs that are correlated with bacterial taxa abundance.

ID	Molecules in Network	Score	Focus Molecules	Top Diseases and Functions
1	Akt,ANGPTL1,AQP3,Cg,CHIA,CLEC4A,CYP4Z1,DISC1,ERK1/2,ESRRA,FAM160B2,FN1,HLA-DRA,ILF3,Immunoglobulin,ITIH1,LIMCH1,MIR320,MKI67,PCED1A,PI3K (complex),PLXND1,Prl4a1,RPN1,SBSN,Sema6d,SF3B14,STAB1,STYK1,TAS1R1,TLR1,TTK,Vegf,Vegfr dimer,Ybx1-ps3	42	19	Cellular Movement, Hematological System Development and Function, Immune Cell Trafficking
2	ACPL2,ALG13,ANKRD18A,ANXA9,ATN1,ATP1B2,BRDT,COASY,DISP1,DPYSL4,FAM101B,FGF1,GJC1,KAT6B,KCNK10,KLHL6,LPCAT1,MAP7D1,MOBP,MURC,NAGLU,NDNF,PRICKLE1,SLC9A2,SSPO,TAGLN3,TEP1,TLDC1,TMEM135,TTC28,UBC,UBXN4,UHRF1BP1,ZSCAN29,ZSWIM8	29	14	Cell Morphology, Cell Signaling, Cellular Compromise
3	ABCA10,ADIPOR2,AMZ2,ATP13A3,CALR,CANX,CAPN15,CD1D-CANX-CALR,CHRNA9,COPS5,DACT2,DCHS2,ECHDC3,F5,FAM114A2,GIN1,JMJD1C,LCT,MICU2,miR-483-3p (miRNAs w/seed CACUCCU),NIPA2,OARD1,RNF20,SLC13A3,SLC26A11,SLC35A5,TBC1D10A,TGFB1,TMEM106C,TMEM184B,TP53BP1,TVP23B,UBC,VAT1L,ZNF554	23	12	Cell Cycle, Cancer, Cellular Development
4	ABCA6,APP,ARL11,ARL6IP6,ARNT2,ATAT1,BHLHB9,CCDC40,CHTF18,CPXM2,CSF2,Csl,DNMT1,DPY19L3,E130116L18Rik,EPDR1,FAM32A,GCA,GOLGA5,GSAP,H2afv,Hist3h2ba,IL10,JAGN1,KCNE1L,NAALADL2,NRIP1,OCEL1,PSEN1,SCEL,SLC36A2,SLC6A11,SUN5,TICRR,ZFP36	18	10	Cell Death and Survival, Cell-To-Cell Signaling and Interaction, Inflammatory Response
5	ADAMTS17,CAMTA1,CCDC124,CCDC168,CEP78,CORO7/CORO7-PAM16,CUL1,DFNA5,DMD,EIF2AK2,FBXL20,FBXO22,FBXO34,FBXW9,GAN,GDAP1,MYOG,NSUN2,PBRM1,PKD1L1,PSMG3,RAP1GAP2,RPL10L,RPL3L,SCNN1D,SLC9A7,SP140,TBCB,TBPL2,TM4SF20,TTC32,TTC7B,UBC,UNC79,ZNF532	12	7	Developmental Disorder, Hereditary Disorder, Neurological Disease
