# Supplementary material for: Host genetic variation impacts microbiome composition across human body sites
Source: Genome Biol. 2015 Sep 15;16(1):191. doi: 10.1186/s13059-015-0759-1 (PMC4570153; doi:10.1186/s13059-015-0759-1)
Supplement: Supplementary file 3 — This is a PDF document containing detailed supplementary materials and methods. (PDF 300 kb) [file 13059_2015_759_MOESM3_ESM.pdf]

Additional file 3

Ran Blekhman[1,2,*], Julia K. Goodrich[3,4], Katherine Huang[5], Qi Sun[6], Robert Bukowski[6], Jordana T. Bell[7], Timothy D. Spector[7], Alon Keinan[8], Ruth E. Ley[3,4], Dirk Gevers[5,9], Andrew G. Clark[3]

1. Department of Genetics, Cell Biology, and Development, University of Minnesota, Minneapolis, MN 55455, USA
2. Department of Ecology, Evolution, and Behavior, University of Minnesota, St. Paul, MN 55108, USA
3. Department of Molecular Biology and Genetics, Cornell University, NY 14853, USA
4. Department of Microbiology, Cornell University, Ithaca, NY 14853, USA
5. Broad Institute of MIT and Harvard, Cambridge, MA 02142, USA
6. BRC Bioinformatics Facility, Institute of Biotechnology, Cornell University, Ithaca, NY 14853, USA
7. Department of Twin Research & Genetic Epidemiology, King's College London, U.K.
8. Department of Biological Statistics and Computational Biology, Cornell University, Ithaca, NY 14853, USA
9. Current address: Janssen Human Microbiome Institute, Janssen Research and Development, Cambridge, MA 02142

Correspondence should be addressed to Ran Blekhman (blekhman@umn.edu)

This document provides a complete and detailed description of the analyses in the paper. Therefore, there is some overlap with the text in the Methods section in the main text of the paper.

Ethical statement

Recruitment protocols were approved by Institutional Review Boards at each HMP clinical site, and written informed consent was obtained from all study participants for data sharing through dbGap. All study participants have consented for the sequencing of their own genetic material [1]. Specifically, the HMP human subjects study was reviewed by the Institutional Review Boards (IRBs) at each sampling site: the BCM (IRB protocols H-22895 (IRB no. 00001021) and H-22035 (IRB no. 00002649)); Washington University School of Medicine (IRB protocol HMP-07-001 (IRB no. 201105198)); and St Louis University (IRB no. 15778). The study was also reviewed by the J. Craig Venter Institute under IRB protocol 2008-084 (IRB no. 00003721), and at the Broad Institute of MIT and Harvard the study was determined to be exempt from IRB review.

Host read data acquisition, filtering, and alignment

The processing of the raw data files through the genotyping step was performed on the compute cluster at the Broad Institute. We downloaded 1,553 raw Illumina read files (total of 8 TB) in SRA format, representing samples from 98 individuals (HMP subjects), from the dbGaP database. The files were decrypted, and converted to FASTQ format using NCBI's SRA toolkit (version 1.0.0-b10) with default parameters. 152 files that failed the standard Illumina quality checks were excluded from the downstream analysis. The reads from the remaining 1401 files were aligned to the human genome (build hg19) using BWA v0.5.7 [2] with default settings for the alignment, except for the "bwa sampe" step, where the option "-a 2000" was used to change the maximum insert size from default 500 to 2000. Out of the 79,877,504,468 post-filter reads, 35,828,514,379 were mapped to the human genome.

The 1401 BAM files were reorganized by merging reads from different samples from the same subject into subject BAM files using samtools [3]. Each record was properly tagged with a Read Group (RG) reflecting the sequencing lane, the subject, the library of origin, and the sequencing platform. The merging failed for one individual (due to corruption of the original sample BAM files), and for 4 others the merged BAM files contained only reads from stools samples with virtually no human DNA present. These 5 subjects were excluded, leaving 93 individual BAM files with sizes varying from 7 GB through 142 GB, with the average size of 31.4 GB. The number of mapped reads per individual varied widely between 23 thousand and 840 million with a few individuals scoring as many as 1.5 billion mapped reads. The average number of mapped reads per individual was 365 million.

Further processing of the alignments was done using the GATK (build 5588) [4] and Picard (versions 1.41-1.46; http://picard.sourceforge.net/) packages. The GATK suite is very sensitive to the format of the input alignments. Therefore, in order to correct for a few

inconsistencies introduced by the BWA alignment program, each of the merged BAM files was subjected to the cleaning process using the CleanSam and FixMateInformation tools from the Picard package. The first of these tools soft-clips the alignments extending beyond the edge of a reference sequence, while the second synchronizes position information between reads in mate-pairs. Each subject BAM cleaned in this way was then run through the process of flagging the molecular duplicates and realignment around indels using the MarkDuplicates function from Picard and GATK's RealignerTargetCreator and IndelRealigner functions, respectively. The realignment step was followed by a call to Picard's FixMateInformation procedure. Overall, it took about 3,600 CPU hours to process all BAM files through the cleaning and MarkDuplicates step, and another 1,000 CPU hours for realignment around indels. These parts of the calculation were parallelized over individual BAM files (i.e., each individual BAM file was processed on a single cluster processor)

First pass genotype calling

Variants (SNPs and short indels) were called from all 93 cleaned and re-aligned BAM files using the GATK's UnifiedGenotyper function with standard emission confidence parameter set to 3.0 (-stand_emit_conf 3.0). This value, much lower than the GATK default, was used in order to provide an exhaustive list of possible variants for subsequent filtering. The coverage for each individual was down-sampled to 200 (i.e., the option –dcov 200 was used). Other options of UnifiedGenotyper were kept at their default values. The calculation was parallelized over genomic coordinates by splitting the genome into 80,000 bp intervals and running UnifiedGenotyper for each of these intervals on a separate processor of the compute cluster. Typically, each job took about 1 hour of wall time, with the exception of a few intervals around centromeric or telomeric regions, for which the calculation needed more time and sometimes had to be parallelized further in order to fit within the compute cluster queue time limits. Overall, the genotyping part of the calculation required about 4800 CPU-hours. After excluding contigs that did not map to a known chromosome, this unfiltered, low-pass genotype set included 19,377,382 SNPs and 3,519,487 short InDels.

Genotype call filtering, recalibration, and QC

In order to filter the genotype calls and keep only high-quality variants, we used GATK and applied several hard filters that are recommended for low-coverage whole-genome data [5]. Specifically, we excluded SNPs with low mapping quality (using the filter string "QUAL < 10"), SNPs with a strand bias ("SB >= -0.1"), and SNPs that are otherwise of low quality (using the filter string MQ0 >= 4 && ((MQ0 / (1.0 * DP)) > 0.1)"). In addition, we masked out SNPs that are near InDels using a window size of 10. Lastly, we excluded any SNPs for which there is missing information and a clear filter decision could not be made.

Next, we performed variant score recalibration on the SNPs that have passed the above filters using the GATK VariantRecalibrator. As input to train the model, we used three input SNP sets: (i) HapMap3.3 SNPs, with the options "known=false, training=true, truth=true,

prior=15.0”, (ii) dbSNP build 132 SNPs, with the options “known=true, training=false, truth=false, prior=8.0”, (iii) 1000 Genomes Project SNPs from Omni 2.5 chip, with the options “known=false, training=true, truth=false, prior=12.0”. After applying the recalibration using the GATK ApplyRecalibration command with the parameter “--ts_filter_level 99.0”, and excluding variants that did not pass the various filters, we were left with 13,190,940 SNPs across the 93 individuals. Of this set, 7,229,492 SNPs (60.3%) were also found in dbSNP.

As quality control, we plotted the number of sites filtered out by each filter or combination of filters, as well as the Ti/Tv ratio for each filter combination (Figure S6). The sites that passed our filtering criteria have the highest Ti/Tv ratio (mean 2.1), which is close to the expected value observed in many sequencing projects, including the 1000 Genomes Project pilot data (genomic average Ti/Tv of 1.96)[6]. In addition, we plotted the coverage across the set of filtered and recalibrated SNPs in a number of ways. First, we plotted the mean coverage per site summed across all individuals (Figure S2). The mean depth of coverage for each site is 1061 reads (median 1093), and 99% of sites are covered at >500x across 93 individuals. Next, we plotted the mean coverage for each individual (Figure S3). There is noticeable variability across individuals, with one individual covered at >50x and some <5x. However, most individuals have a mean coverage in the range of 5x-20x, and only two individuals have a mean coverage of <3x. Considering the number of SNPs called for each individual, we again see some variability (Figure S8), with the same two individuals with fewer SNPs (around 10M, compared to a mean of 12.9M across all individuals). Although this variability was expected, given that the total number of reads mapped varied greatly across individuals, we decided to exclude from the association analysis the two individuals with < 10M SNPs, to make sure the results are not biased by low-coverage calls.

When we consider the frequency spectrum of alleles in our sample (Figure S7), we see an enrichment of low-frequency variants, as consistent with many recent population-scale sequencing studies [7]. We see a similar distribution when we consider allele sharing across individuals (Figures S8 and S9), with most alleles appearing in only one individual. Since alleles at lower frequencies are less informative for association analysis, we excluded from downstream analysis SNPs that are at frequency of less than 5% in our sample, leaving us a set of 5,536,004 SNPs. Of this set, 5,108,016 SNPs are also found in dbSNP (92.3%).

Finally, we transformed the SNP data using the make-bed command in PLINK, keeping only SNPs with minor allele frequency above 10% (using the parameter --maf 0.1), SNP with P-value > 10^{-3} for Hardy-Weinberg equilibrium (HWE; using the parameter --hwe 0.001), autosomal SNPs, and SNPs with less than 50% missing information (using the parameter --geno 0.5). The final BED file included 4,205,323 SNPs that set that passed these QC thresholds and were used in the analysis.

Pairwise identity-by-state (IBS) distances between individuals were calculated from the filtered SNP data using PLINK (http://pngu.mgh.harvard.edu/~purcell/plink) [8]. We performed metric multidimensional scaling analysis (MDS) on the pairwise IBS distance matrix using PLINK.

Microbiome data acquisition and initial filtering and processing

OTU tables generated by the HMP consortium from sequencing of the 16S variable region 3-5 (V35) were downloaded from the HMP DACC website (http://hmpdacc.org/HMQCP) on June 21, 2012. For a detailed description of this dataset and the pipeline used to generate it see [1]. Briefly, the 16S sequences were processed by the HMP consortium using the QIIME pipeline [9-12], which uses an OTU-binning approach, and attaches a taxonomic classification for each OTU. The unfiltered table contained 45,383 rows (OTUs) and 4,790 columns (samples), representing samples taken from 18 (female) or 15 (male) body sites, including stool, saliva, tongue dorsum, hard palate, buccal mucosa, attached keratinized gingiva, palatine tonsils, throat, anterior nares, supragingival_plaque, subgingival plaque, left antecubital fossa, right antecubital fossa, left retroauricular crease, right retroauricular crease, vaginal introitus, mid vagina, and posterior fornix. First, we mapped the sample IDs in the OTU table to the IDs of the HMP individuals for which we have genotype data, and excluded columns in the OTU table that represent individuals without such information. The resulting table contained information for 2170 (45%) microbiome samples, representing the 93 genotyped individuals. We note that the number of individuals for which we had both genetic and genotype data varied across body sites (figure S4). Since we only had data for <40 female individuals, we excluded from further analyses the three female-specific body sites (vaginal introitus, mid vagina, and posterior fornix). We used QIIME [9] to calculate alpha and beta diversity and perform a Principal Coordinate Analysis (PCoA) using these OTU tables for each body site.

Host-bacteria Correlation analysis

We used the first 5 principal coordinates (PCs) of the microbiome 16S data in each of the 15 body sites as quantitative traits, which we correlated against genetic variation in the host. Prior to running this analysis we normalized the PC values using the Box-Cox transformation with the formula

$$y^{(\lambda)} = (y^{\lambda} - 1) / \lambda$$

where λ was calculated using the function box.cox.powers in R (in the package “car”).

Next, we filtered the SNP data using the make-bed command in PLINK, keeping only SNPs with minor allele frequency above 10% (using the parameter --maf 0.1), SNP with *P*-value > 10^{-3} for Hardy-Weinberg equilibrium (using the parameter --hwe 0.001), autosomal SNPs, and SNPs with less than 50% missing information (using the parameter --geno 0.5). The final BED file included 4,205,323 SNPs that set that passed these QC thresholds and were used in the analysis.

Correlation analysis of normalized trait values was performed in PLINK (http://pngu.mgh.harvard.edu/~purcell/plink) v1.07 [8]. We used a linear model (with the --linear parameter), and included the following covariates:

1. Individual sex (binary variable);

2. Individual age;
3. Site where microbiome data were collected. This was extracted from the “collectsite” column in the HMP map files, and was coded as a binary variable representing one of two collection centers (St. Louis and Houston);
4. Center where sequencing was performed. This was extracted from the “RUNCENTER” column in the HMP map files, and was coded as three binary variable representing the four sequencing centers: BCM (Baylor College of Medicine), BI (Broad Institute), JCVI (J. Craig Venter Institute), and WUGC (Washington University Genome Center). Note that some samples were sequenced in multiple centers.
5. The total number of sequences for each individual in the metagenomic sequencing data. This value was extracted from the OTU file for each body site, and represented here as a covariate instead of normalizing the trait values by this number prior to the analysis. Note that since this covariate has a different value for each body site, we created 15 body site-specific covariate files that were used in the association analysis.
6. The positions on the first five dimensions in the MDS analysis of the genotype data (see above).

Collapsing microbiome data by taxonomy

To test for correlations with specific microbiome taxa, we first aimed to use the taxonomic abundance data in the OTU table as quantitative traits. However, the dataset is large, with thousands of rows that can represent potential traits, many of which are intercorrelated and/or have identical taxonomic assignments. Thus, we aimed to reduce the dimensionality of the data by collapsing it by taxonomic assignment, and use filtering to include only informative traits. Using the procedure described below allowed us to reduce the number of traits tested to ~41 per body site on average.

First, we sought to collapse the OTU abundance data to represent single distinguishable phylogenetic taxa. The original OTU table contained taxonomic assignments for each row at the level of genus or above. We used this taxonomic assignment, and summed read counts over rows that correspond to identical taxa, using the complete taxonomic identification string. More specifically, we went over the unique phylogenetic assignments in each body site-specific OTU table. For each label (e.g., “Root;p__Firmicutes;c__Bacilli;o__Lactobacillales;f__Streptococcaceae;g__Streptococcus”) we identified all multi-level taxonomic assignments that correspond to its location in the phylogenetic tree as represented in the label string. In the example above, there are five taxonomic assignments:

(1) p__Firmicutes;c__Bacilli;o__Lactobacillales;f__Streptococcaceae;g__Streptococcus
(2) p__Firmicutes;c__Bacilli;o__Lactobacillales;f__Streptococcaceae
(3) p__Firmicutes;c__Bacilli;o__Lactobacillales
(4) p__Firmicutes;c__Bacilli

(5) p__Firmicutes

Overall, there were 742 unique taxa represented in the data, including 389 unique genera, 180 families, 100 orders, 47 classes, and 26 phyla.

For each taxon, we identified all the rows that correspond to it in the body site-specific OTU table, and summed across these rows, to achieve a single read count representing this taxon for each individual. By collapsing the data for each taxon in each body site, we were able to reduce the dimensionality of the data, as well as represent it in a phylogenetic context, with a single row representing each node in the underlying phylogenetic tree. We note that in most cases our collapsing method included only a single OTU from the original table, and >95% of taxa represent <10 OTUs from the original table. This process resulted in body site-specific tables of read counts where each row corresponds to a unique and single phylogenetic taxon. The number of taxa in each body site is shown in Figure S14. We found the largest number of taxa on the skin (at least 450 in each of the four body sites on the skin), and the fewest in the tongue dorsum and supragingival plaque with 228.

Microbiome data advanced filtering

Next, we used a number of filters to exclude uninformative traits from the association analysis. First, for each body site, we excluded taxa for which there is no data (zero reads) across individuals. Second, we excluded taxa for which data is sparse. This was achieved by counting, for each taxon, the proportion of zero-count cells, namely, the proportion of individuals for which there are no reads mapped to this taxon. When we plot these proportions across taxa (Figure S15), we see a clear bimodal distribution, where some taxa have a low proportion of zero counts, and some taxa have a high proportion of zero-counts. We used a cutoff of 50%, and excluded from further analysis all taxa for which half or more of the individuals have no reads assigned. The two steps above reduced the number of taxa considerably (Figure S14), ranging from an ~80% reduction in the number of taxa in the skin sites, to 57% in the tongue dorsum.

As another filtering measure, we wanted to exclude taxa for which data is highly correlated, as testing such taxa for association would be redundant and uninformative. We first calculated the pairwise Pearson correlation between each pair of taxa in each body site, and plotted the distribution of values (Figure S16). In all body sites there is a clear enrichment of very high pairwise correlations, with a peak around $r^2 = 1$. Figure S17 shows dotplots of the first 10 taxa in the GI tract, where high correlations between some taxon pairs are visible. To investigate this further, we clustered the taxa based on their pairwise correlations and displayed the results in a heatmap (Figure S18). There are multiple highly correlated clusters, each with a small number of taxa. Upon closer examination, we find that each of these clusters correspond to a group of taxa that represent consecutive levels in the phylogenetic tree. In other words, these high correlations are usually caused by a lower-ranked taxon that is driving the levels in a number of higher-ranked taxa. For example, Figure S19 shows a cluster (GI tract data) where the counts in the genus *Akkermansia* are driving the counts in all higher levels of the

phylogeny, up to the phylum level. Considering these observations, we decided to include only the lowest rank taxa from each highly correlated cluster in each body site. Specifically, we identified all pairs of taxa with an r^2 of at least 0.9, and excluded the more higher-ranked taxon of the pair from future analysis. This resulted in an exclusion of ~50% of remaining taxa (Figure S14).

Identifying interactions between host genetic variation and specific taxa

After the above filtering steps, our final set of traits included 615 taxa across the 15 body sites (see Figure S14), with an average of 41 taxa per body site. The variability in this number across body sites was not large, with a range of 25 taxa in the attached keratinized gingiva to 53 taxa in the Saliva. We filtered the genetic variation data to reduce the number of statistical test to include only protein-coding SNPs. We used ANNOVAR [13] to annotate the above set of filtered SNPs, and identified 33,814 protein-coding SNPs. We used this set of coding SNPs and ran the correlation analysis as described above against the taxon-level abundance data.

Enrichment analysis

Using the output of the correlation analysis, we considered SNPs with P-value $\leq 10^{-6}$, and identified genes that overlap or are located ≤ 50kb from these SNPs, using data for all known human genes taken from the refGene table (hg19 genome build), downloaded from the UCSC Genome Browser database in July 2012. The identified genes were used as input to functional enrichment analysis, performed using Ingenuity Pathway Analysis (IPA; August 2012 software release), a program that uses Ingenuity's high-quality knowledge base, which includes curated information on genes, pathways, and interactions (see www.ingenuity.com). IPA generates a P-value using a Fisher exact test comparing the expected and observed genes in a given pathway. The most enriched canonical pathways are listed in Table S1. To identify the bacterial taxa driving these enrichments, we calculated correlations between each OTU and the PCs in each body site. The most highly correlated OTU for each PC where correlation with host genetics was found is listed in Table S1. We also used the InnateDB database (www.innatedb.com) to identify enrichment of specific gene ontology (GO; www.geneontology.org [14]) categories (Table S3) and additional pathway databases (Table S4), including KEGG (www.genome.jp/kegg) [15] and Reactome (www.reactome.org).

To make sure the specific cutoff values chosen in this analysis do not affect the enrichment result, we repeated this analysis with varying P-value and gene distance cutoffs (see Table S2). Specifically, we used two P-value cutoffs ($P \leq 10^{-6}$ and $P \leq 5x10^{-7}$) and three gene distance cutoffs ($D \leq 50k$, $D \leq 20k$, and $D \leq 5k$), and examined the enrichment P-value and rank of pathways of interest (Table S2).

Figure 2 shows an enrichment analysis that includes data from the GWAS catalog [16], which was downloaded from www.genome.gov/26525384 in June 2013, and data generated by the GTEx consortium [17], which were downloaded from the GTEx portal

(www.broadinstitute.org/gtex/) on October 2013. The enrichment plots shown in Figure 2 were calculated as follows: given a dataset (e.g., GWAS catalog genes involved in obesity-related traits), and given a *P*-value cutoff (P_i, shown on the *x*-axis of the figure), we identified the set of genes or SNPs for which $P \leq P_i$. Next, we calculated the overlap between G_i and the genes or SNPs identified to be correlated with the microbiome in the current paper. The fold enrichment (*y*-axis) for P_i is the number observed compared to expected overlapping genes or SNPs, where the expected number is the overlap among genes or SNPs not in G_i.

To identify enrichment in an independent cohort, we used data from the TwinsUK Project, which included both stool microbiome 16S data, as well as host genetic data assessed by SNP genotyping, from 984 adults [18]. OTU tables and PCs were generated using the QIIME pipeline as described above. Host SNP genotyping data were fully imputed using IMPUTE version 2[19], and quality checked as previously described [20]. SNPs were removed if they had a minor allele frequency below 5%, a genotyping rate below 95% or extreme deviation from HWE ($p<0.001$). Deviation from HWE was determined using the genotypes from only a single twin from each twin pair. Only imputed SNPs with an imputation accuracy score (IMPUTE *INFO* field) greater than 0.9 were included in the analysis. The final number of SNPs used for the association analysis was 1,310,141. To test for correlation between host SNPs and fecal microbiome PCs, we used the score test implemented in the software Merlin [21] to account for the relatedness of the individuals (option –fastassoc). The recombination rates from HapMap II release 22 were used as the genetic map input to Merlin. Model covariates included the number of sequences per sample, sample batch, sequencing run, the person that extracted the DNA, the gender, the age, and the first 3 PCs of the MDS. After quality filtering of traits and genotypes, 170 MZ twin pairs, 241 DZ twin pairs, and 162 unrelated individuals were included in the association analysis. For the analysis shown in Figure 2C, we used correlation *P*-values for SNPs and nearby genes, and calculated fold-enrichment for several *P*-value as described above.

F_{ST} analysis

We used F_{ST} data downloaded from the database of recent positive selection across human populations [22] via http://jjwanglab.org/dbpshp in March 2014. We compared F_{ST} values in SNPs that were correlated with microbiome PCs with $P<10^{-4}$ in each of the four body sites and the rest of the SNPs in our sample. To compare two sets of F_{ST} values we used a permutation test on the medians as follows: we randomly split the data into two groups the same size of the two original groups, and calculated the difference in medians between the two groups. This process was repeated 10,000 times, and the *P*-value was defined as the proportion of permutations in which difference in medians was greater that the real difference between the two original groups. Figure 4 shows all the comparisons made and highlights in color cases where the calculated *P*-value was smaller than 10^{-3}. The error bars in the figure are 95% confidence intervals that were calculated using bootstrapping as follows: for a given set of F_{ST} values, we subsampled with replacement a sample of the same size, and calculated the median

of the sample. This was repeated 10,000 times, with the median recorded in each iteration. The 95% CI was defined as the range between the 2.5 and 97.5 percentiles of all subsample medians.

References

1. The Human Microbiome Project Consortium: **A framework for human microbiome research.** *Nature* 2012.
2. Li H, Durbin R: **Fast and accurate short read alignment with Burrows-Wheeler transform.** *Bioinformatics* 2009, **25:**1754-1760.
3. Li H, Handsaker B, Wysoker A, Fennell T, Ruan J, Homer N, Marth G, Abecasis G, Durbin R: **The Sequence Alignment/Map format and SAMtools.** *Bioinformatics* 2009, **25:**2078-2079.
4. McKenna A, Hanna M, Banks E, Sivachenko A, Cibulskis K, Kernytsky A, Garimella K, Altshuler D, Gabriel S, Daly M, DePristo MA: **The Genome Analysis Toolkit: a MapReduce framework for analyzing next-generation DNA sequencing data.** *Genome Res* 2010, **20:**1297-1303.
5. DePristo MA, Banks E, Poplin R, Garimella KV, Maguire JR, Hartl C, Philippakis AA, del Angel G, Rivas MA, Hanna M, et al: **A framework for variation discovery and genotyping using next-generation DNA sequencing data.** *Nat Genet* 2011, **43:**491-498.
6. Consortium TGP: **A map of human genome variation from population-scale sequencing.** *Nature* 2010, **467:**1061-1073.
7. Keinan A, Clark AG: **Recent explosive human population growth has resulted in an excess of rare genetic variants.** *Science* 2012, **336:**740-743.
8. Purcell S, Neale B, Todd-Brown K, Thomas L, Ferreira MA, Bender D, Maller J, Sklar P, de Bakker PI, Daly MJ, Sham PC: **PLINK: a tool set for whole-genome association and population-based linkage analyses.** *Am J Hum Genet* 2007, **81:**559-575.
9. Caporaso JG, Kuczynski J, Stombaugh J, Bittinger K, Bushman FD, Costello EK, Fierer N, Pena AG, Goodrich JK, Gordon JI, et al: **QIIME allows analysis of high-throughput community sequencing data.** *Nat Methods* 2010, **7:**335-336.
10. Price MN, Dehal PS, Arkin AP: **FastTree 2--approximately maximum-likelihood trees for large alignments.** *PLoS One* 2010, **5:**e9490.
11. Caporaso JG, Bittinger K, Bushman FD, DeSantis TZ, Andersen GL, Knight R: **PyNAST: a flexible tool for aligning sequences to a template alignment.** *Bioinformatics* 2010, **26:**266-267.
12. Edgar RC: **Search and clustering orders of magnitude faster than BLAST.** *Bioinformatics* 2010, **26:**2460-2461.
13. Wang K, Li M, Hakonarson H: **ANNOVAR: functional annotation of genetic variants from high-throughput sequencing data.** *Nucleic Acids Res* 2010, **38:**e164.
14. Consortium TGO: **The Gene Ontology in 2010: extensions and refinements.** *Nucleic Acids Res* 2010, **38:**D331-335.

15. Kanehisa M, Goto S, Sato Y, Furumichi M, Tanabe M: **KEGG for integration and interpretation of large-scale molecular data sets.** *Nucleic Acids Res* 2012, **40:**D109-114.
16. Hindorff LA, Sethupathy P, Junkins HA, Ramos EM, Mehta JP, Collins FS, Manolio TA: **Potential etiologic and functional implications of genome-wide association loci for human diseases and traits.** *Proc Natl Acad Sci U S A* 2009, **106:**9362-9367.
17. Consortium G: **The Genotype-Tissue Expression (GTEx) project.** *Nat Genet* 2013, **45:**580-585.
18. Goodrich JK, Blekhman R, Koren O, Beaumont M, Bell JT, Spector T, Clark AG, Ley RE: **Heritability and host SNP associations of microbial species in the human gut.** *In review* 2013.
19. Howie BN, Donnelly P, Marchini J: **A flexible and accurate genotype imputation method for the next generation of genome-wide association studies.** *PLoS Genet* 2009, **5:**e1000529.
20. Moayyeri A, Hammond CJ, Hart DJ, Spector TD: **The UK Adult Twin Registry (TwinsUK Resource).** *Twin Res Hum Genet* 2013, **16:**144-149.
21. Abecasis GR, Cherny SS, Cookson WO, Cardon LR: **Merlin--rapid analysis of dense genetic maps using sparse gene flow trees.** *Nat Genet* 2002, **30:**97-101.
22. Li MJ, Wang LY, Xia Z, Wong MP, Sham PC, Wang J: **dbPSHP: a database of recent positive selection across human populations.** *Nucleic Acids Res* 2014, **42:**D910-916.
